# [*closo*-B_10_H_8_-1-CN-10-Azinium]^−^ Anions: Photoactive Heteroditopic Ligands for Metal Complexes

**DOI:** 10.1021/acs.inorgchem.4c02670

**Published:** 2024-09-06

**Authors:** Rafał Jakubowski, Mustapha B. Abdulmojeed, Oleksandr Hietsoi, Andrienne C. Friedli, Piotr Kaszyński

**Affiliations:** † Department of Chemistry, Middle Tennessee State University, Murfreesboro, Tennessee 37132, United States; ‡ Centre of Molecular and Macromolecular Studies, Polish Academy of Sciences, Sienkiewicza 112, 90-363 Łódź, Poland; § Faculty of Chemistry, University of Łódź, Tamka 12, 91-403 Łódź, Poland

## Abstract

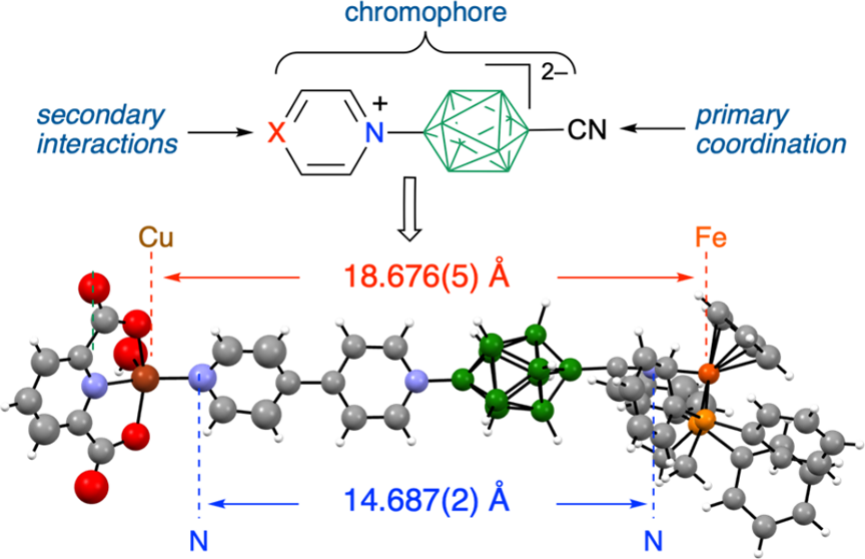

A new class of rigid, photoactive heteroditopic anionic ligands based on the 1,10-disubstituted [*closo*-B_10_H_10_]^2–^ anion was designed and six of these compounds were obtained from [*closo*-B_10_H_10_]^2–^ in three steps with yields in the range of 25–30%. The design includes two apical substituents, a metal coordinating cyano group and an azinium (4-cyanopyridinium, 4,4′-bipyridinium, pyrazinium, pyrimidinium, and pyridazinium), which provides a secondary binding site. The azinium substituent is involved in an efficient intramolecular charge transfer process and compensates one of the two delocalized negative charges of the boron cluster. The compounds exhibit intramolecular CT bands with maxima in the range of 340–410 nm (MeCN) and two are weakly fluorescent with significant Stokes shifts. A highly colored bis-zwitterionic byproduct with two clusters, [*closo*-1-CN-B_10_H_8_-10-(pyrazinium-1,4-diyl)-10′-B_10_H_8_-1′-CN-*closo*]^2–^, was also obtained in a low yield. The ligands were structurally characterized (XRD) and their geometrical and photophysical properties were compared to those of the analogues lacking the CN group and the parent pyridinium derivative. A comparative analysis of experimental data was augmented with DFT and TD-DFT results. Two of the ligands (with the 4,4′-bipyridinium and pyrazinium) were converted to (η^5^-Cp)(dppe)Fe complexes and one of them was used to obtain a heterodinuclear complex with Cu(pdc)(H_2_O)_3_ to demonstrate the ditopic function of the ligand. All three iron complexes were characterized by the XRD method.

## Abstract 2

Heteroditopic ligands [*closo*-B_10_H_8_-10-Azine-1-CN]^−^ with intramolecular CT are obtained in three steps and overall yields up to 30% and used to prepare three metal complexes demonstrating the ligand function.

## Introduction

The steric and electronic properties of carboranes^1^ and other *closo*-boranes are attractive factors in designing functional ligands^2−4^ for transition metal complexes acting as catalysts for coupling reactions,^5−7^ asymmetric reduction,^8^ hydrosilylation,^9^ olefin polymerization,^10,11^ luminescent materials,^12,13^ molecular objects,^14−16^ coordinational polymers,^17,18^ functional grids,^19^ and MOFs.^20−26^ The latter materials typically use homoditopic carborane carboxylic acids as ligands.

Among *closo*-boranes, the [*closo*-B_10_H_10_]^2–^ anion^27,28^ is exceptional due to its *D*_4d_ symmetry and high lying HOMO with large amplitudes at the apical (1 and 10) positions. Despite these features, anion [*closo*-B_10_H_10_]^2–^ has received little attention as a structural element of functional ligands besides our recent report^29^ and several examples of equatorial substituted derivatives.^30−34^

**Figure 1:**
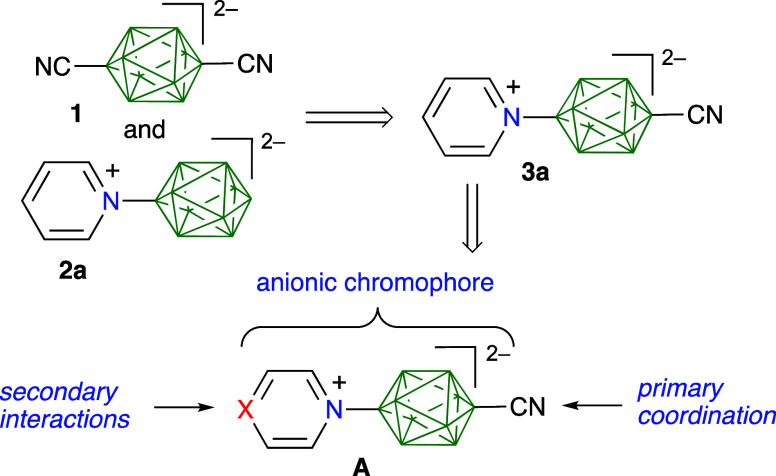
Conceptual development of a general design for linear, heteroditopic, anionic, photoactive ligands **A** by combining features of strongly binding ditopic ligand **1** with chromophore **2a**.

Recently, we demonstrated that [*closo*-B_10_H_8_-1,10-(CN)_2_]^2–^ (**1**, ref (35), Figure 1) is an efficient ligand for metal complexes due to the high electron density at the CN groups that is transferred from the {*closo*-B_10_} skeleton.^36^ The resulting HOMO extends over the entire molecule, providing effective communication between the two metal centers coordinated to the CN groups. Surprisingly, the strength of this communication is similar to that in the corresponding 1,4-dicyanobenzene complexes.^36^ We also demonstrated that [*closo*-B_10_H_9_-1-pyridinium]^−^ (**2a**) is a chromophore with photoinduced, intramolecular, cage-to-pyridine charge transfer (CT) with an absorption maximum at 365.5 nm in MeCN.^37^ The energy of this π–π* excitation can be tuned with substituents on both the pyridine and the {*closo*-B_10_} cluster.^37^

Substitution of a CN group at the other apical position of the cluster in **2a** leads to [*closo*-B_10_H_8_-1-CN-10-pyridinium]^−^ (**3a**),^37^ which uniquely combines the features of a strongly binding ligand with CT and a diffused overall negative charge. This constitutes a potentially attractive photoactive ligand for complexation of transition metals. Further modification of the azine heterocycle at the B(10) position in **3a** leads to the concept of heteroditopic photoactive ligands of the general structure **A** (Figure 1), in which the CN provides strong binding to a metal center, while the modified azine can, in principle, be used to build 2D and 3D networks through secondary interactions with other metal ions or carboxylic acids. Such functional polytopic^38^ and heteroditopic^39,40^ ligands are of continuous interest for *e.g*. MOFs.^41,42^

**Figure 2:**
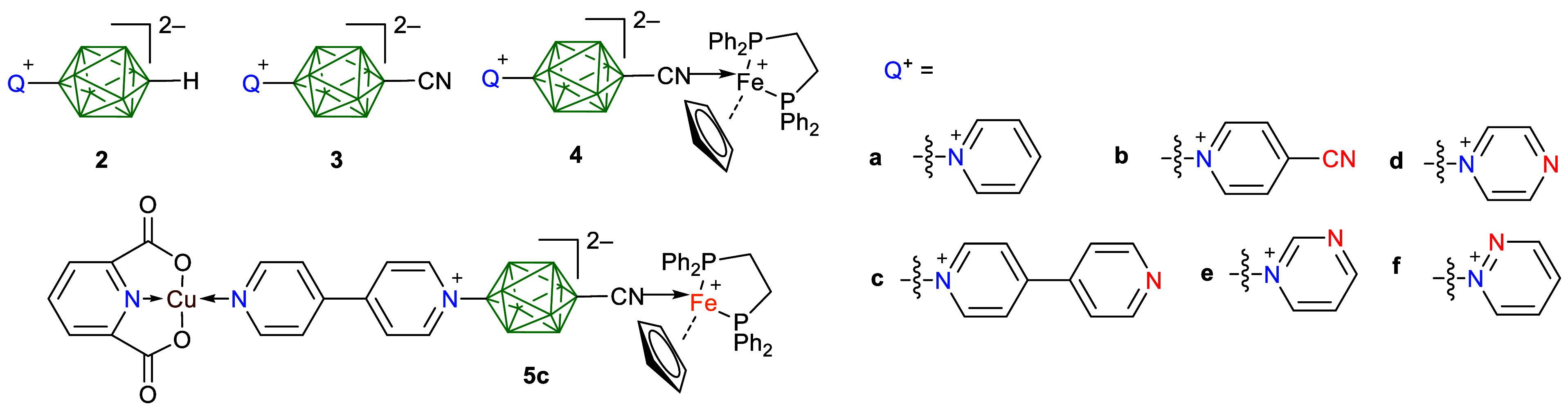
Structures of ligands **3**, complexes **4c**, **4d**, and **5c**, and reference derivatives **2a**, **2d**, and **2f**.

The azine at the B(10) position in **A** can be 4-cyanopyridine (**3b**), 4,4′-bipyridyl (**3c**) or pyrazine (**3d**), providing a rigid, linear ditopic ligand with variable length and coordination strength of the secondary interactions (Figure 2). We have also envisioned two angular ditopic ligands: derivatives of pyrimidine **3e** and pyridazine **3f**.

Herein we report the synthesis and characterization of nitrile derivatives **3** (Figure 2) as potential ditopic ligands for metal complexes and a step toward functional coordinational networks. The derivatives are characterized with spectroscopic (UV, IR, and NMR) and single-crystal XRD methods, while all experimental data are augmented with DFT results. For comparison purposes we also present selected derivatives **2** lacking the CN group. We demonstrate XRD structures for two complexes with Fe(η^5^-Cp)(dppe), **4c** and **4d**, formed from ligands **3c** and **3d**, respectively (Figure 2). Finally, we report a heterodinuclear metal complex **5c** derived from **3c**.

## Results and Discussion

### Synthesis

**Scheme 1:**
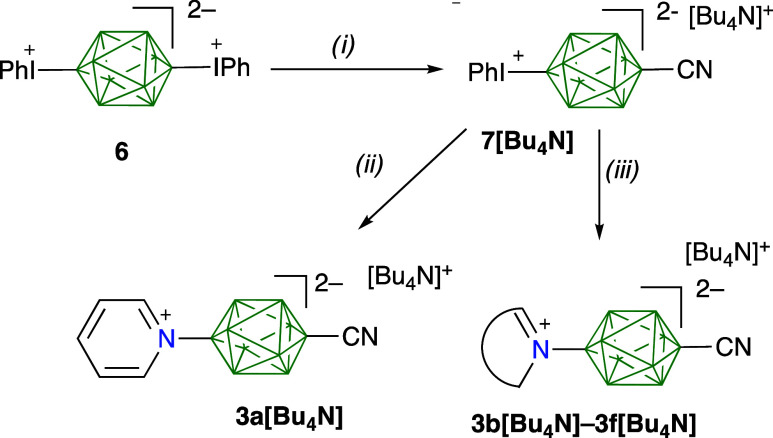
Synthesis of 3a[Bu_4_N]–3f[Bu_4_N] *Reagents and conditions*: (i) [Bu_4_N]^+^CN^–^, MeCN/THF, 60 °C, 12 h, 47–50%; (ii) pyridine, 80 °C, 16 h, 60%, ref (37); (iii) azine 5 equiv., MeCN, 80 °C, 16 h, 50–78%.

The nitriles **3** were obtained starting with bisiodonium zwitterion **6**,^43^ according to the previously developed general protocol (Scheme 1).^37^ Thus, bisiodonium **6** undergoes selective nucleophilic substitution with 1 eq of [Bu_4_N]^+^CN^–^ to give the monocyano derivative **7[Bu**_**4**_**N]** in 47–50% yield after column chromatography, according to a revised procedure (see the Supporting Information (SI)). The choice of the [Bu_4_N]^+^ counterion was dictated by the more facile reaction of **6** due to better solubility of the products, as compared to those containing the [Et_4_N]^+^ ion. The subsequent reaction of **7[Bu**_**4**_**N]** in neat pyridine gave **3a[Bu**_**4**_**N]** in 60% yield^37^ (Scheme 1).

**Scheme 2:**
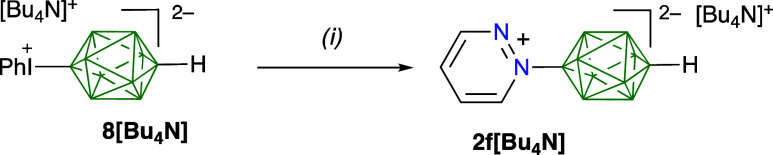
Synthesis of 2f[Bu_4_N] *Reagents and conditions*: (i) pyridazine, MeCN, 80 °C, 3 h, 61%.

To optimize conditions for the synthesis of other azine derivatives, reactivity of monoiodonium **8[Bu**_**4**_**N]** with pyridazine and formation of **2f[Bu**_**4**_**N]** in 50 mM MeCN solutions were briefly investigated at 80 °C (Scheme 2). The ratio of pyridazine to **8[Bu**_**4**_**N]** was varied (5:1, 10:1, and 20:1) and the progress of the reaction was monitored using ^11^B NMR spectroscopy. Results demonstrated that even for the lowest ratio of reagents 5:1, **8[Bu**_**4**_**N]** completely reacted within 3 h to form **2f[Bu**_**4**_**N]** in 61% yield. This method was used to prepare pyrazine derivative **2d[Bu**_**4**_**N]** in 25% yield after purification with column chromatography.

**Scheme 3:**
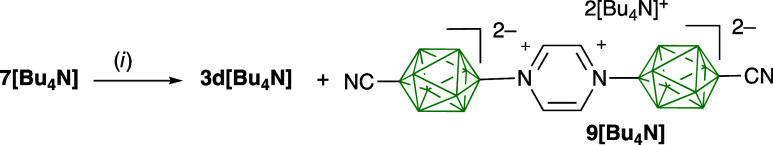
Synthesis of 3d[Bu_4_N] and 9[Bu_4_N] *Reagents and conditions*: (i) pyrazine, MeCN, 80 °C, 18 h.

Using conditions developed for the preparation of **2f[Bu**_**4**_**N]**, iodonium intermediate **7[Bu**_**4**_**N]** was reacted completely with pyridazine only after 18 h at 80 °C to give **3f[Bu**_**4**_**N]**. Therefore, the amount of solvent (MeCN) was reduced to concentration of 0.5 M, and **3b[Bu**_**4**_**N]**–**3f[Bu**_**4**_**N]** were obtained in 50–78% yields. Interestingly, the preparation of **3d[Bu**_**4**_**N]** gave an unexpected deep purple, doubly substituted side product **9[Bu**_**4**_**N]**, which was isolated in about 7% yield (Scheme 3). Attempts to increase the yield of **9[Bu**_**4**_**N]** using a 2:1 ratio of **7[Bu**_**4**_**N]** to pyrazine did not succeed, while treatment of iodonium **7[Bu**_**4**_**N]** with **3d[Bu**_**4**_**N]** gave only traces of **9[Bu**_**4**_**N]**. Such double zwitterions were not observed during the reaction of other azines.

**Scheme 4:**
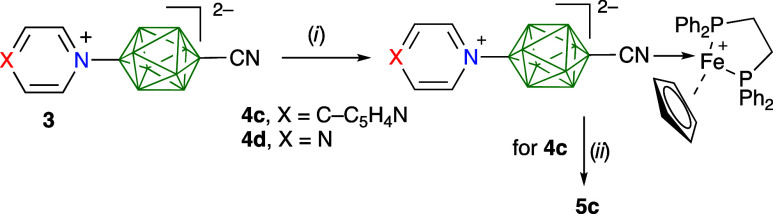
Preparation of Complexes **4c**, **4d**, and **5c** *Reagents and conditions*: (i) (η^5^-Cp)(dppe)FeCl, CH_2_Cl_2_, reflux, 16 h, 60–85%. (ii) **4c**, (pdc)Cu(aq)_3_, MeOH, quant.

Complexes **4** were prepared by refluxing appropriate nitrile **3** and (η^5^-Cp)(dppe)FeCl^44^ in CH_2_Cl_2_ (Scheme 4). The successful formation of the products was indicated by a color change of the reaction mixture from brown to dark red. The crude products were purified by passing through a short layer of SiO_2_, followed by recrystallization from AcOEt. The resulting products were obtained as solvates with 1/8 (**4c**) or 1/2 (**4d**) molecule of AcOEt in 60% and 85% yield, respectively. Both complexes are well soluble in CH_2_Cl_2_ and were conveniently purified by column chromatography. Treatment of complex **4c** with the trihydrate of copper(II) pyridine-2,6-dicarboxylate, (pdc)Cu(aq)_3_,^45,46^ in MeOH gave a nearly quantitative yield of heterodinuclear complex **5c** (Scheme 4), which quickly crystallized from the solution as long, thin brown needles. The complex was sparingly soluble in MeCN, and essentially insoluble in MeOH and CH_2_Cl_2_.

### Crystal and Molecular Structures

**Figure 3:**
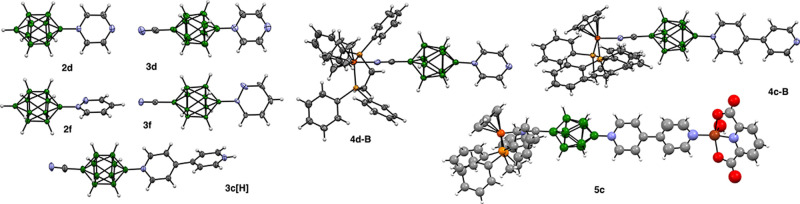
Atomic displacement ellipsoid representations of selected ligands **2** and **3**, and complexes **4**. The ellipsoids are drawn at the 50% probability level. Bottom: isotropically refined structure of **5c** for illustration purposes. Color codes: C-gray, B-green, O-red, N-blue, P-yellow, Fe-orange, Cu-brown.

**Figure 4:**
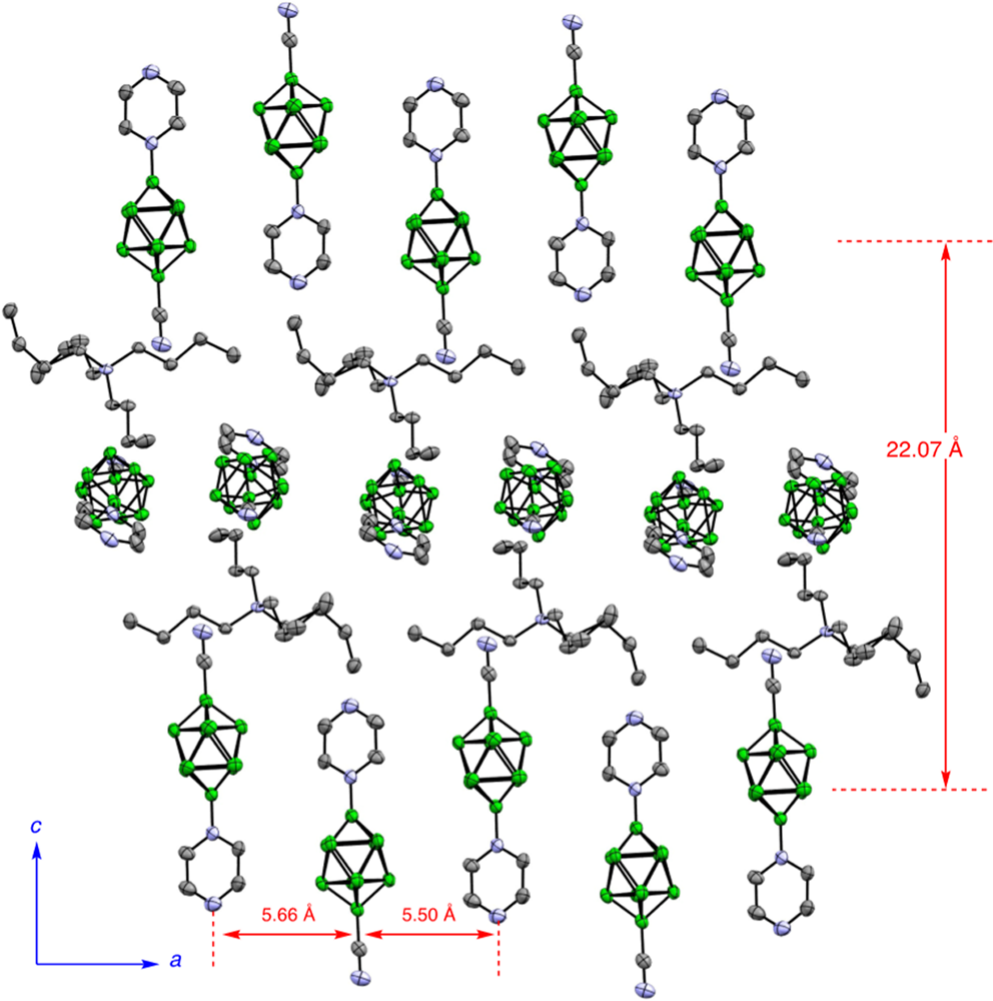
Partial packing diagram for **3d[Bu**_**4**_**N]** in the (011) plane. Hydrogen atoms are omitted for clarity. Atomic displacement ellipsoid diagram is drawn at 50% probability.

**Figure 5:**
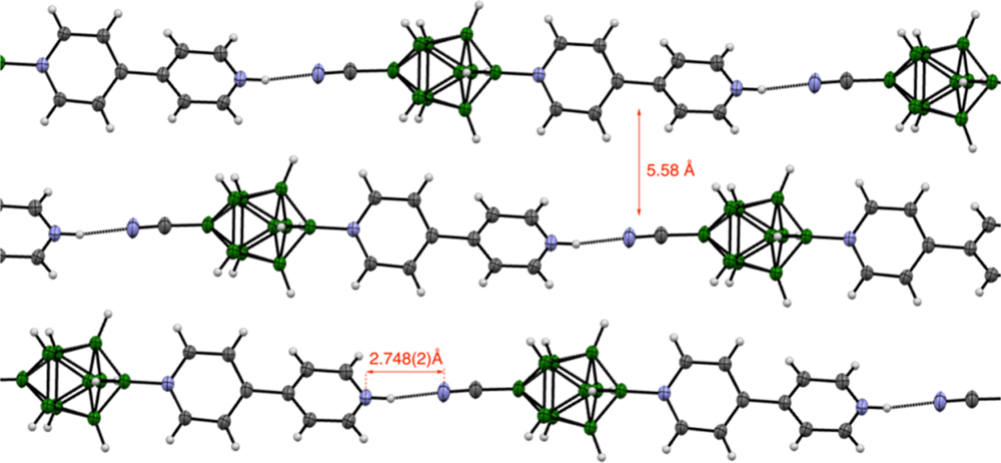
Partial packing diagram for **3c[H]** along the [100] direction. Atomic displacement ellipsoid diagram is drawn at 50% probability. The mean distances between the chain axes and the N···N separation are shown.

Yellow, monoclinic crystals suitable for single-crystal XRD analysis were obtained by slow cooling of EtOH solutions (**2d[Bu**_**4**_**N]**, *P*2_1_/n), or slow evaporation of CH_2_Cl_2_/EtOH solutions (**2f[Bu**_**4**_**N]***P*2_1_/n, **3d[Bu**_**4**_**N]***P*2_1_/n, and **3f[Bu**_**4**_**N]***P*2_1_/*c*). Similar evaporation of CH_2_Cl_2_/EtOH solutions of bipyridyl derivative **3c[Bu**_**4**_**N]** containing small amounts of acid gave light yellow triclinic crystals (*P̅*) of **3c[H]**. Red monoclinic (*P*2_1_/n) and triclinic (*P̅*) crystals of Fe complexes **4c** and **4d**, respectively, were grown by slow evaporation of AcOEt solutions. For **4d**, two triclinic polymorphs were found, both with the *P*-1 space group. Analysis was focused on polymorph B with smaller *R*1 value (0.0429 vs 0.0852). Selected bond lengths and angles of the investigated derivatives are collected in Table 1, while their molecular and crystal structures are shown in Figures 3–5. For comparison purposes, data for previously reported **2a[Et**_**4**_**N]** and **3a[Et**_**4**_**N]** are also included. Full details are provided in the SI.^46^

**Table 1:** Selected Interatomic Distances and Angles for Derivatives of [*closo*-B_10_H_10_]^2–^a

X =	[B_10_H_10_]^2–^b H	**2a[Et**_**4**_**N]**^c^^,^^d^ H	**3a[Et**_**4**_**N]**^e^ CN	**3c[H]** CN	**4c**^c^ CN[Fe]	**2d[Bu**_**4**_**N]** H	**3d[Bu**_**4**_**N]** CN	**4d**^c^ CN[Fe]	**2f[Bu**_**4**_**N]** H	**3f[Bu**_**4**_**N]** CN
B(1)–X			1.543(2)	1.544(2)	1.529		1.540(3)	1.531(3)		1.539(2)
B(10)–N		1.529(3)	1.524(2)	1.526(2)	1.525	1.525(2)	1.528(2)	1.524(3)	1.522(1)	1.525(2)
B(1)–B(2) avg	1.701(3)	1.699(6)	1.696(4)	1.692(4)	1.693(6)	1.701(3)	1.694(2)	1.692(4)	1.702(3)	1.697(2)
B(2)–B(3) avg	1.100	1.837(7)	1.853(2)	1.855(10)	1.857(11)	1.839(6)	1.850(6)	1.855(5)	1.845(8)	1.853(6)
B(1)···B(2–5)^e^	1.835(9)	1.095	1.077	1.069	1.070	1.097	1.075	1.079	1.092	1.079
B(2)–B(6) avg	1.813(6)	1.809(6)	1.811(6)	1.812(7)	1.812(9)	1.813(9)	1.811(7)	1.810(6)	1.811(6)	1.812(4)
B(6)–B(7) avg	1.835(9)	1.848(6)	1.856(1)	1.856(8)	1.854(3)	1.852(6)	1.852(7)	1.857(12)	1.855(9)	1.856(5)
B(10)···B(6–9)^f^	1.701(3)	1.056	1.052	1.056	1.054	1.054	1.053	1.050	1.055	1.057
B(10)–B(9) avg	1.100	1.680(5)	1.682(4)	1.685(7)	1.682(5)	1.681(2)	1.681(3)	1.681(3)	1.683(3)	1.685(2)
B(1)···B(10)	3.717(4)	3.660(4)	3.637(2)	3.632(2)	3.632	3.664(2)	3.638(3)	3.626	3.657(2)	3.644(2)
B–B(1)–X	130.3(12)	130.1(2)	129(2)	129(2)	129(3)	130(1)	129.4(5)	129(2)	130(1)	129(2)
B–B(10)–N	130.3(12)	129(2)	129(1)	129(4)	129(4)	129(4)	129(3)	129(2)	129(3)	129(1)
Azine/{B_10_}^g^^,^^f^		34.0/24.0	1.20	53.2		22.2	32.4	11, 23	5.2	23.1
C≡N			1.151(2)	1.147(2)	1.148		1.152(2)	1.154		1.153(2)
N→Fe					1.907			1.910(2)		

a For consistency the azine is substituted at the B(10) position in all derivatives. Except for unique interatomic distances, all parameters are average values and the esd refers to distribution of individual measurements.

b [*closo*-B_10_H_10_]^2–^[2,2′-bipyridinium], ref (49).

c Two molecules.

d Ref (50).

e Ref (37).

f The height of the tetragonal pyramid.

g Angle between mean plane of the azine ring and plane defined by B(1,2,4,10) atoms; 0° for an ideal staggered orientation.

All attempts at growing crystals of heterodinuclear complex **5c** suitable for XRD analysis by slow evaporation of MeOH, MeCN/MeOH or MeCN/EtOH solutions gave conglomerates of thin needless, which provided only partial data insufficient for anisotropic refinement. Therefore, results for **5c** are used for illustrative purposes only. The best crystal of **5c** exhibited the *I̅*4 space group.^46^

The asymmetric unit of crystal systems contains a single ion pair (**2[Bu**_**4**_**N]** and **3[Bu**_**4**_**N]**) or a single molecule (**3c[H]**), while two molecules are found for iron complexes **4c** and polymorph B of **4d**. The [Bu_4_N]^+^ cation was modeled without positional disorder and with one (**2d[Bu**_**4**_**N]**, **2f[Bu**_**4**_**N]**, and **3f[Bu**_**4**_**N]**) or two (**3d[Bu**_**4**_**N]**) butyl chains adopting a gauche conformation. In polymorph B of **4d**, one phenyl group in each molecule exhibits positional disorder modeled at about a 60:40 ratio. Crystallization solvent (presumably AcOEt) present in the structure of **4d**, as evident from additional electron density, was removed using the solvent mask function in the OLEX2.^47^

The intracage dimensions of the anions, such as B–B bond distances and angles, are typical for apical-substituted derivatives of {*closo*-B_10_} cluster (Table 1).^35,37^ Data indicate that substitution of anion **2** with the CN group has only a small effect on the {*closo*-B_10_} cage geometry. Thus, the B(1)–B bonds and the height of the B(1) square pyramid slightly contract (by 0.006 and 0.020 Å, respectively), while the B(2)–B(3) bonds slightly expand (0.013 Å) in **3** relative to **2**, all outside 3σ of the experimental error. This is consistent with structural effects of an electron withdrawing substituent at the apical position.^37,48^ The variation of the azinium substituent at the B(10) position has essentially no effect on the {*closo*-B_10_} cage geometry. Structural analysis indicates that the azinium substituent is a more strongly electron withdrawing substituent than the CN group. This is evident from the shorter B(10)–B bonds and the lower height of the B(10) square pyramid, when compared to the analogous dimensions at the B(1) apex (1.683(2) and 1.055(2) Å vs 1.695(2) and 1.075(4) Å, respectively).

The N–B(10) distance is essentially the same in the entire series (within the experimental error) with a mean value of 1.526(2) Å for all seven derivatives in Table 1. The B(1)–CN distance is 1.542(2) Å for all four derivatives and typical for other derivatives of the [*closo*-B_10_H_10_]^2–^ anion containing CN substituents.^37^ The azine rings typically adopt ideal staggered or pseudo staggered orientations with respect to the {*closo*-B_10_} cage, with the noticeable exception of **3c**, in which pyridine ring nearly eclipses the B(10)–B bonds. The overall length of the ditopic ligand **3c** is *d*_N···N_ = 14.687(2) Å and **3d***d*_N_..._N_ = 10.630(2) Å.

Complexation of the CN group with the Fe center has almost no effect on ligand’s geometry. The largest effect is the shortening of the B(1)–CN bond by 0.015 and 0.09 Å in ligands **4c** and **4d**, respectively. Other molecular dimensions, including the Fe–NC distances, are similar to those reported for the bimetallic complex of dinitrile **1** (1.910(2) and 1.902(2) Å).^36^ Although the structure of the bimetallic complex **5c** could not be refined anisotropically, the results provide a proof of concept and a glimpse into the potential of **3** as heteroditopic ligands. The Fe–NC and Cu–N bond lengths are 1.88(2) and 1.92(2) Å, respectively, which are typical for analogous complexes with nitriles (see **4c** and **4d**) and with pyridine derivatives.^51−53^ The metal centers in **5c** are 18.676(5) Å apart.

The crystals of **2d[Bu**_**4**_**N]**, **3d[Bu**_**4**_**N]**, and **3f[Bu**_**4**_**N]** are organized as fundamental antiparallel discrete dimers of two anions with C(2)–H···B(2,6) (**2d[Bu**_**4**_**N]** and **3d[Bu**_**4**_**N]**) or C(3)–H···B(2,6,7) (**3f[Bu**_**4**_**N]**) close contacts. The pyrazine derivatives **2d** and **3d** form undulated ribbons separated by [Bu_4_N]^+^ counterions (Figure 4). In the pyridazine derivative **2f[Bu**_**4**_**N]**, the anions form infinite zigzag chains using close C(3)–H···B(1,2,3) interactions.^46^ In contrast, the molecules of **3c[H]** are arranged in infinite linear chains running along the [100] direction through terminal N–H···NC interactions (*d*_N···N_ = 2.748(2) Å, Figure 5).

### Molecular Modeling

To provide a better understanding of the properties of the investigated compounds, their electronic structures and excitation energies were modeled using the TD-DFT method at the CAM-B3LYP/Def2TZVP//B3LYP/Def2TZVP level of theory in the MeCN dielectric medium. Geometry optimizations were performed with the B3LYP/Def2TZVP method for **2** and **3** in a weak dielectric medium (PhCl), since it was previously demonstrated that the inclusion of a dielectric medium is necessary to obtain accurate geometry of boron zwitterions.^35^

**Figure 6:**
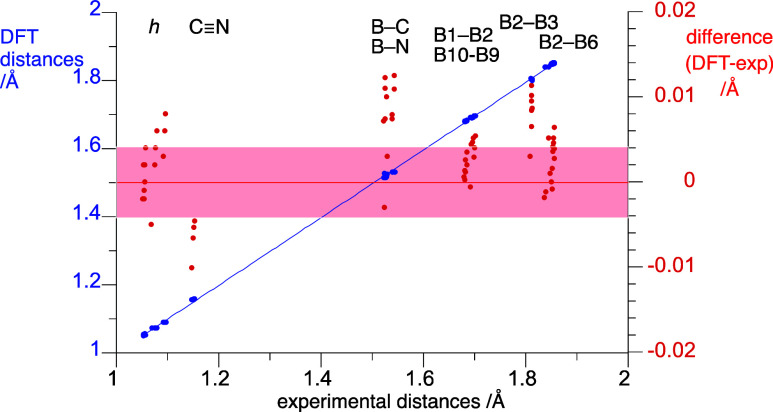
Left axis, blue data points: A comparison of the experimental and DFT derived molecular dimensions for anions **2** and **3**. The best fitting blue line: *d*_DFT_ = 0.995(2) × *d*_exp_+0.004(3), *r*^2^ = 0.9998. Right axis, red data points: differences between experimental and DFT values. The shadowed area corresponds to the typical experimental uncertainty of ±0.004 Å.

Results demonstrate that the DFT method well reproduces the experimental molecular dimensions listed in Table 1 and the correlation linear fitting function has a low error (Figure 6). Detailed analysis of the differences between the experimental and DFT interatomic distances revealed that the theoretical values are generally overestimated by up to 0.014 Å, while the C≡N bond lengths are underestimated with an average difference of 0.003(5) Å (Figure 6). Particularly overestimated are exocage distances B–CN and B–azine.

DFT calculations determined the equilibrium geometry of the bis-zwitterion **9** to have *D*_2h_ point group symmetry with shorter B–N and C–C distances (1.505 and 1.532 Å, respectively) than those found in the analogous monozwitterion **3d** (1.518 and 1.540 Å, respectively). This significant contraction results from the strongly electron withdrawing character of the doubly positive pyrazinium ring.

Normal mode analysis indicates that the C≡N stretching frequency in series **3** is in the narrow range of 2270 (**3a**) – 2274 (**3d**) cm^–1^, which corresponds to the experimentally determined values ranging from 2183 to 2189 cm^–1^. These stretching frequencies are significantly lower than those reported for benzonitrile (2228 cm^–1^) or *t*-BuCN (2235 cm^–1^). The indicated lower bond order of the C≡N group is consistent with efficient charge transfer from the {*closo*-B_10_} cluster.

The geometry optimization at the B3LYP/Def2SVP level of theory of model complexes **4**′, in which the dppe ligand was replaced with two PH_3_ groups, gave equilibrium structures with key interatomic distances that reasonably well reproduce experimental values for **4c** and **4d**. Thus, DFT determined Fe–N distances of about 1.92 Å, Fe–P 2.24 Å, and Fe–C 2.12 Å, which compare favorably to mean values measured experimentally: 1.908(3), 2.20(1), and 2.09(1) Å, respectively.

### Photophysical Properties

**Figure 7:**
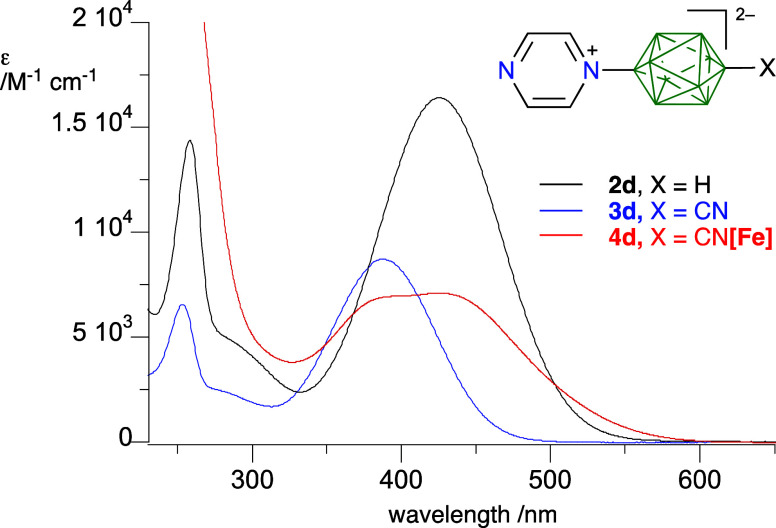
Electronic spectra of series **d** recorded in MeCN: **2d** (black), **3d** (blue), and **4d** (red).

Electronic absorption and emission spectra for zwitterions in series **2** and **3** and the two complexes **4c** and **4d** were recorded in MeCN solutions. Results shown in Table 2 and Figure 7 demonstrate that all ligands **2** and **3** exhibit a moderately strong (log ε ≈ 4.0) absorption band between 330 and 430 nm and weaker bands below 300 nm.

**Table 2:** Experimental and DFT-derived Electronic Transition Energies, Oscillator Strength Values and FMO Energies

	experimental^a^		DFT^b^		
	absorption^c^ λ_max_ (log ε)/nm	emission λ_max_ (*S* shift)/nm (eV)	π→ π* (*f*)^c^/eV	*E*_HOMO_/eV	*E*_LUMO_/eV
**2a**^d^	365 (3.85)^d^	568(1.21)	313(0.29)	–7.038	–0.871
**2b**^d^	452 (3.98)^d^	e	390 (0.42)	–7.092	–1.911
**2d**	426.0 (4.22)	e	365(0.32)	–7.144	–1.579
**2f**	422.5 (4.03)	e	364(0.34)	–7.065	–1.469
**3a**^d^	339.0 (3.85)^d^	538.5(1.36)	296(0.33)	–7.345	–0.948
**3b**	409.5 (3.96)	e	362(0.45)	–7.408	–1.971
**3c**	378.5 (4.20)	581(1.14)	324(0.58)	–7.338	–1.529
**3d**	387.5 (3.94)	e	341(0.34)	–7.458	–1.653
**3e**	360.5 (3.78)	554(1.21)	313(0.32)	–7.417	–1.291
**3f**	385.0 (4.02)	e	341(0.37)	–7.377	–1.539
**4c**	380.2 (4.03)	e	f	f	f
**4d**	395sh (3.84)	e	f	f	f
	425.4 (3.85)				
**9**	554.0 (4.18)	e	481(1.01)	–7.299	–2.677

a Recorded in MeCN.

b Obtained with the CAM-B3LYP/Def2TZVP//B3LYP/Def2TZVP method in MeCN dielectric medium.

c Energy corresponding to the charge transfer (CT) excitation.

d Ref (37).

e No fluorescence.

f Not calculated. DFT with a smaller basis set.

The lowest energy and also the highest intensity of this band is observed for the double zwitterion **9** with λ_max_ of 554 nm and *log* ε = 4.18. The low energy band is ascribed to the intramolecular CT excitation, and its intensity is an indicator of the ability of the {*closo*-B_10_} to couple strongly with π symmetry azines. Analysis of three pairs of derivatives **2** and **3** indicates that substitution of the CN group at the apical position in **2** results in a hypsochromic shift of the CT band by about 0.28 eV in the corresponding derivatives **3**. This effect is consistent with stabilization of the HOMO and widening the HOMO–LUMO gap. Further analysis of series **3** indicates that the energy of the charge transfer band follows the order **3a** (pyridinium) < **3e** (pyrimidinium) < **3f** (pyridazinium) < **3d** (pyrazinium), which correlates well with the order of experimentally estimated electron affinities, *E*_aff_, of free azines^54^ (*E*_CT_ = 3.38(3) × *E*_aff_ −0.47(7) eV; *r*^2^ = 0.96).

The formation of the Fe complexes **4c** and **4d** affects the position of the CT bands in ligands **3c** and **3d** marginally. In both complexes, however, an additional, low energy absorption band related to the Fe center appears and overlaps with the CT signal. This band is of low intensity and merged with the CT band in **4c**, while in **4d** it is distinct with a maximum at 425.4 nm (Figure 7). Low solubility hampered detailed analysis of **5c**.

Excitation at the peak of the low energy absorption band results in fluorescence in pyridine derivatives **2a** and **3a**, and also in dipyridyl **3c** and pyrimidine **3e** (Table 2). The Stokes shift in these compounds is characteristically large and in the range of 1.14 eV for **3c** to 1.36 eV for **3a**. A comparative qualitative analysis indicates that the two fluorescent azine derivatives **3c** and **3e** have low fluorescence intensities relative to **2a**.

**Figure 8:**
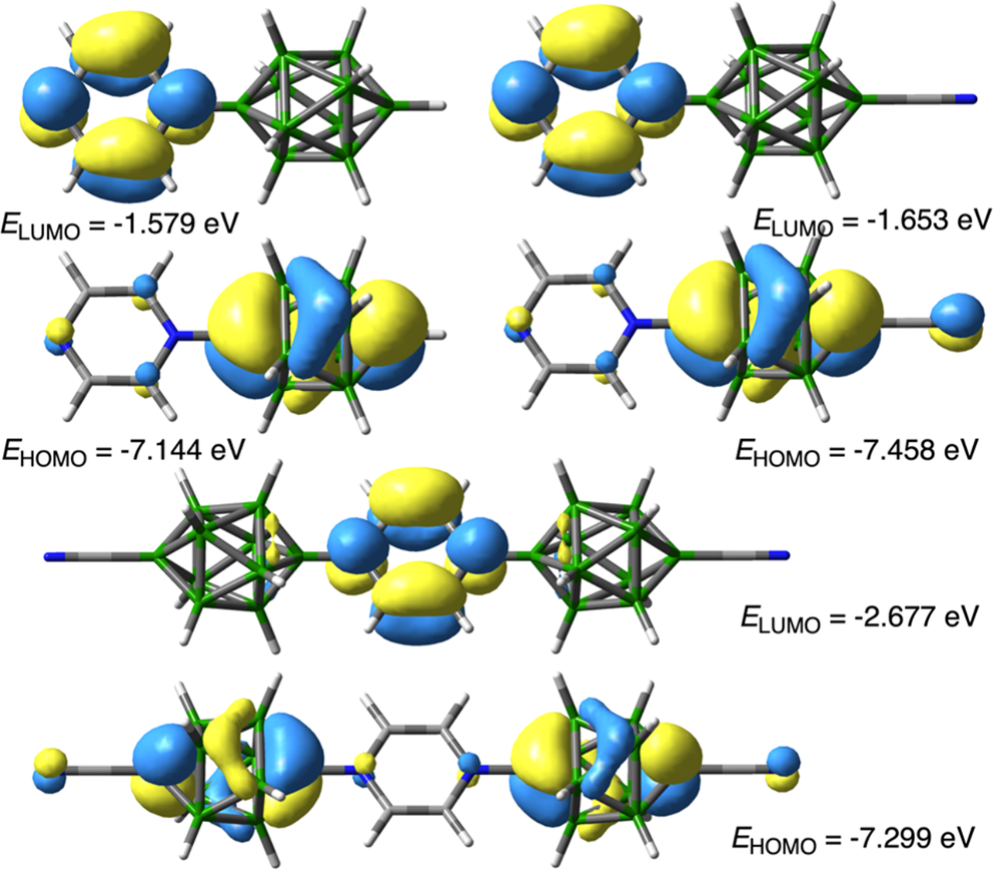
FMO contours and energies for **2d** (upper left), **3d** (upper right) and **9** (lower) anions obtained using the CAM-B3LYP/Def2TZVP//B3LYP/Def2TZVP method in MeCN dielectric medium (MO *isovalue* = 0.04).

**Figure 9:**
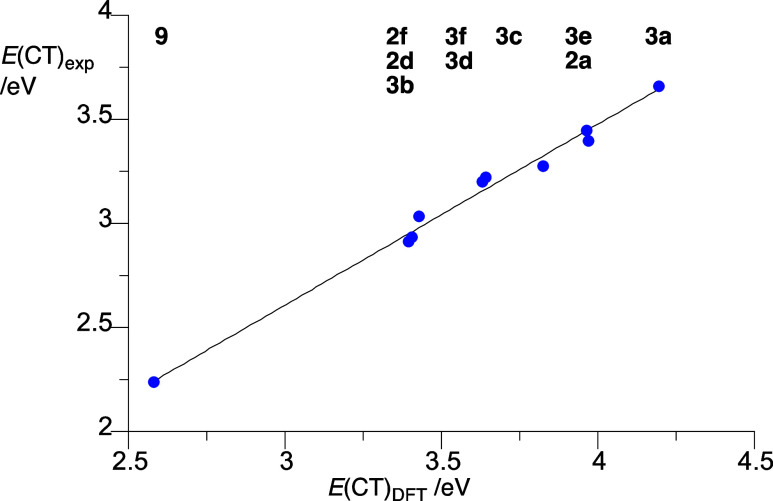
Correlation of experimental and TD-DFT derived energies of the CT band in series **2** and **3** in MeCN. Best line fit: *E*(CT)_exp_ = *E*(CT)_DFT_ × 0.869(4), *r*^2^ = 0.989.

TD-DFT calculations confirmed that the low energy absorption band in ligands **2** and **3** is a π–π* excitation with intramolecular charge transfer (CT) character. Thus, results indicate that the lowest energy excitation (S_0_ → S_1_) is predominately (∼50%) due to the π–π* transition between the HOMO, localized mainly on the {*closo*-B_10_} cluster, to the LUMO, solely associated with the azinium substituent as shown in Figure 8 for selected anions. The energies of the CT bands calculated with the TD-CAM-B3LYP/Def2TZVP // B3LYP/Def2TZVP method in the MeCN dielectric medium correlate well with the experimentally determined values (Figure 9).

**Figure 10:**
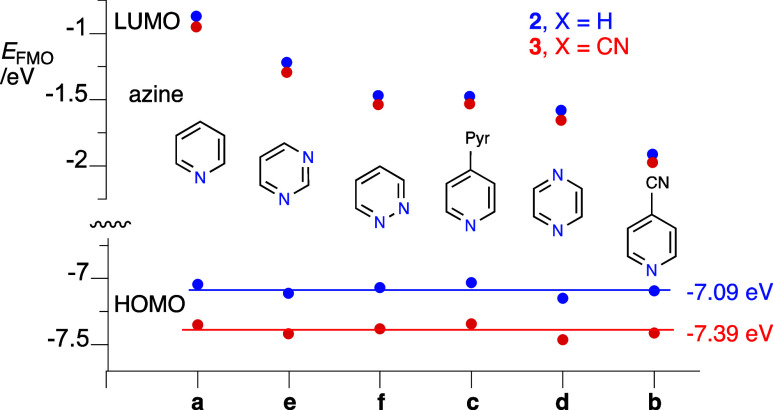
A comparison of energies of the FMOs for ligands in series **2** and **3** obtained with the CAM-B3LYP/Def2TZVP//B3LYP/Def2TZVP method in the MeCN dielectric medium.

Further analysis of the DFT results revealed that the variation in the CT energy in each series **2** and **3** is almost exclusively due to the nature of the azine and associated level of the LUMO. Figure 10 shows that incorporation of the second N atom into the azine ring lowers the LUMO, with the strongest effect for the pyrazine derivative (by 0.7 eV), while the HOMO is also lowered by about 0.1 eV. The strongest effect is observed for 4-cyanopyridine, which lowers the LUMO by about 1.0 eV, while the HOMO is changed by only 0.05 eV. Substitution of the CN group at the {*closo*-B_10_} cluster shifts the HOMO energy down by an average of 0.3 eV, while the LUMO is lowered by 0.07 eV.

### E-chem Analysis of Fe Complexes

**Figure 11:**
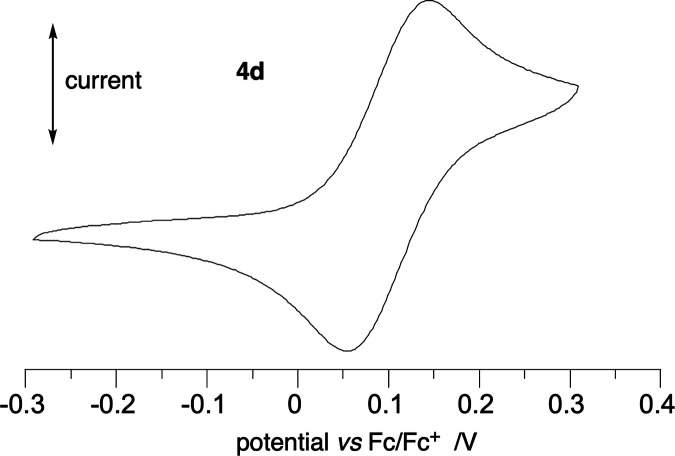
Cyclic voltammogram of complex **4d**.

Cyclic voltammetry in CH_2_Cl_2_ demonstrated a single quasireversible oxidation process for both complexes **4c** and **4d** with halfwave potential, *E*_1/2_, of 0.080 and 0.101 V *vs* the Fc/Fc^+^ couple, respectively (Figure 11 and Table 3). In comparison, these potentials are slightly higher than those measured for the analogous diiron complex of dinitrile **1**, and all three follow the trend in the DFT calculated level of the HOMO for the model complexes (Table 3).

**Table 3:** Oxidation Potentials for Complexes 4^a^

complex	*E*_1/2_/V	*E*_HOMO_^b^/eV
**1[Fe]**	0.056^c^	–5.52
**4c**	0.080	–5.66
**4d**	0.101	–5.69

a 0.5 mM in CH_2_Cl_2_ [*n*-Bu_4_N]^+^[PF_6_]^−^ (100 mM), at *ca*. 22 °C, 50 mV s^–1^, glassy carbon working electrode. Referenced to the Fc/Fc^+^ couple.

b Obtained with the B3LYP/Def2SVP method in CH_2_Cl_2_ dielectric medium.

c Ref (36).

## Conclusions

A new, potentially broad class of functional, rigid, heteroditopic ligands with intense intramolecular CT bands in the range of 340–410 nm (MeCN) and delocalized net negative charge has been demonstrated. Thus, six ligands were obtained from the [*closo*-B_10_H_10_]^2–^ anion in three steps and 25–30% overall yield, demonstrating a general synthetic method. Analysis of structural and spectroscopic data for the ligands, and their analogues lacking the CN group, revealed a relatively small effect of the CN group on the {*closo*-B_10_} cluster geometry and an hypsochromic shift of 0.28 eV. On the other hand, the azine has a significant effect on all electronic absorption spectra through modification of the LUMO. The B3LYP/Def2TZVP computational method well reproduced the experimental structural and electronic absorption data, which indicates that it can be used to design new ligands with tailored properties.

The binding ability of the ligands’ electron rich CN group was exemplified by the formation of two iron complexes, in which the positive charge of the FeCp(dppe) center was compensated with the negative charge on ligands **3c** and **3d**. The complex with the bipyridyl substituent (**4c**) was used to demonstrate the ditopic properties of ligand **3c** by forming a linear, heterodinuclear complex containing the FeCp(dppe) and Cu(pdc) centers spaced by 18.68 Å (**5c**).

These results demonstrate convenient access to these and related ligands, such as linear ligand **3b** (containing the secondary CN group) or pyrimidine **3e** (angular ligand), as functional structural elements for metal ion frameworks and other materials.

## Computational Details

Quantum-mechanical calculations were carried out using Gaussian 09 suite of programs.^55^ Geometry optimizations were performed with the B3LYP functional and Ahlrichs Def2TZVP (compounds **2**, **3**, and **9**) or Def2SVP (complexes **4**) basis set,^56,57^ using tight convergence limits and appropriate symmetry constraints. All geometry optimizations were performed in PhCl dielectric medium implemented with the PCM model^58^ using the SCRF(solvent = C6H5Cl) keyword. The nature of stationary points was confirmed with vibrational frequency calculations. Zero-point energy (ZPE) corrections were scaled by 0.9806.

Electronic excitation energies for series **2**, **3**, and **9** in a MeCN dielectric medium were obtained at the CAM-B3LYP/Def2TZVP // B3LYP/Def2TZVP level of theory using the time-dependent DFT method^59^ supplied in the Gaussian package, and TD(NStates = 20) and SCF = tight keywords. The equilibrium geometry for each compound was obtained in a PhCl dielectric medium (*vide supra*). The solvation model was implemented with the PCM model^58^ using the SCRF(solvent = CH3CN) keyword.

## Experimental section

### General

Reactions were carried out under Ar and subsequent manipulations were conducted in air. Literature procedures were used to obtain [*closo*-B_10_H_10_]^2–^ 2[Et_3_NH]^+^,^60^**7[Bu**_**4**_**N]**,^37^ and **8[Bu**_**4**_**N]**.^43^ NMR spectra were obtained at 500 MHz (^1^H), 126 MHz (^13^C), and 160 MHz (^11^B) in acetone-*d*_6_ unless otherwise indicated. Chemical shifts were referenced to the solvent (^1^H and ^13^C: 2.05 and 29.84 ppm for acetone-*d*_6_) or an external sample of neat BF_3_**·**Et_2_O (^11^B, δ = 0.0 ppm). ^11^B NMR chemical shifts are reported from {^1^H} decoupled spectra. IR spectra were recorded for neat samples using an ATR attachment. HR mass spectrometry was conducted with the TOF-MS ESI method in the negative mode at the Mass Spectrometry and Proteomics Facility at the University of Notre Dame. UV–vis spectra were recorded in spectroscopic grade MeCN at concentrations in a range of 1–10 × 10^–5^ M. Details for measurement of electronic absorption spectra and XRD are given in the SI.

### General Procedure for the Synthesis of Series 2[Bu_4_N] and 3[Bu_4_N]

The mixture of [*closo*-B_10_H_9_-1-IPh]^−^[Bu_4_N]^+^ (ref (43), 8**[Bu**_**4**_**N]**, 56 mg, 0.10 mmol) or [*closo*-B_10_H_8_-1-CN-10-IPh]^−^[Bu_4_N]^+^ (ref,^37^**7[Bu**_**4**_**N]**, 59 mg, 0.10 mmol) and azine (5 equiv) in MeCN (0.2 mL) was stirred at 80 °C for 3 h (for series **2**) or overnight (for series **3**) and volatiles were removed under vacuum. The residue was washed with hexanes, 10% HCl and with H_2_O, and then purified using gradient flash column chromatography (SiO_2_) starting with 9:1 CH_2_Cl_2_/MeCN, followed by recrystallization from EtOH or CH_2_Cl_2_/EtOH. When **3d[Bu**_**4**_**N]** was prepared from **7[Bu**_**4**_**N]** and pyrazine, and purified as described above, a small amount of **9[Bu**_**4**_**N]** was obtained.

#### [*closo*-B_10_H_9_-1-Pyrazinium]^−^ [Bu_4_N]^+^ (**2d[Bu**_**4**_**N]**)

Product **2d[Bu**_**4**_**N]** was obtained in 25% yield (32.3 mg) using **8[Bu**_**4**_**N]** (163 mg, 0.29 mmol) and pyrazine (116 mg, 1.45 mmol) as an orange powder recrystallized from EtOH: *R*_f_ = 0.53 (5:1 CH_2_Cl_2_/MeCN); mp 133–134 °C (EtOH); ^1^H NMR (500 MHz, acetone-*d*_6_) δ 0.05–1.65 (br m, 8H), 0.98 (t, *J* = 7.4 Hz, 12H), 1.44 (sext, *J* = 7.3 Hz, 8H), 1.82 (pseudo quin, *J* = 8.0 Hz, 8H), 3.44 (pseudo t, *J* = 8.6 Hz, 8H), 4.16 (q, *J* = 146 Hz, 1H), 9.12 (dd, *J*_1_ = 3.1 Hz, *J*_2_ = 1.0 Hz, 2H), 9.52 (dd, *J*_1_ = 3.0 Hz, *J*_2_ = 0.9 Hz, 2H); ^13^C{^1^H} NMR (126 MHz, acetone-*d*_6_) δ 13.9, 20.4, 24.5, 59.5, 141.7, 148.6; ^11^B NMR (160 MHz, acetone-*d*_6_) δ −24.0 (d, *J* = 133 Hz, 4B), −19.2 (d, *J* = 132 Hz, 4B), 11.5 (d, *J* = 148 Hz, 1B), 15.6 (s, 1B); IR (ATR) *v* 3101, 2966, 2888, 2468 (BH), 1604, 1420, 1171, 820, 727 cm^–1^; UV (CH_3_CN) λ_max_ (log ε) 258.5 (4.16), 426.0 (4.22) nm; HRMS (ESI, −) *m*/*z* calcd. for C_4_H_13_B_10_N_2_: 199.2015, found: 199.2021. Anal. Calcd for C_20_H_49_B_10_N_3_: C, 54.63; H, 11.23; N, 9.56. Found: C, 54.34; H, 11.45; N, 9.63.

#### [*closo*-B_10_H_9_-1-Pyridazinium]^−^[Bu_4_N]^+^ (**2f[Bu**_**4**_**N]**)

Product **2f[Bu**_**4**_**N]** was obtained in 61% yield (27.0 mg) using **8[Bu**_**4**_**N]** (56 mg, 0.10 mmol) and pyridazine (40 mg, 36 μL, 0.50 mmol) as an orange powder recrystallized from EtOH/CH_2_Cl_2_: *R*_f_ = 0.27 (9:1 CH_2_Cl_2_/MeCN); mp 148–149 °C (EtOH/CH_2_Cl_2_); ^1^H NMR (500 MHz, acetone-*d*_6_) δ 0.06–1.60 (br m, 8H), 0.97 (t, *J* = 7.4 Hz, 12H), 1.43 (sext, *J* = 7.4 Hz, 8H), 1.80 (pseudo quin, *J* = 8.0 Hz, 8H), 3.42 (pseudo t, *J* = 8.6 Hz, 8H), 4.08 (q, *J* = 146 Hz, 1H), 8.23 (ddd, *J*_1_ = 8.1 Hz, *J*_2_ = 5.0 Hz, *J*_3_ = 1.2 Hz, 1H), 8.30 (ddd, *J*_1_ = 7.9 Hz, *J*_2_ = 5.9 Hz, *J*_3_ = 2.0 Hz, 1H), 9.33 (d, *J* = 4.9 Hz, 1H), 10.29 (d, *J* = 5.8 Hz, 1H); ^13^C{^1^H} NMR (126 MHz, acetone-*d*_6_) δ 13.9, 20.4, 24.5, 59.5 (t, *J* = 2.8 Hz), 131.4, 132.5, 151.2, 153.1; ^11^B NMR (160 MHz, acetone-*d*_6_) δ −25.0 (d, *J* = 131 Hz, 4B), −20.2 (d, *J* = 132 Hz, 4B), 9.5 (d, *J* = 146 Hz 1B), 16.1 (s, 1B); IR (ATR) *v* 3097, 2933, 2960, 2874, 2449 (BH), 1566, 1446, 1416, 1293, 880 cm^–1^; UV (CH_3_CN) λ_max_ (log ε) 422.5 (4.03) nm; HRMS (ESI, −) *m*/*z* calcd. for C_4_H_13_B_10_N_2_: 199.2015, found: 199.2021. Anal. Calcd for C_20_H_49_B_10_N_3_: C, 54.63; H, 11.23; N, 9.56. Found: C, 54.62; H, 11.16; N, 9.37.

#### [*closo*-B_10_H_8_-1-CN-10-(4-Cyanopyridinium]^−^[Bu_4_N]^+^ (**3b[Bu**_**4**_**N**])

Product **3b[Bu**_**4**_**N]** was obtained in 55% yield (40 mg) using **7[Bu**_**4**_**N]** (92 mg, 0.16 mmol) and 4-cyanopyridine (83 mg, 0.80 mmol) as orange crystals recrystallized from EtOH: *R*_f_ = 0.70 (5:1 CH_2_Cl_2_/MeCN); mp 169–170 °C (EtOH); ^1^H NMR (500 MHz, acetone-*d*_6_) δ 0.35–1.67 (br m, 8H), 0.98 (t, *J* = 7.4 Hz, 12H), 1.44 (sext, *J* = 7.4 Hz, 8H), 1.83 (pseudo quint, *J* = 8.0 Hz, 8H), 3.44 (pseudo t, *J* = 8.6 Hz, 8H), 8.37 (dd, *J*_1_ = 5.4 Hz, *J*_2_ = 1.2 Hz, 2H), 9.74 (d, *J* = 6.7 Hz, 2H); ^13^C{^1^H} NMR (126 MHz, acetone-*d*_6_) δ 13.9, 20.4, 24.5, 59.5, 116.3, 125.2, 129.5, 149.6. The signal corresponding to the CN carbon was not observed; ^11^B NMR (160 MHz, acetone-*d*_6_) δ −22.5 (d, *J* = 138 Hz, 4B), −21.0 (d, *J* = 133 Hz, 4B), −1.0 (s, 1B), 19.8 (s, 1B); IR (ATR) *v* 3109, 2962, 2876, 2485 (BH), 2183 (CN), 1494, 1428, 1232, 837, 735 cm^–1^; UV (CH_3_CN) λ_max_ (log ε) 259.5 (3.73), 409.5 (3.96) nm; HRMS (ESI, −) *m*/*z* calcd. for C_7_H_12_B_10_N_3_: 248.1967, found: 248.1987. Anal. Calcd for C_21_H_48_B_10_N_4_: C, 56.52; H, 9.90; N, 11.46. Found: C, 56.27; H, 9.98; N, 11.82.

#### [*closo*-B_10_H_8_-1-CN-10-(4,4′-Bipyridinium)]^−^[Bu_4_N]^+^ (**3c[Bu**_**4**_**N**])

Product **3c[Bu**_**4**_**N]** was obtained in 50% yield (22 mg) using **7[Bu**_**4**_**N]** (48 mg, 0.081 mmol) and 4,4′-bipyridine (64 mg, 0.41 mmol) as orange crystals recrystallized from Et_2_O/CH_2_Cl_2_. *R*_f_ = 0.35 (5:1 CH_2_Cl_2_/MeCN); mp 166–167 °C (Et_2_O/CH_2_Cl_2_); ^1^H NMR (500 MHz, acetone-*d*_6_) δ 0.25–1.70 (br m, 8H), 0.98 (t, *J* = 7.4 Hz, 12H), 1.44 (sext, *J* = 7.4 Hz, 8H), 1.83 (pseudo quint, *J* = 8.0 Hz, 8H), 3.45 (pseudo t, *J* = 8.6 Hz, 8H), 8.01 (dd, *J*_1_ = 4.4 Hz, *J*_2_ = 1.7 Hz, 2H), 8.34–8.37 (m, 2H), 8.87 (dd, *J*_1_ = 4.4 Hz, *J*_2_ = 1.7 Hz, 2H), 9.60 (dd, *J*_1_ = 5.6 Hz, *J*_2_ = 1.3 Hz, 2H); ^13^C{^1^H} NMR (126 MHz, acetone-*d*_6_) δ 13.9, 20.4, 24.5, 59.5, 122.5, 124.6, 143.7, 149.1, 151.1, 152.0. The signal corresponding to the CN carbon was not observed; ^11^B NMR (160 MHz, acetone-*d*_6_) δ −24.3 (d, *J* = 168 Hz, 4B), −23.3 (d, *J* = 165 Hz, 4B), −4.9 (s, 1B), 19.2 (s, 1B); IR (ATR) *v* 3109, 3044, 2962, 2876, 2485 (BH), 2183 (CN), 1632, 1595, 1485, 1212, 825, 633 cm^–1^; UV (CH_3_CN) λ_max_ (log ε) 250.5 (4.38), 378.5 (4.20) nm; HRMS (ESI, −) *m*/*z* calcd. for C_11_H_16_B_10_N_3_: 300.2280. Found: 300.2290. Anal. Calcd for C_27_H_52_B_10_N_4_: C, 59.96; H, 9.69; N, 10.36. Found: C, 59.81; H, 9.72; N, 10.36.

#### [*closo*-B_10_H_8_-1-CN-10-Pyrazinium]^−^[Bu_4_N]^+^ (**3d[Bu**_**4**_**N**])

Product **3d[Bu**_**4**_**N]** was obtained in 53% yield (24.6 mg) using **7[Bu**_**4**_**N]** (59 mg, 0.10 mmol) and pyrazine (40 mg, 0.50 mmol) as yellow crystals recrystallized from EtOH: *R*_f_ = 0.60 (5:1 CH_2_Cl_2_/MeCN); mp 152–153 °C (EtOH); ^1^H NMR (500 MHz, acetone-*d*_6_) δ 0.35–1.65 (br m, 8H), 0.98 (t, *J* = 7.4 Hz, 12H), 1.44 (sext, *J* = 7.4 Hz, 8H), 1.83 (pseudo quint, *J* = 8.0 Hz, 8H), 3.45 (pseudo t, *J* = 8.6 Hz, 8H), 9.25 (dd, *J*_1_ = 3.0 Hz, *J*_2_ = 1.6 Hz, 2H), 9.50 (dd, *J*_1_ = 3.0 Hz, *J*_2_ = 1.3 Hz, 2H); ^13^C{^1^H} NMR (126 MHz, acetone-*d*_6_) δ 13.9, 20.4, 24.5, 59.5, 141.4, 149.3. The signal corresponding to the CN carbon was not observed; ^11^B NMR (160 MHz, acetone-*d*_6_) δ −22.8 (d, *J* = 143 Hz, 4B), −21.1 (d, *J* = 138 Hz, 4B), −0.7 (s, 1B), 18.5 (s, 1B); IR (ATR) *v* 3609, 3410, 3104, 2968, 2872, 2485 (BH), 2186 (CN), 1607, 1482, 1426, 1168, 829, 633 cm^–1^; UV (CH_3_CN) λ_max_ (log ε) 253.5 (3.82), 387.5 (3.94) nm; HRMS (ESI, −) *m*/*z* calcd. for C_5_H_12_B_10_N_3_: 224.1967. Found: 224.1969. Anal. Calcd for C_21_H_48_B_10_N_4_: C, 54.27; H, 10.41; N, 12.06. Found: C, 54.16; H, 10.46; N, 12.05.

#### [*closo*-B_10_H_8_-1-CN-10-Pyrimidinium]^−^[Bu_4_N]^+^ (**3e[Bu**_**4**_**N**])

Product **3e[Bu**_**4**_**N]** was obtained in 57% yield (26.5 mg) using **7[Bu**_**4**_**N]** (59 mg, 0.10 mmol) and pyrimidine (40 mg, 0.50 mmol) as yellow crystals recrystallized from EtOH: *R*_f_ = 0.58 (5:1 CH_2_Cl_2_/MeCN); mp 122–123 °C (EtOH); ^1^H NMR (500 MHz, acetone-*d*_6_) δ 0.35–1.65 (br m, 8H), 0.97 (t, *J* = 7.3 Hz, 12H), 1.43 (sext, *J* = 7.5 Hz, 8H), 1.81 (pseudo quint, *J* = 8.0 Hz, 8H), 3.43 (pseudo t, *J* = 8.5 Hz, 8H), 8.16 (dd, *J*_1_ = 6.1 Hz, *J*_2_ = 5.1 Hz, 1H), 9.37 (dd, *J*_1_ = 5.1 Hz, *J_2_* = 1.2 Hz, 1H), 9.78 (d, *J* = 6.1 Hz, 1H), 10.14 (s, 1H); ^13^C{^1^H} NMR (126 MHz, acetone-*d*_6_) δ 13.9, 20.4, 24.5, 59.4, 123.5, 155.3, 156.6, 162.2. The signal corresponding to the CN carbon was not observed; ^11^B NMR (160 MHz, acetone-*d*_6_) δ −23.5 (d, *J* = 159 Hz, 4B), −22.4 (d, *J* = 154 Hz, 4B), −2.9 (s, 1B), 17.1 (s, 1B); IR (ATR) *v* 3109, 3082, 2970, 2873, 2491 (BH), 2188 (CN), 1608, 1477, 1413, 1174, 893, 698 cm^–1^; UV (CH_3_CN) λ_max_ (log ε) 232.0 (3.82), 360.5 (3.78) nm; HRMS (ESI, −) *m*/*z* calcd. for C_5_H_12_B_10_N_3_: 224.1967; found: 224.1969. Anal. Calcd for C_21_H_48_B_10_N_4_: C, 54.27; H, 10.41; N, 12.06. Found: C, 54.00; H, 10.66; N, 12.04.

#### [*closo*-B_10_H_8_-1-CN-10-Pyridazinium]^−^[Bu_4_N]^+^ (**3f[Bu**_**4**_**N**])

Product **3f[Bu**_**4**_**N]** was obtained in 78% yield (36.6 mg) using **7[Bu**_**4**_**N]** (59 mg, 0.10 mmol) and pyridazine (40 mg, 36 μL, 0.50 mmol) as yellow crystals recrystallized from EtOH: *R*_f_ = 0.55 (5:1 CH_2_Cl_2_/MeCN); mp 173–174 °C (EtOH); ^1^H NMR (500 MHz, acetone-*d*_6_) δ 0.35–1.65 (br m, 8H), 0.98 (t, *J* = 7.3 Hz, 12H), 1.44 (sext, *J* = 7.4 Hz, 8H), 1.82 (pseudo quint, *J* = 8.1 Hz, 8H), 3.44 (pseudo t, *J* = 8.6 Hz, 8H), 8.35 (ddd, *J*_1_ = 8.3 Hz, *J*_2_ = 5.1 Hz, *J*_3_ = 1.7 Hz, 1H), 8.42 (ddd, *J*_1_ = 8.0 Hz, *J*_2_ = 5.7 Hz, *J*_3_ = 2.0 Hz, 1H), 9.43 (d, *J* = 4.9 Hz, 1H), 10.28 (d, *J* = 5.8 Hz, 1H); ^13^C{^1^H} NMR (126 MHz, acetone-*d*_6_) δ 13.9, 20.4, 24.5, 59.5, 132.7, 133.1, 151.7, 153.5. The signal corresponding to the CN carbon was not observed; ^11^B NMR (160 MHz, acetone-*d*_6_) δ −23.2 (d, *J* = 139 Hz, 4B), −21.5 (d, *J* = 138 Hz, 4B), −2.1 (s, 1B), 19.5 (s, 1B); IR (ATR) *v* 3098, 3075, 2963, 2873, 2499 (BH), 2188 (CN), 1586, 1469, 1425, 1166, 889 cm^–1^; UV (CH_3_CN) λ_max_ (log ε) 227.0 (3.74), 385.0 (4.02) nm; HRMS (ESI, −) *m*/*z* calcd. for C_5_H_12_B_10_N_3_: 224.1967; found: 224.1972. Anal. Calcd for C_21_H_48_B_10_N_4_: C, 54.27; H, 10.41; N, 12.06. Found: C, 54.14; H, 10.44; N, 12.11.

#### [{(η^5^-Cp)(dppe)Fe}-*closo*-B_10_H_8_-1-CN-10-(4,4′-Bipyridinium)] (**4c**)

Solid [(η^5^-Cp)(dppe)FeCl] (ref (44), 26.1 mg, 0.047 mmol) and [*closo*-B_10_H_8_-1-CN-10-bipyridyl]^−^ [Bu_4_N]^+^ (**3c[Bu**_**4**_**N]**, 25.4 mg, 0.032 mmol) were placed in a two-neck flask equipped with a condenser. The flask was evacuated and refilled with nitrogen three times. Dry CH_2_Cl_2_ (5 mL) was added by a syringe. The resulting brownish solution was stirred at reflux for 16 h, during which time the mixture changed color from brown to dark red. After cooling, the flask was opened to air and the mixture was filtered through a silica gel plug. The silica plug was washed with CH_2_Cl_2_/MeCN (10:1) until the first red-orange band was fully eluted. The eluent was concentrated to dryness to give a red residue, which was recrystallized from AcOEt giving 23.2 mg (60% yield) of solvate **4c·AcOEt** as red crystals: ^1^H NMR (400 MHz, CD_2_Cl_2_) δ 0.00–1.20 (br m, 8H), 2.40–2.64 (m, 4H), 4.38 (s, 5H), 7.29 (t, *J* = 8.3 Hz, 4H), 7.40 (t, *J*_1_ = 7.4 Hz, 4H), 7.43–7.55 (m, 8H), 7.66 (dd, *J*_1_ = 4.5 Hz, *J*_2_ = 1.6 Hz, 2H), 7.91–8.01 (m, 6H), 8.84 (dd, *J*_1_ = 4.5 Hz, *J*_2_ = 1.5 Hz, 2H), 9.53 (d, *J* = 6.8 Hz, 2H), AcOEt signals (0.125 equiv): δ 1.23 (t, *J* = 7.1 Hz, 0.375H), 2.00 (s, 0.375H), and 4.08 (q, *J* = 7.1 Hz, 0.25H) (NMR recorded after overnight drying of the crystals under high vacuum (0.1 mmHg)); ^13^C{^1^H} NMR (101 MHz, CD_2_Cl_2_) δ 27.7 (t, *J* = 21 Hz), 78.8, 121.7, 123.7, 128.9 (t, *J* = 4.4 Hz), 129.3 (t, *J* = 4.6 Hz), 130.4, 130.6, 132.5 (t, *J* = 4.6 Hz), 133.4 (t, *J* = 4.6 Hz), 134.6 (t, *J* = 21 Hz), 138.3 (t, *J* = 20 Hz), 142.9, 148.7, 151.1, 151.6; ^11^B NMR (128 MHz, CD_2_Cl_2_) δ −27.3 – −22.5 (m, with maxima at −25.0 and −24.4, 8B), −6.3 (1B), 19.3 (1B); ^31^P{^1^H} NMR (162 MHz, CD_2_Cl_2_) δ 98.6; IR (ATR) ν 3054, 2486 (BH), 2196 (CN), 1731, 1592, 1432, 1095, 691 cm^–1^; UV (CH_2_Cl_2_) λ_max_ (log ε) 243.6 (4.68), 380.2 (4.03) nm; HRMS (ESI, +) *m*/*z* calcd. for C_42_H_45_B_10_FeN_3_O_2_P_2_: 819.3369, found: 819.3442. Anal. Calcd for C_42_H_45_B_10_FeN_3_P_2_: C, 61.69; H, 5.55; N, 5.14. Found: C, 61.66; H, 5.60; N, 5.18.

#### [{(η^5^-Cp)(dppe)Fe}-*closo*-B_10_H_8_-1-CN-10-Pyrazinium] (**4d**)

Following the procedure for preparation of **4c** (using pure CH_2_Cl_2_ for chromatography), solvate **4d·AcOEt** was obtained from [(η^5^-Cp)(dppe)FeCl] (ref (44), 17.8 mg, 0.032 mmol) and [*closo*-B_10_H_8_-1-CN-10-pyrazine]^−^ [Bu_4_N]^+^ (**3d[Bu**_**4**_**N]**, 15.0 mg, 0.032 mmol) as red crystals in 85% yield (20.1 mg), which were dried in vacuum (0.1 mmHg) overnight: ^1^H NMR (400 MHz, CD_2_Cl_2_) δ 0.10–1.10 (br m, 8H), 2.42–2.61 (m, 4H), 4.38 (s, 5H), 7.25–7.31 (m, 4H), 7.40 (t, *J* = 7.4 Hz, 4H), 7.43–7.54 (m, 8H), 7.92–7.98 (m, 4H), 9.05 (dd, *J*_1_ = 3.0 Hz, *J*_2_ = 1.5 Hz, 2H), 9.37 (dd, *J*_1_ = 3.1 Hz, *J*_2_ = 1.4 Hz, 2H), AcOEt signals (0.5 equiv): δ 1.23 (t, *J* = 7.4 Hz, 1.5H), 2.00 (s, 1.5H), and 4.08 (q, *J* = 7.4 Hz, 1H); ^13^C{^1^H} NMR (101 MHz, CD_2_Cl_2_) δ 27.7 (t, *J* = 21 Hz), 78.9, 129.0 (t, *J* = 4.6 Hz), 129.3 (t, *J* = 4.8 Hz), 130.4, 130.6, 132.4 (t, *J* = 4.7 Hz), 133.4 (t, *J* = 4.8 Hz), 134.5 (t, *J* = 21 Hz), 138.2 (t, *J* = 20 Hz), 140.7, 148.4; AcOEt signals (0.5 equiv): δ 14.4, 21.2, and 60.6; ^11^B NMR (128 MHz, CD_2_Cl_2_) δ −24.5 (d, *J* = 145 Hz, 4B), −23.2 (d, *J* = 143 Hz, 4B), −3.8 (1B), 18.2 (1B); ^31^P{^1^H} NMR (162 MHz, CD_2_Cl_2_) δ 98.5; IR (ATR) ν 2921, 2491 (BH), 2185 (CN), 1731, 1602, 1433, 1095, 691 cm^–1^; UV (CH_2_Cl_2_) λ_max_ (log ε) 425.6 (3.85) nm; HRMS (ESI, + ) *m*/*z* calcd. for C_36_H_41_B_10_N_3_P_2_Fe: 743.3056, found: 743.3077. Anal. Calcd for C_36_H_41_B_10_FeN_3_P_2_: C, 58.30; H, 5.57; N, 5.67. Found: C, 58.14; H, 5.49; N, 5.71.

#### [{(η^5^-Cp)(dppe)Fe-CN}-1-*closo*-B_10_H_8_-10-{4,4′-Bipyridinium-Cu(pdc)}] (**5c**)

Triaqua-(2,6-pyridinedicarboxylato)-copper(II) (Cu(pdc)(aq)_3_, 1.07 mg, 0.0038 mmol) was dissolved on heating in MeOH (250 μL). Complex **4c** (3.13 mg, 0.0038 mmol) was dissolved on heating in MeOH (1 mL). Both warm solutions were combined and cooled down. After *ca*. 15 min small thin needles of **5c** were formed which were filtered and dried giving 3.52 mg (88% yield) of dark brown complex **5c**: MS (ESI,+) *m*/*z* 519 (max, 100); HRMS (ESI,+) *m*/*z* calcd. for C_49_H_48_B_10_CuFeN_4_O_4_P_2_: [M–OH] 1047.2768, found: 1047.2802. Anal. Calcd for C_49_H_49_B_10_CuFeN_4_O_5_P_2_: C, 55.35 H, 4.64; N, 5.27. Found: C, 55.72; H, 4.58; N, 5.07.

#### [1,4-Bis-(*closo*-B_10_H_8_-10-CN-1-)-Pyrazinium]^2–^2[Bu_4_N]^+^ (**9[Bu**_**4**_**N**])

Product **9[Bu**_**4**_**N]** was obtained in 7% yield (4.2 mg) using **7[Bu**_**4**_**N]** (59 mg, 0.10 mmol) and pyrazine (40 mg, 0.50 mmol) as a dark blue solid: *R*_f_ = 0.25 (5:1 CH_2_Cl_2_/MeCN); ^1^H NMR (500 MHz, acetone-*d*_6_) δ 0.40–1.90 (br m, 16H), 0.98 (t, *J* = 7.4 Hz, 24H), 1.44 (sext, *J* = 7.5 Hz, 16H), 1.83 (pseudo quint, *J* = 8.0 Hz, 16H), 3.45 (pseudo t, *J* = 8.6 Hz, 16H), 9.83 (s, 4H); ^13^C{^1^H} NMR (126 MHz, acetone-*d*_6_) δ 13.9, 20.4, 24.5, 59.5, 144.2. The signal corresponding to the carbon of the CN group was not observed; ^11^B NMR (160 MHz, acetone-*d*_6_) δ −20.8 (d, *J* = 137 Hz, 4B), −18.2 (d, *J* = 134 Hz, 4B), 4.8 (s, 1B), 20.3 (s, 1B); IR (ATR) *v* 2959, 2873, 2464 (BH), 2176 (CN), 1468, 1379, 879, 738 cm^–1^; UV (CH_3_CN) λ_max_ (log ε) 269.0 (3.82), 554.0 (4.18) nm; HRMS (ESI, −) *m*/*z* calcd. for C_6_H_21_B_20_N_4_: 369.3627, found: 369.3645.

### X-ray Data Collection

Single-crystal X-ray diffraction measurements were conducted at 100.0(2) K using the Cu*K*_α_ radiation (λ = 1.54184 Å). The data were integrated using CrysAlisPro program.^61^ Intensities for absorption were corrected using SCALE3 ABSPACK scaling algorithm implemented in CrysAlisPro program.^61^

## Supporting Information Available

Additional synthetic details, copies of NMR spectra, XRD data collection details, and UV–vis data (PDF)
