# Supplementary material for: [closo-B10H8-1-CN-10-Azinium]− Anions: Photoactive Heteroditopic Ligands for Metal Complexes
Source: Inorg Chem. 2024 Sep 6;63(38):17774–84. doi: 10.1021/acs.inorgchem.4c02670 (PMC11423411; doi:10.1021/acs.inorgchem.4c02670)
Supplement: Supplementary file 1 — ic4c02670_si_001.pdf [file ic4c02670_si_001.pdf]

Supporting Information for

[*closo*-$B_{10}H_8$-1-CN-10-Azinium]$^-$ anions: Photoactive heteroditopic ligands for metal complexes

Rafał Jakubowski,[a,b] Mustapha B. Abdulmojeed,[a] Oleksandr Hietsoi,[a] Andrienne C. Friedli,*[a] and Piotr Kaszyński*[a,b,c]

[a] Department of Chemistry, Middle Tennessee State University, Murfreesboro, TN, 37130

[b] Centre of Molecular and Macromolecular Studies, Polish Academy of Sciences, 90-363 Łódź, Poland.

[c] Faculty of Chemistry, University of Łódź, 91-403 Łódź, Poland

Table of Content:

1. Additional synthetic details ….S2
2. NMR spectra S3
3. XRD data collection and refinement S19
    a) general comments S19
    *b) structure solution and refinement for **2–4*** S19
    *c) attempted preparation of crystals of **5c*** S26
4. Electronic absorption and emission spectra ….S28
5. Electrochemical data ….S35
6. Partial output data for TD-DFT calculations S36
7. Archive for DFT results S38
8. References S46

1. Additional synthetic details

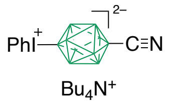

Revised procedure for the preparation of [*closo*-$B_{10}H_8$-10-IPh-1-CN][Bu_4N] (7[Bu_4N]).[1] It was prepared according to the literature procedure[1] from [*closo*-$B_{10}H_8$-1,10-$(IPh)_2$][2] (**6**, 1.572 g, 3.00 mmol) and $[Bu_4N]^+[CN]^-$ (885 mg, 3.30 mmol). Substrates were dissolved in THF/MeCN mixture (1:1, 60 mL) and stirred for 13 h at 55 °C. The reaction mixture was evaporated avoiding excess heat. The crude mixture was purified on passivated with $[Bu_4N]^+[HSO_4]^-$ silica gel using as eluent CH_2Cl_2 (elution of unreacted [*closo*-$B_{10}H_8$-1,10-$(IPh)_2$]), followed by CH_2Cl_2/MeCN (20:1) giving 1.401 g (47% yield, 47–50% in several runs) of pure **7[Bu_4N]** as a brownish oil which solidified on standing, which was used in the next step without further purification.

Note: Longer heating results in formation of large quantities of side products.

Preparation of triaqua-(2,6-pyridinedicarboxylato)-copper(II) (Cu(pdc)(aq)$_3$). 2,6-Pyridinecarboxylic acid (167.1 mg, 1 mmol) was suspended in H_2O (3 mL) and a solution of NaOH (80.0 mg, 2 mmol) in H_2O (2 mL) was added. The resulting mixture was heated until all solids dissolved and $CuCl_2•2H_2O$ (170.5 mg, 1 mmol) was added in one portion. After *ca*. 15 min. blue crystals formed which were filtered off, washed with H_2O and dried in air giving 232.7 mg (82% yield) of triaqua-(2,6-pyridinedicarboxylato)-copper(II). The structure and composition of the solid was confirmed with XRD analysis.

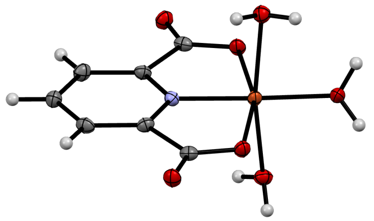

Crystal Data: $C_7H_9CuNO_7$, M_r = 282.69, monoclinic, $P2_1/n$ (No. 14), a = 6.3868(2) Å, b = 23.1162(8) Å, c = 6.8136(2) Å, β = 106.352(4)°, α = γ = 90°, V = 965.26(6) Å^3, T = 99.8(6)K, Z= 4, Z'= 1, μ(Cu K$_\alpha$)= 3.483, 4546 reflections measured, 1947 unique (R_{int} = 0.0344) which were used in all calculations. The final wR_2 was 0.1216 (all data) and R_1 was 0.0411 (I > 2(I)).

The unit cell parameters were found to be nearly identical with those reported in the literature.[3]

2. NMR spectra.

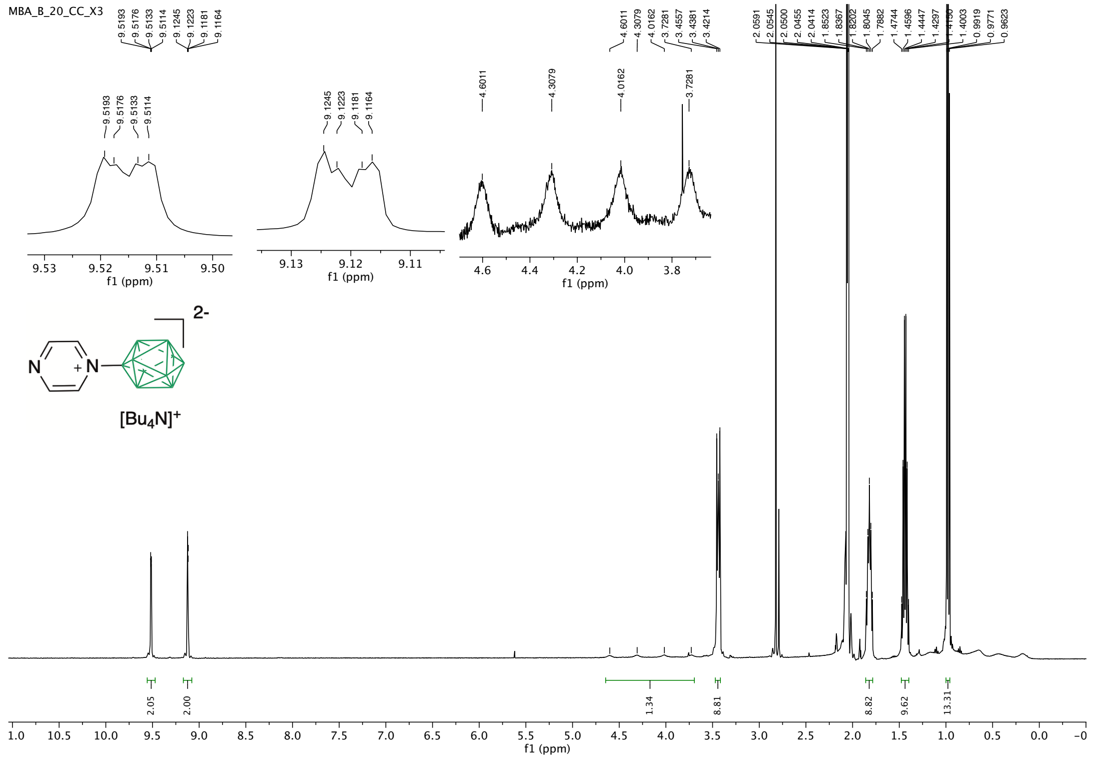

Figure S1: ^{1}H NMR spectrum of compound **2d[Bu$_4$N]** (500 MHz, acetone-d_6).

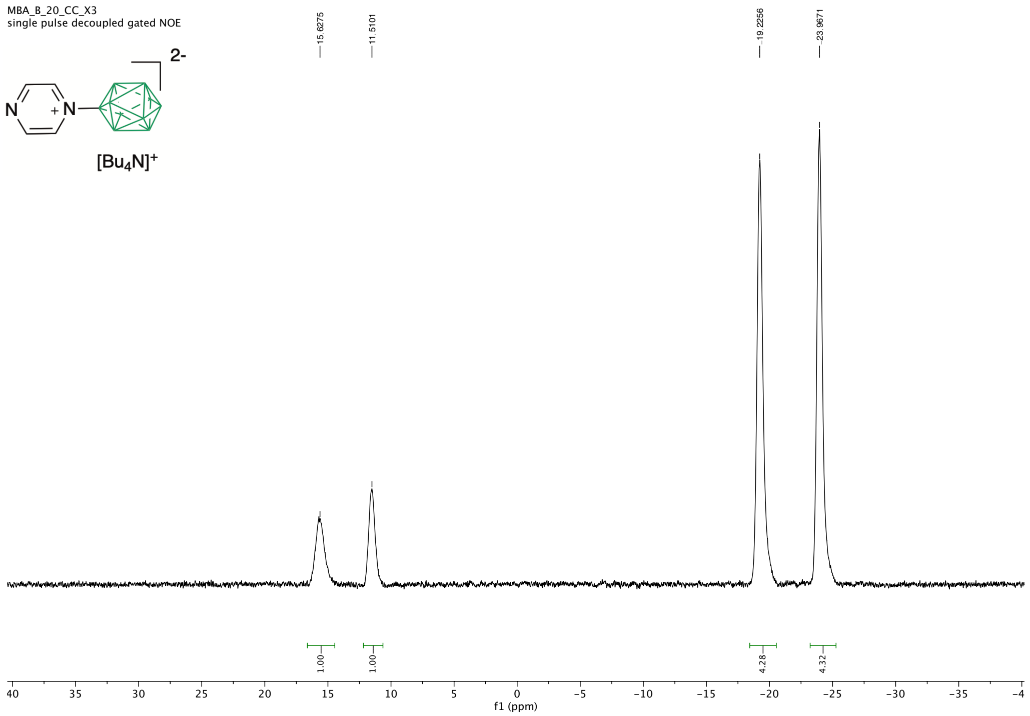

Figure S2: ^{11}B{^{1}H} NMR spectrum of compound **2d[Bu$_4$N]** (160 MHz, acetone-d_6).

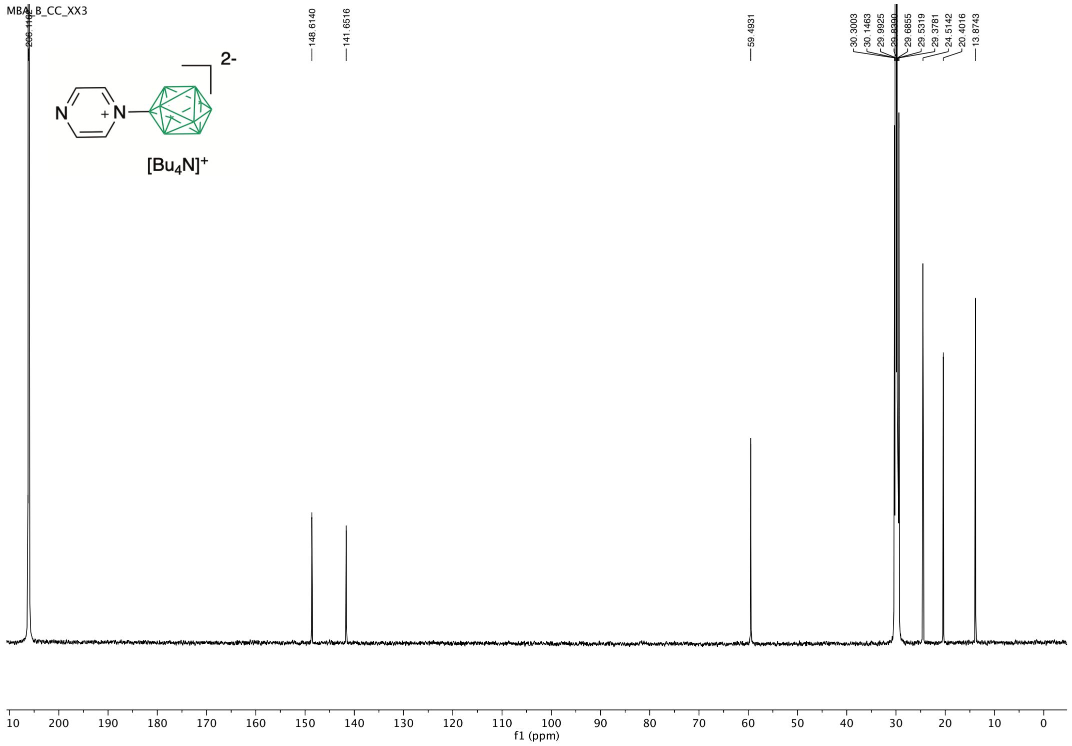

Figure S3: $^{13}C\{^1H\}$ NMR spectrum of compound **2d[Bu$_4$N]** (126 MHz, acetone-d_6).

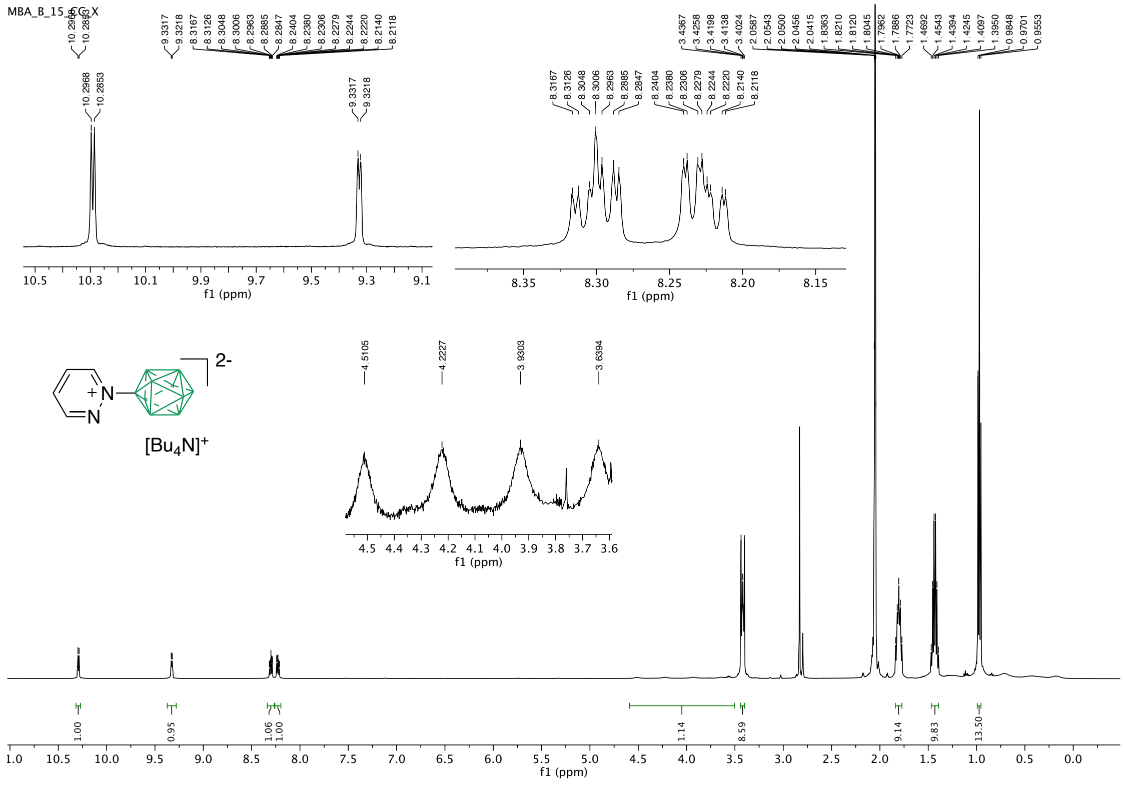

Figure S4: ^1H NMR spectrum of compound **2f[Bu$_4$N]** (500 MHz, acetone-d_6).

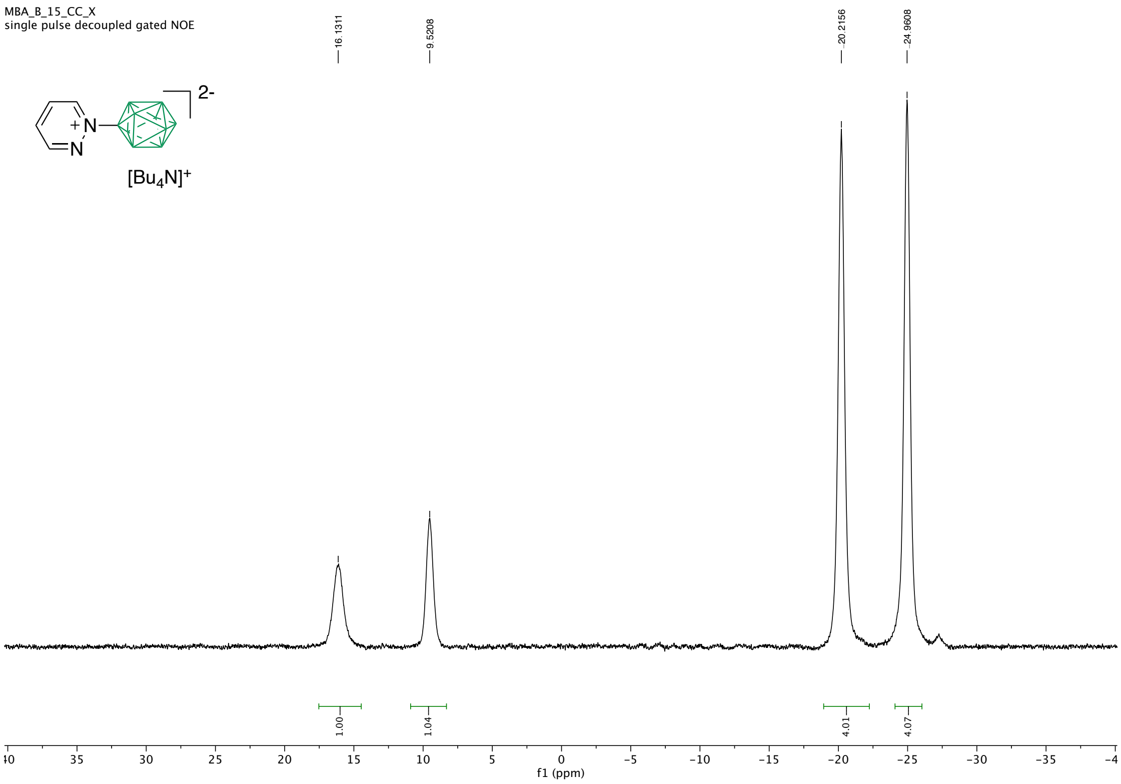

Figure S5: $^{11}B\{^{1}H\}$ NMR spectrum of compound **2f[Bu₄N]** (160 MHz, acetone-*d*$_6$).

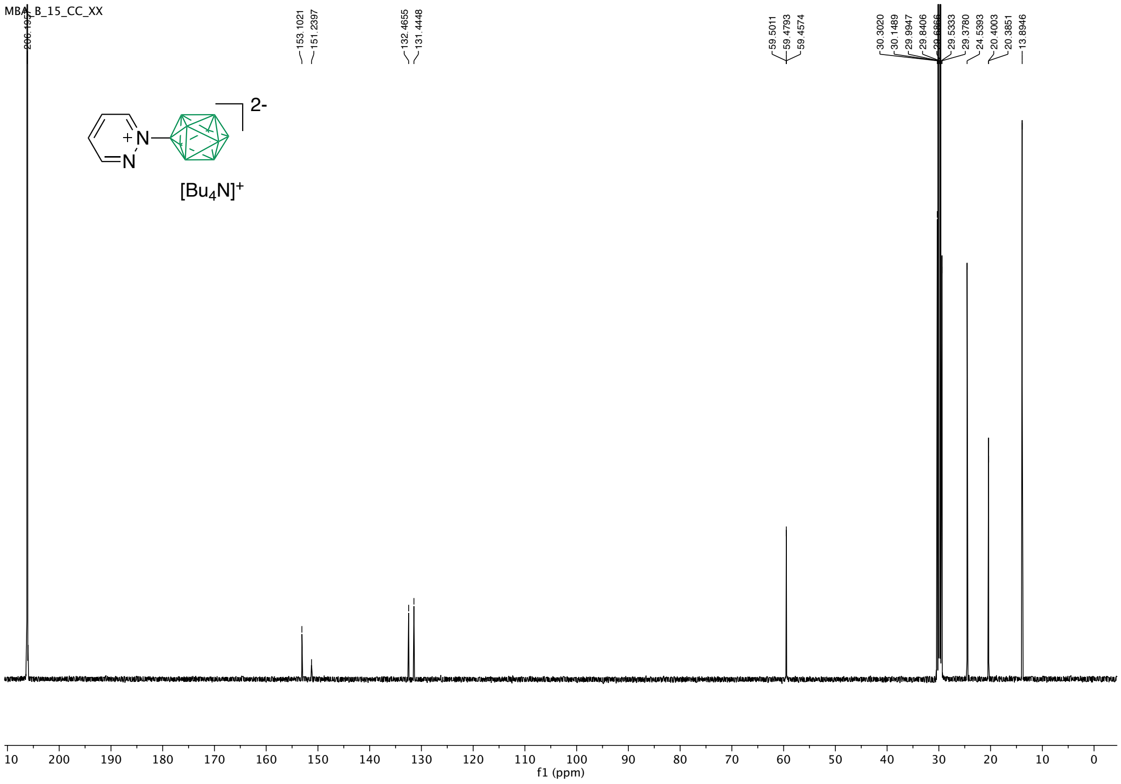

Figure S6: $^{13}C\{^{1}H\}$ NMR spectrum of compound **2f[Bu₄N]** (126 MHz, acetone-*d*$_6$).

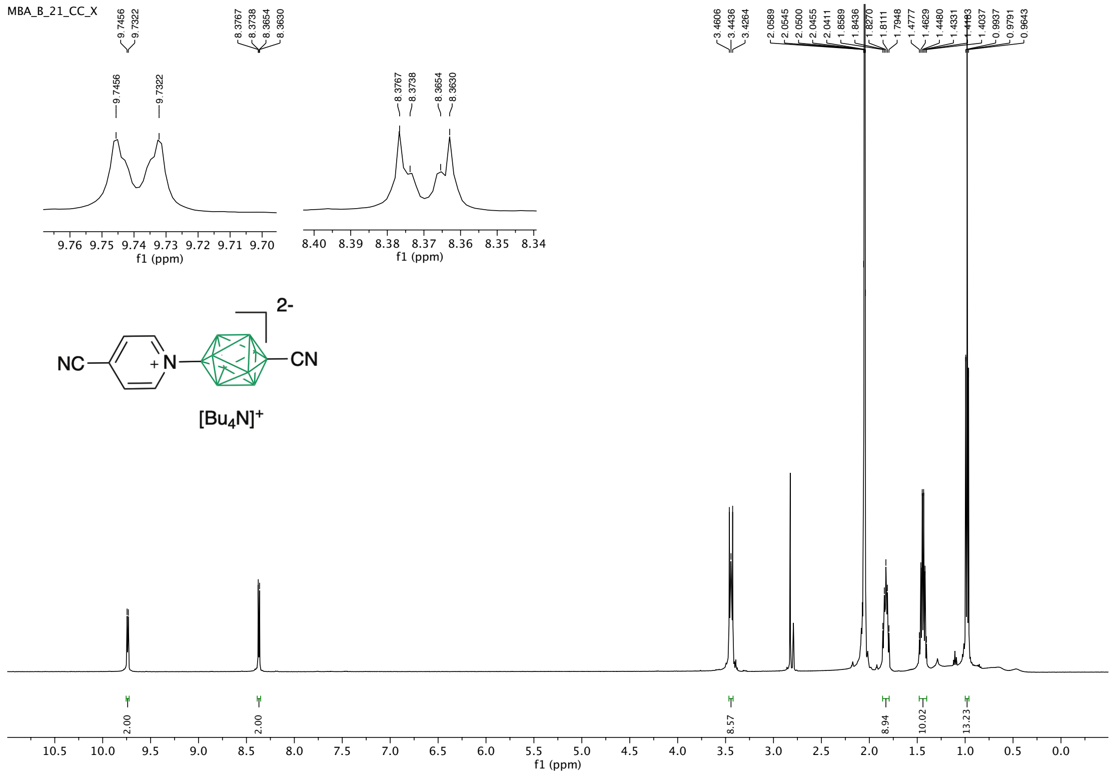

Figure S7: ^{1}H NMR spectrum of compound **3b[Bu$_4$N]** (500 MHz, acetone-d_6).

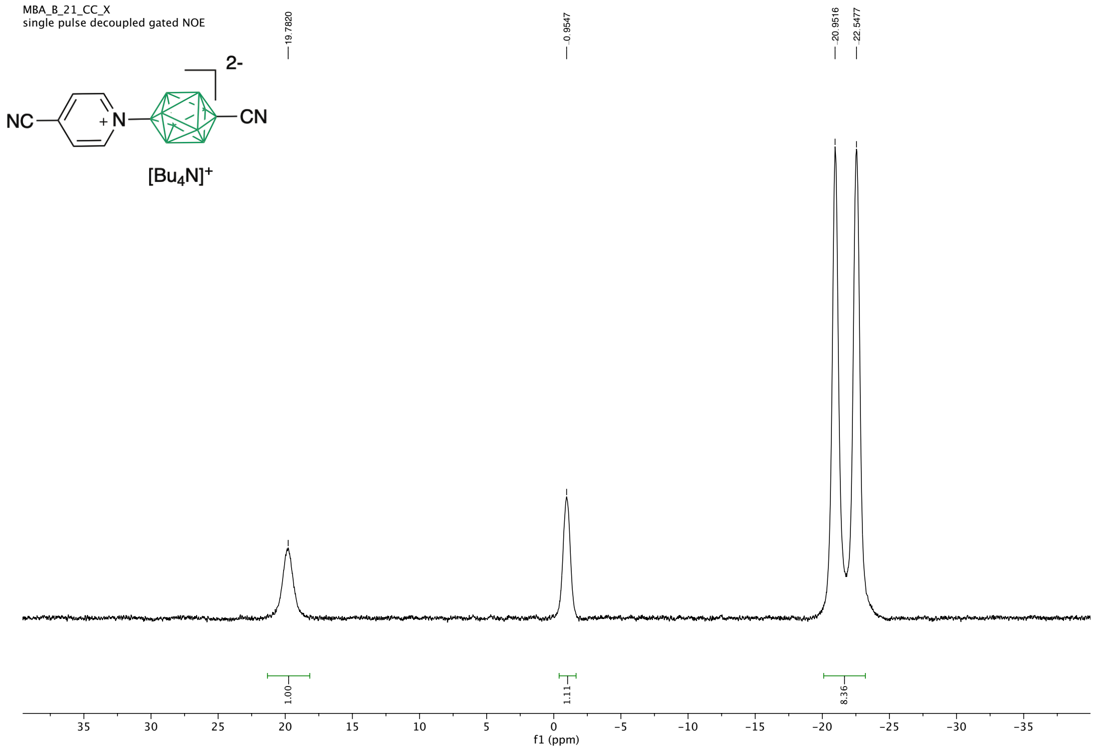

Figure S8: ^{11}B{^{1}H} NMR spectrum of compound **3b[Bu$_4$N]** (160 MHz, acetone-d_6).

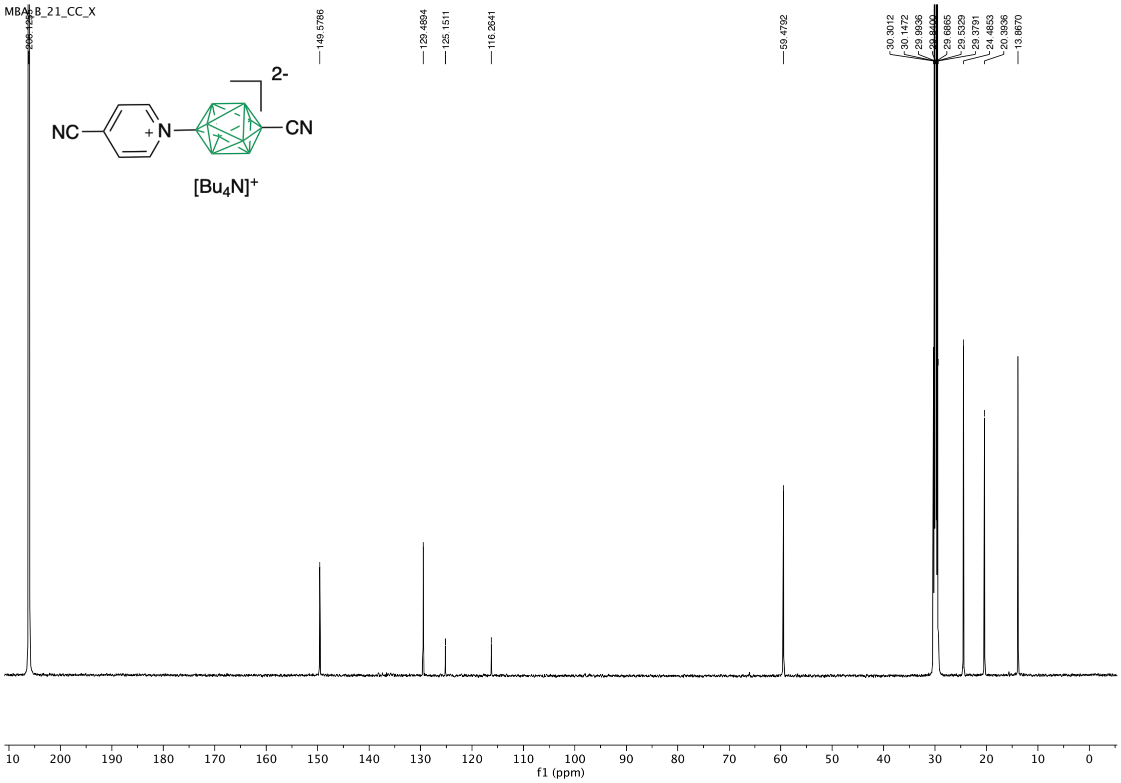

Figure S9: $^{13}C\{^{1}H\}$ NMR spectrum of compound **3b[Bu4N]** (126 MHz, acetone-d_6).

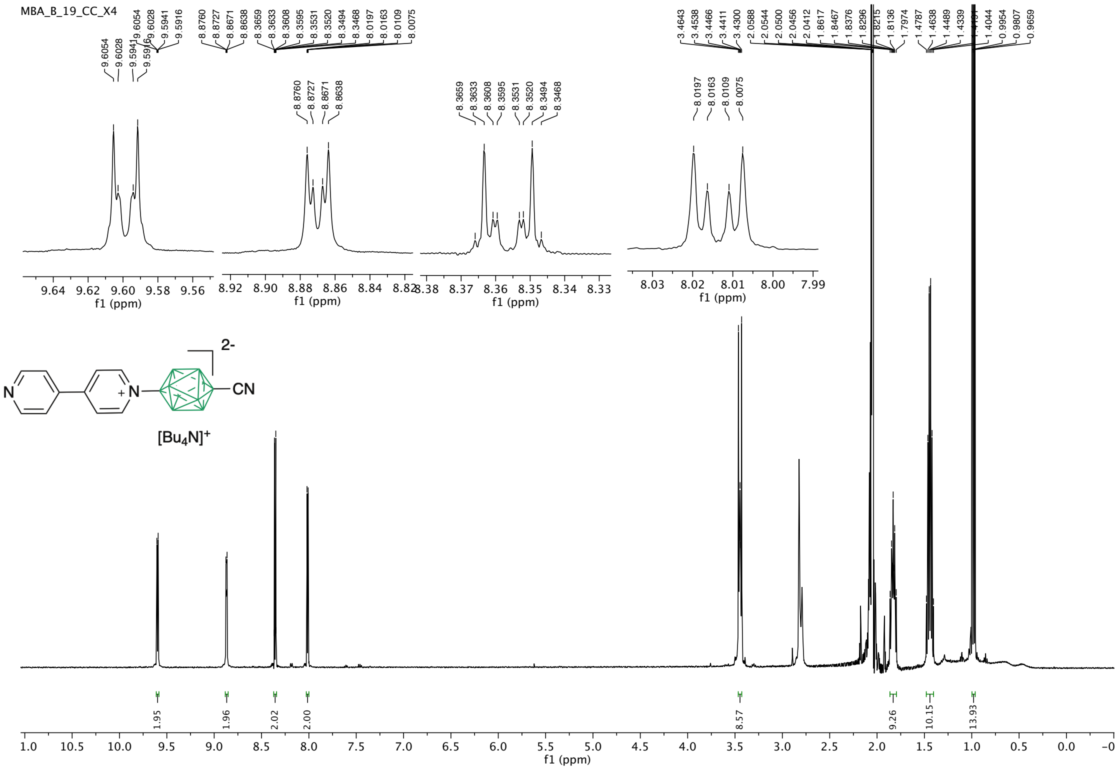

Figure S10: ^{1}H NMR spectrum of compound **3c[Bu4N]** (500 MHz, acetone-d_6).

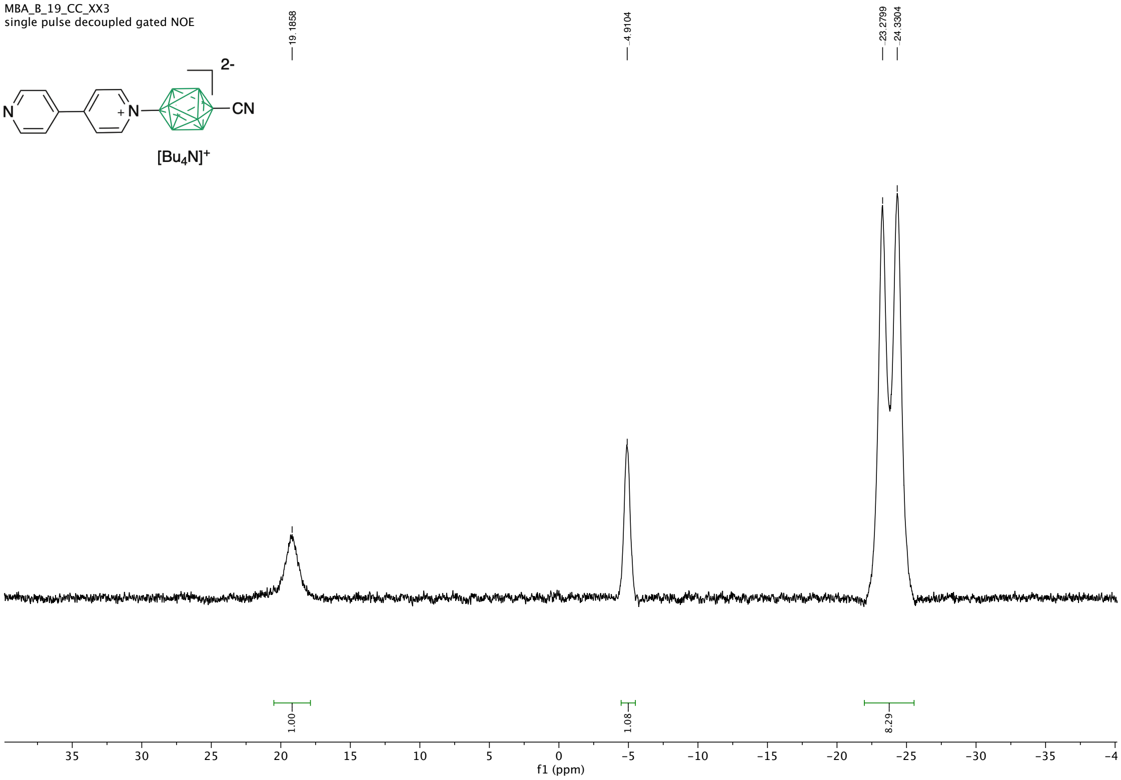

Figure S11: $^{11}B\{^{1}H\}$ NMR spectrum of compound **3c[Bu₄N]** (160 MHz, acetone-d_6).

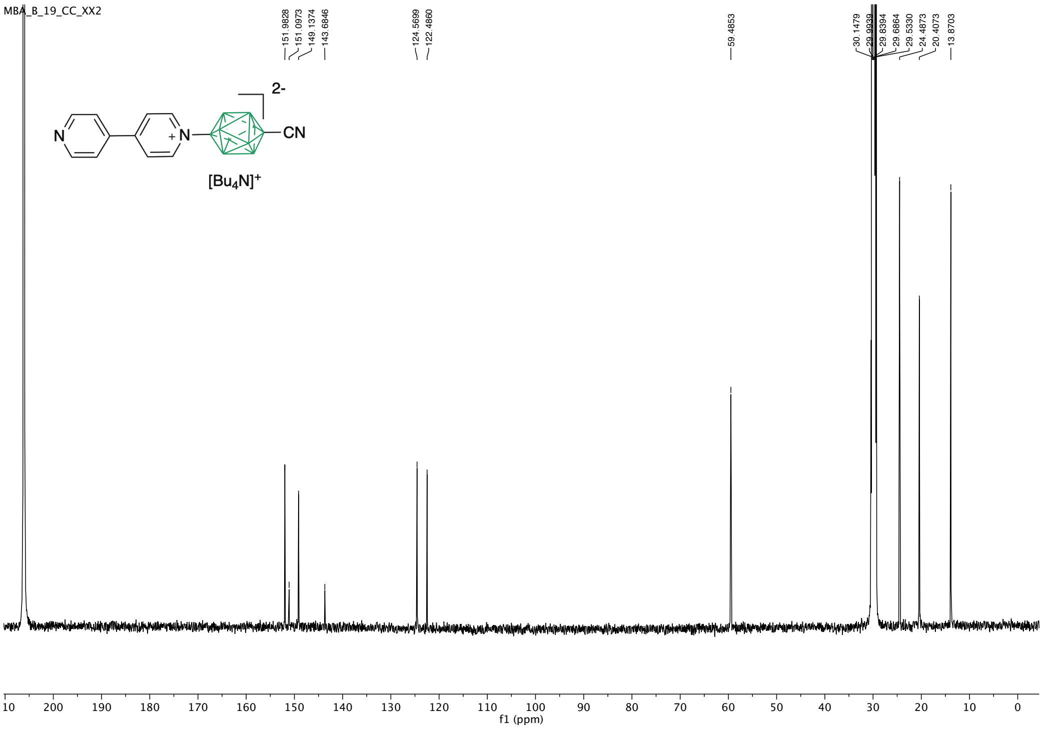

Figure S12: $^{13}C\{^{1}H\}$ NMR spectrum of compound **3c[Bu₄N]** (126 MHz, acetone-d_6).

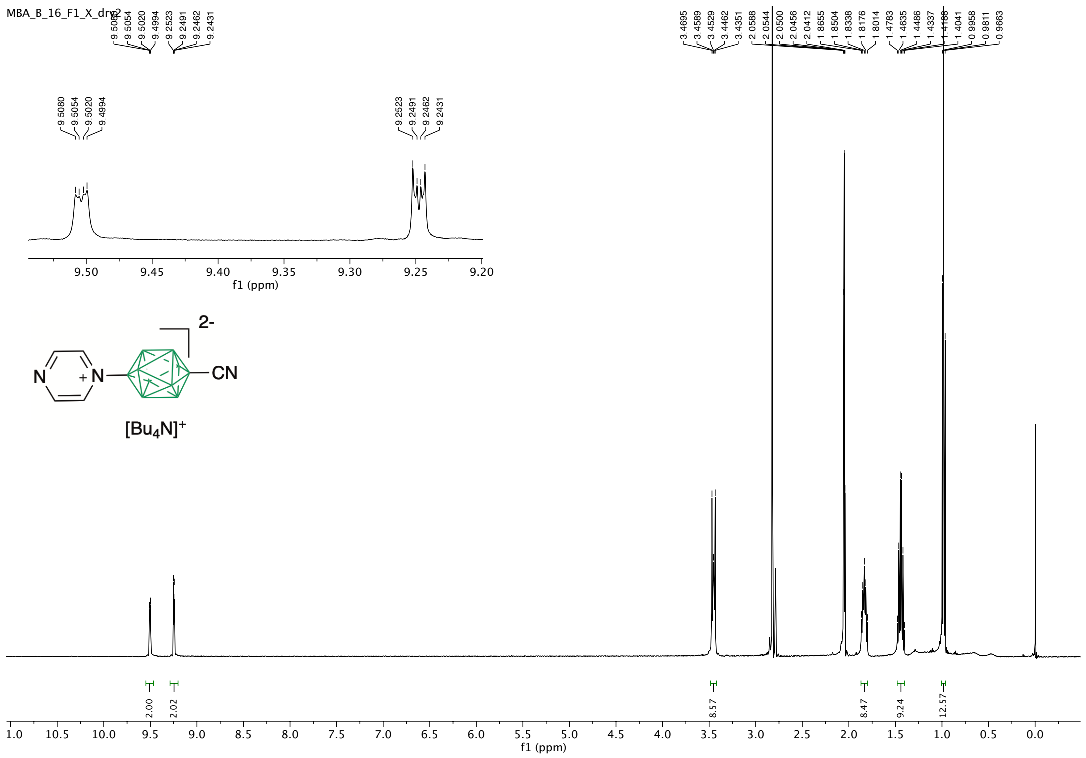

Figure S13: ^{1}H NMR spectrum of compound **3d[Bu$_4$N]** (500 MHz, acetone-d_6).

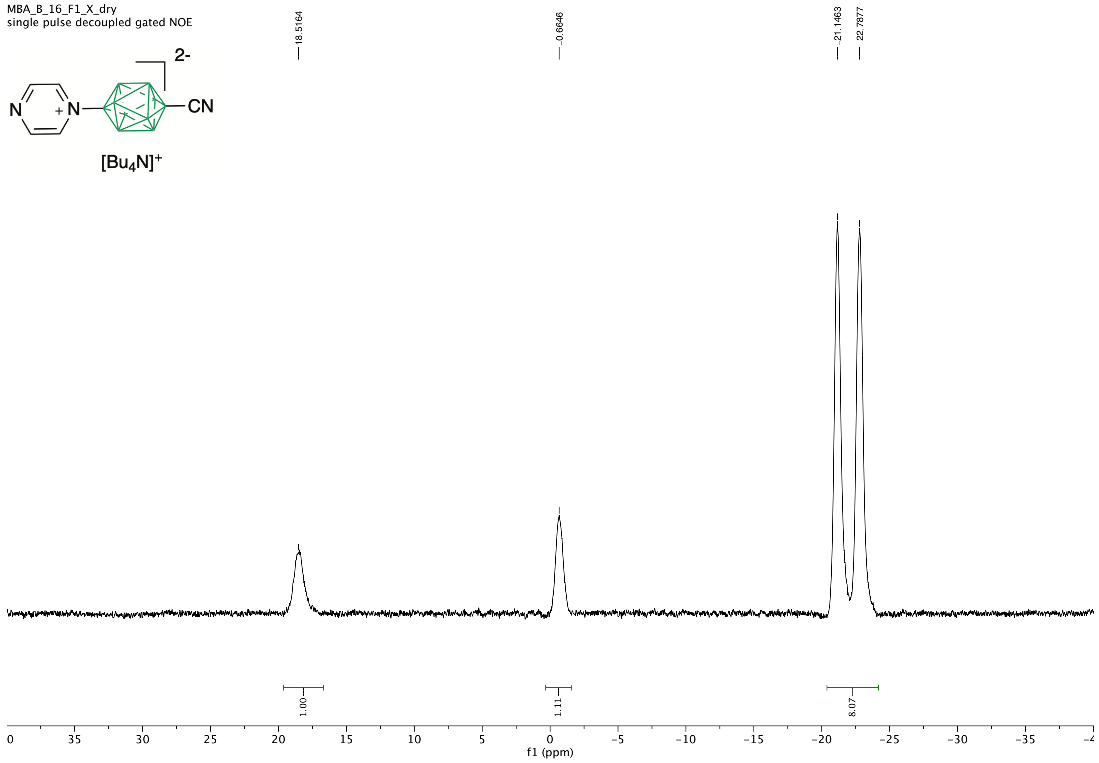

Figure S14: ^{11}B{^{1}H} NMR spectrum of compound **3d[Bu$_4$N]** (160 MHz, acetone-d_6).

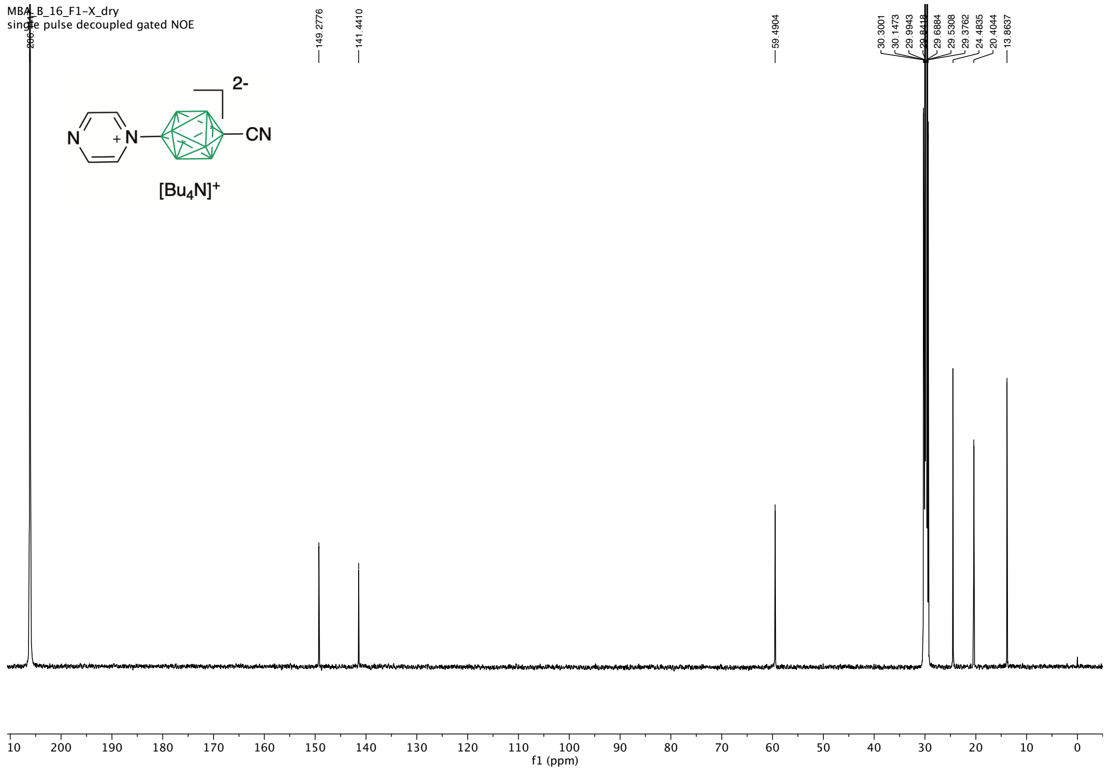

Figure S15: $^{13}C\{^1H\}$ NMR spectrum of compound **3d[Bu₄N]** (126 MHz, acetone-d_6).

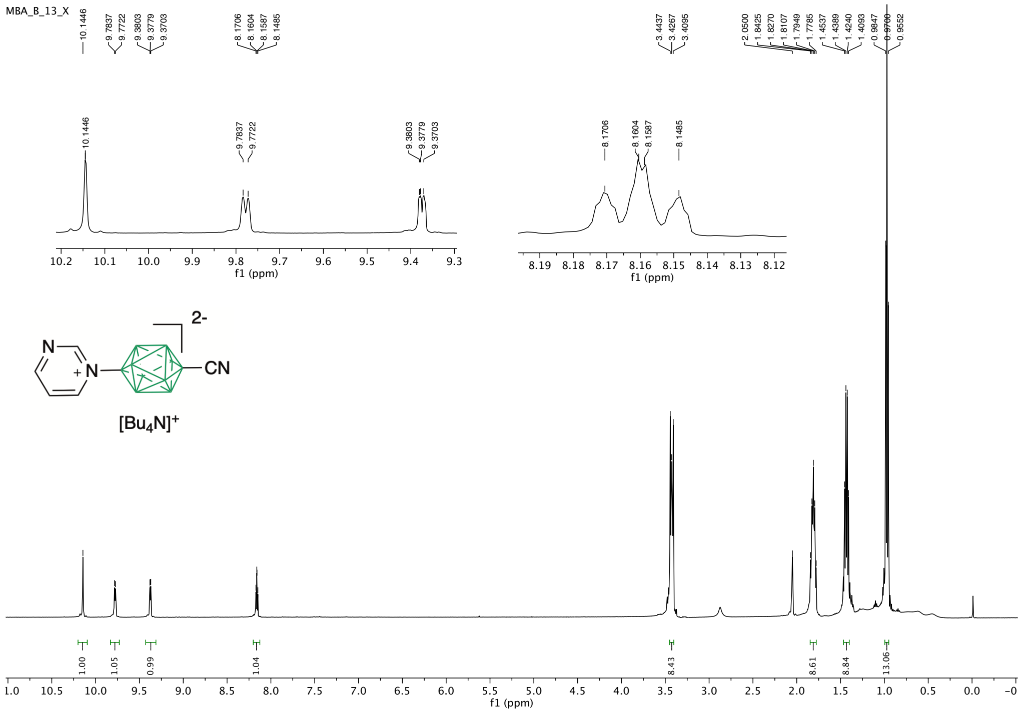

Figure S16: ^1H NMR spectrum of compound **3e[Bu₄N]** (500 MHz, acetone-d_6).

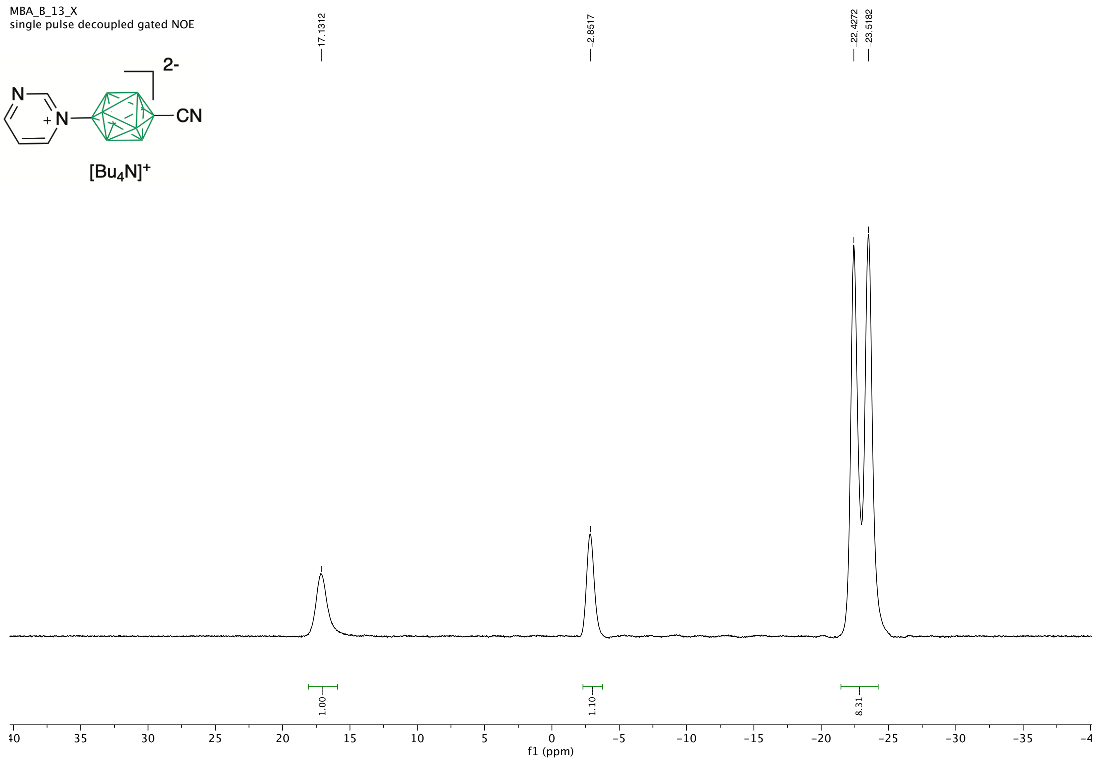

Figure S17: $^{11}B\{^{1}H\}$ NMR spectrum for compound **3e[Bu₄N]** (160 MHz, acetone-d_6).

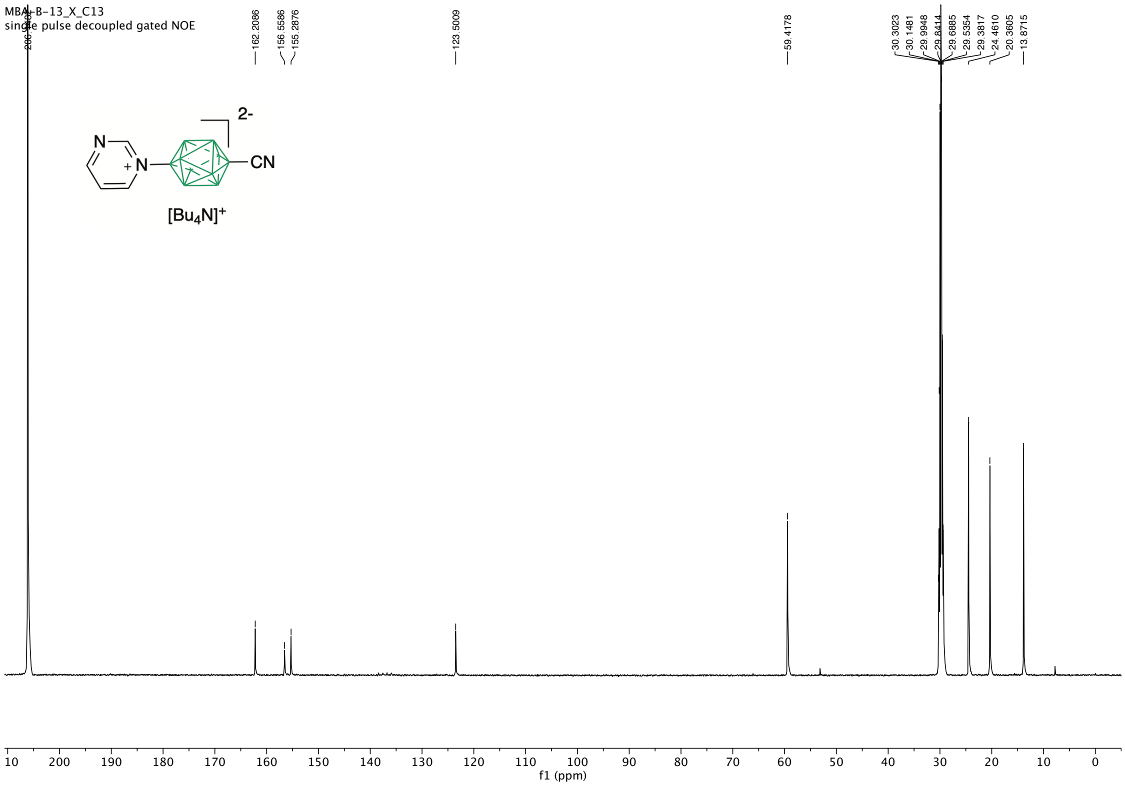

Figure S18: $^{13}C\{^{1}H\}$ NMR spectrum for compound **3e[Bu₄N]** (126 MHz, acetone-d_6).

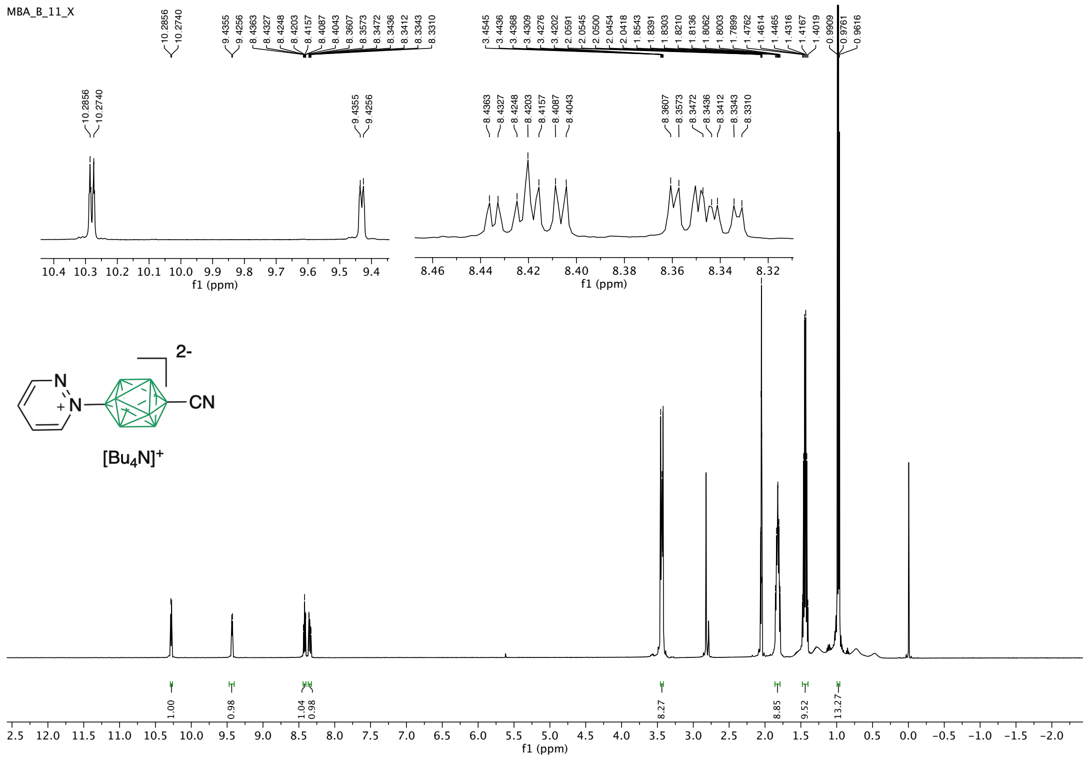

Figure S19: ^{1}H NMR spectrum of compound **3f[Bu$_4$N]** (500 MHz, acetone-d_6).

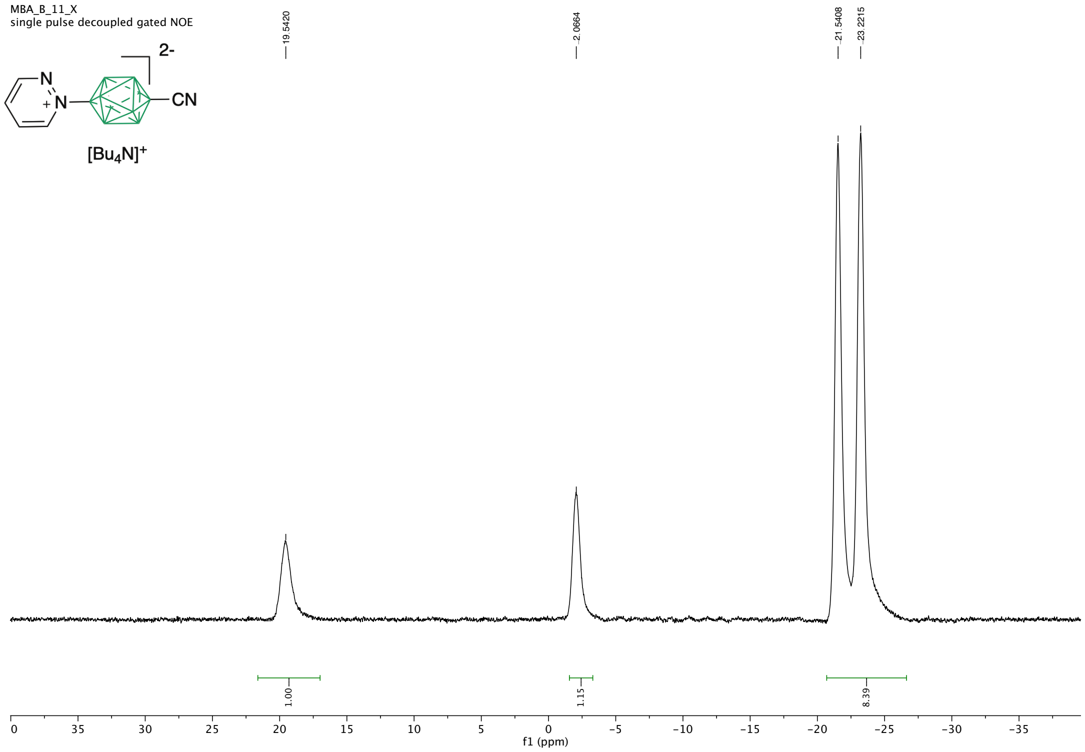

Figure S20: ^{11}B{^{1}H} NMR spectrum of compound **3f[Bu$_4$N]** (160 MHz, acetone-d_6).

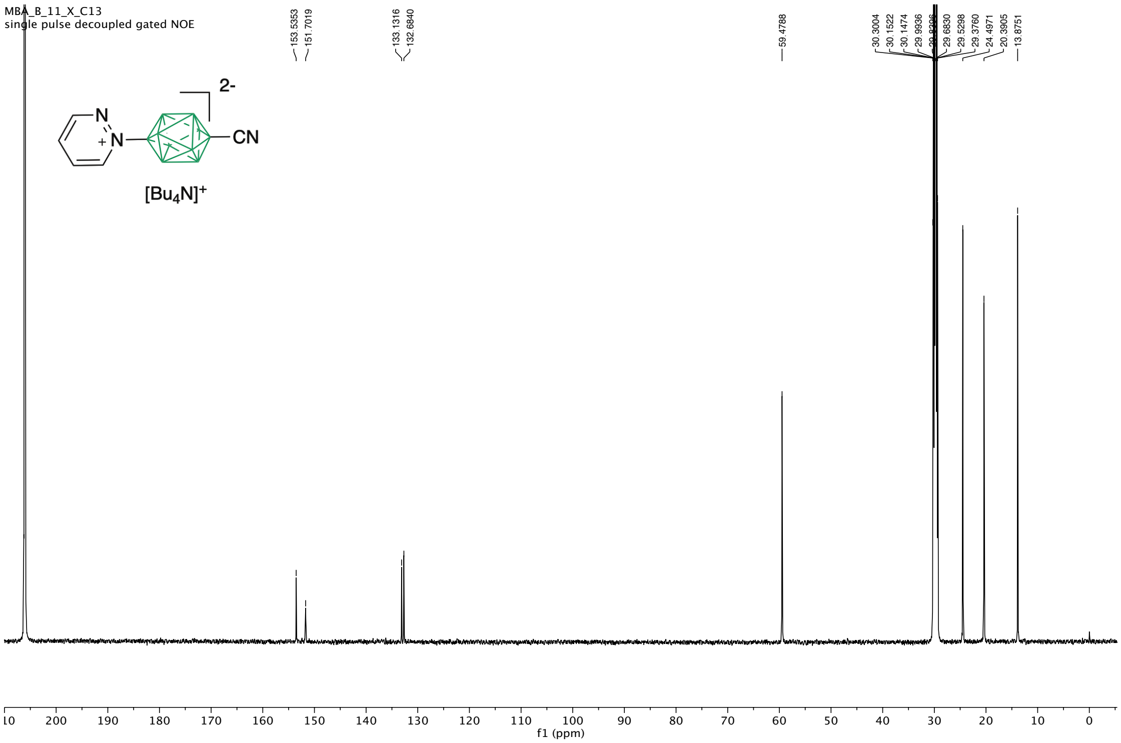

Figure S21: $^{13}C\{^1H\}$ NMR spectrum of compound **3f[Bu4N]** (126 MHz, acetone-d_6).

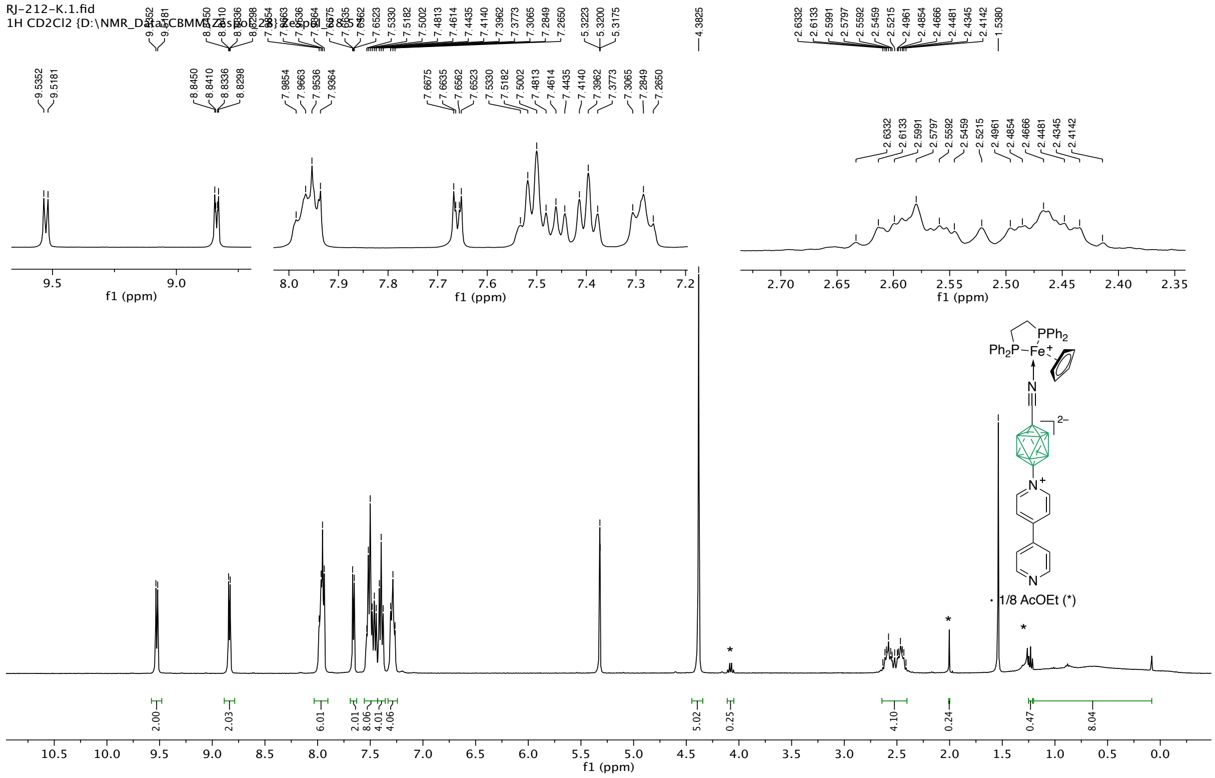

Figure S22: ^1H NMR spectrum of compound **4c** (400 MHz, CD_2Cl_2). The asterisk denotes AcOEt.

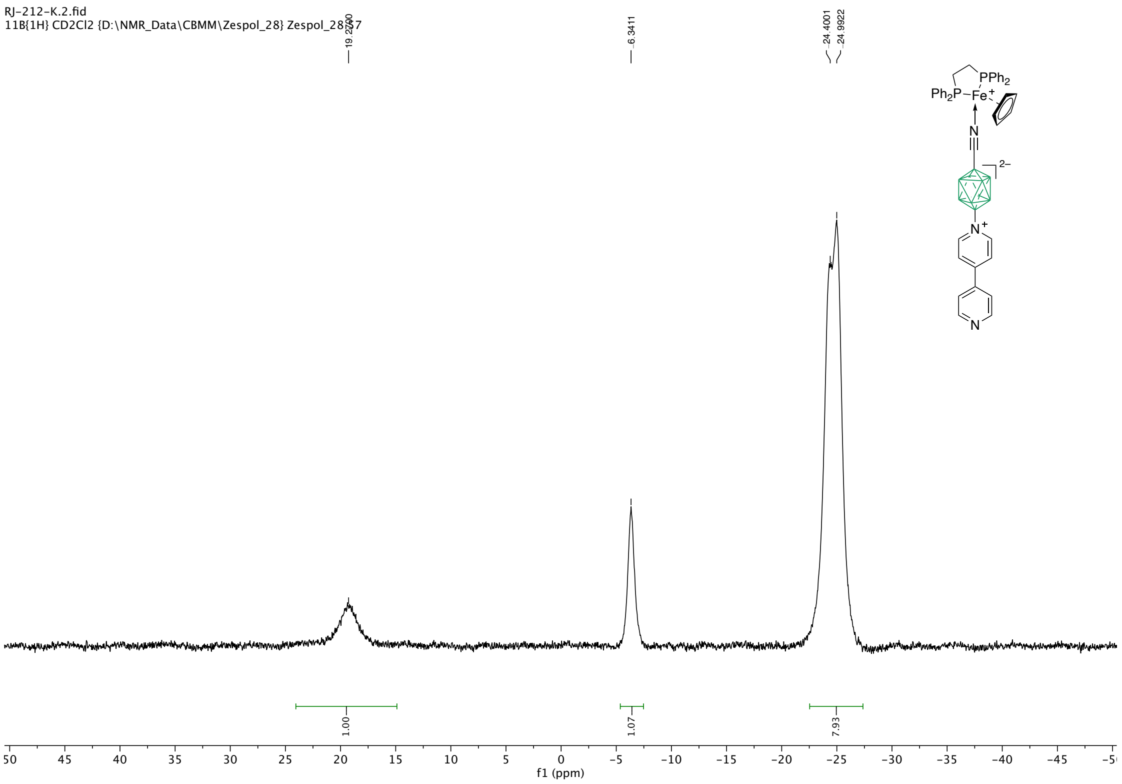

Figure S23: $^{11}B\{^{1}H\}$ NMR spectrum of compound **4c** (128 MHz, CD_2Cl_2).

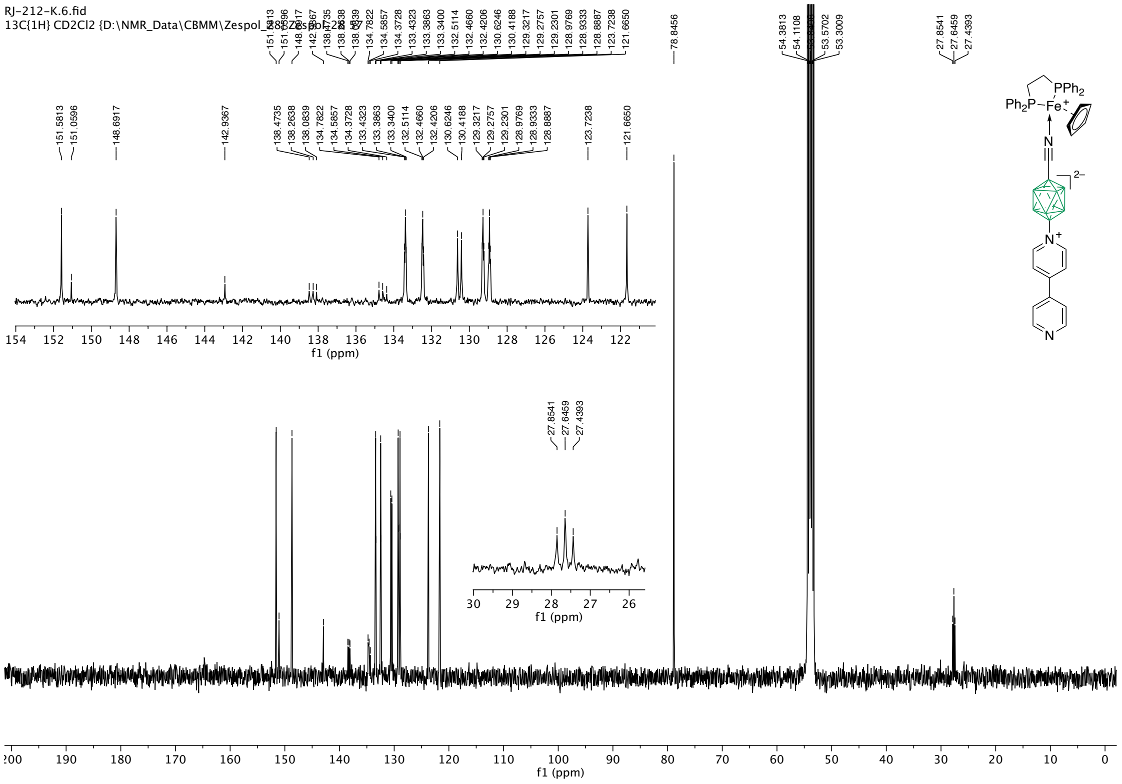

Figure S24: $^{13}C\{^{1}H\}$ NMR spectrum for compound **4c** (101 MHz, CD_2Cl_2).

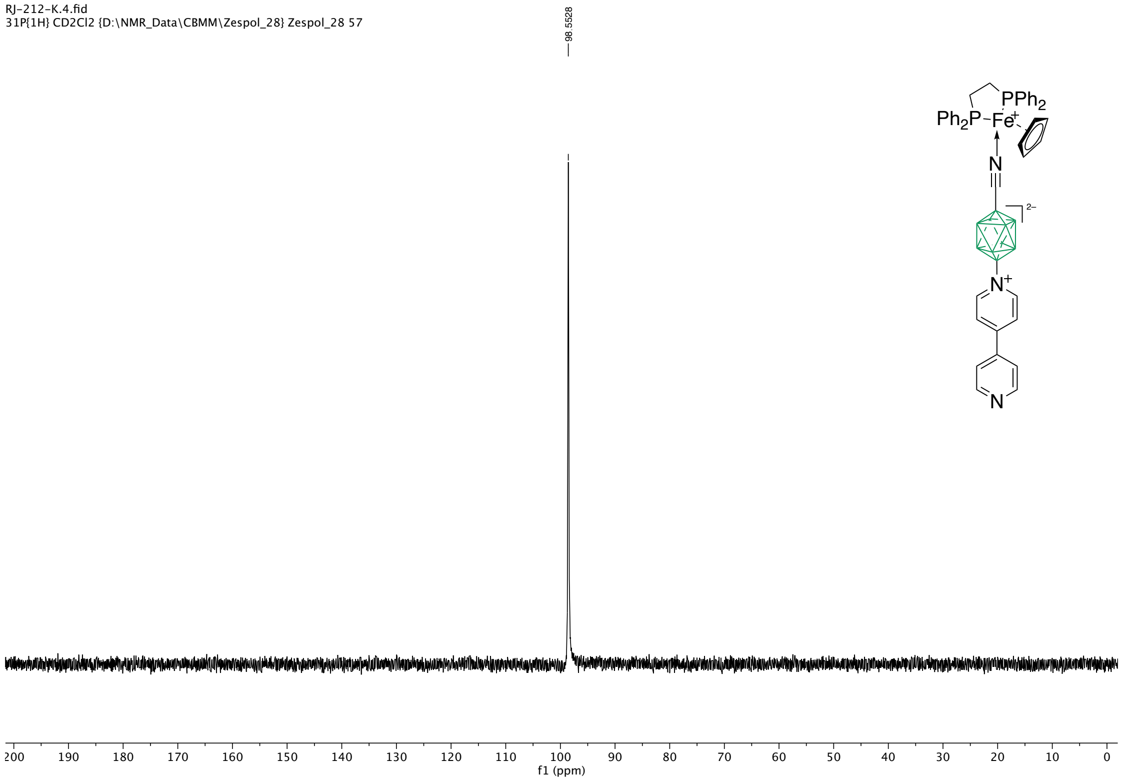

Figure S25: $^{31}P\{^{1}H\}$ NMR spectrum of compound **4c** (162 MHz, CD_2Cl_2).

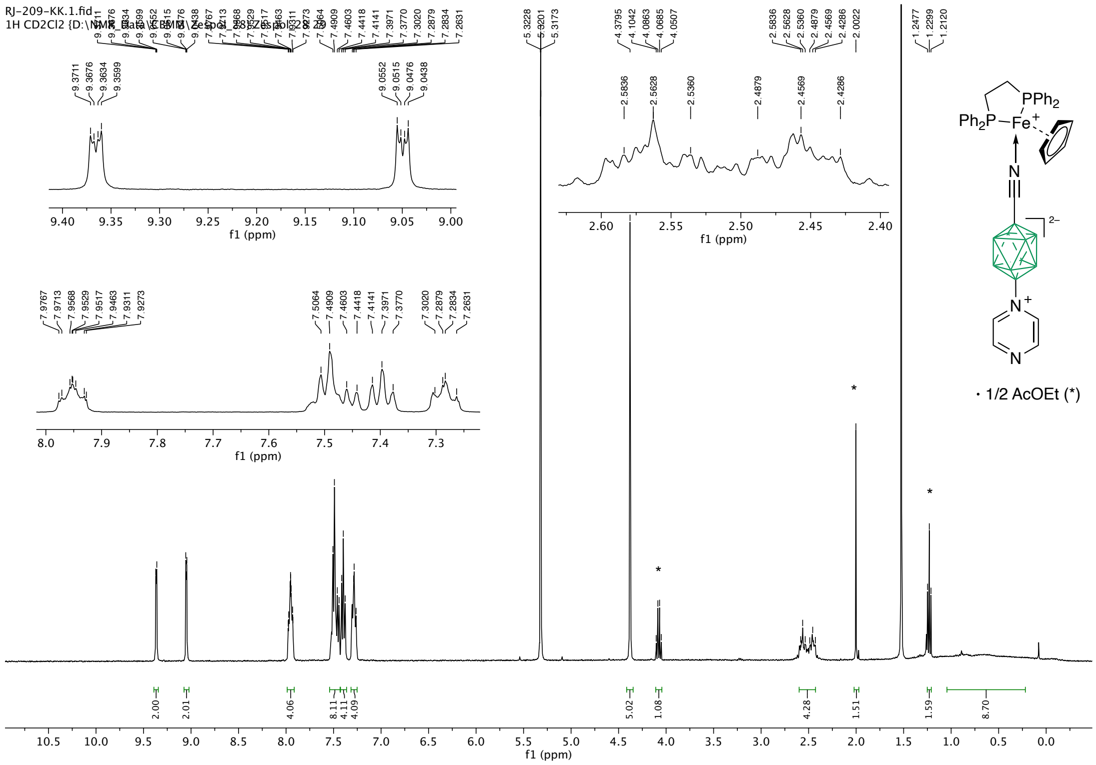

Figure S26: ^{1}H NMR spectrum of compound **4d** (400 MHz, CD_2Cl_2). The asterisk denotes AcOEt.

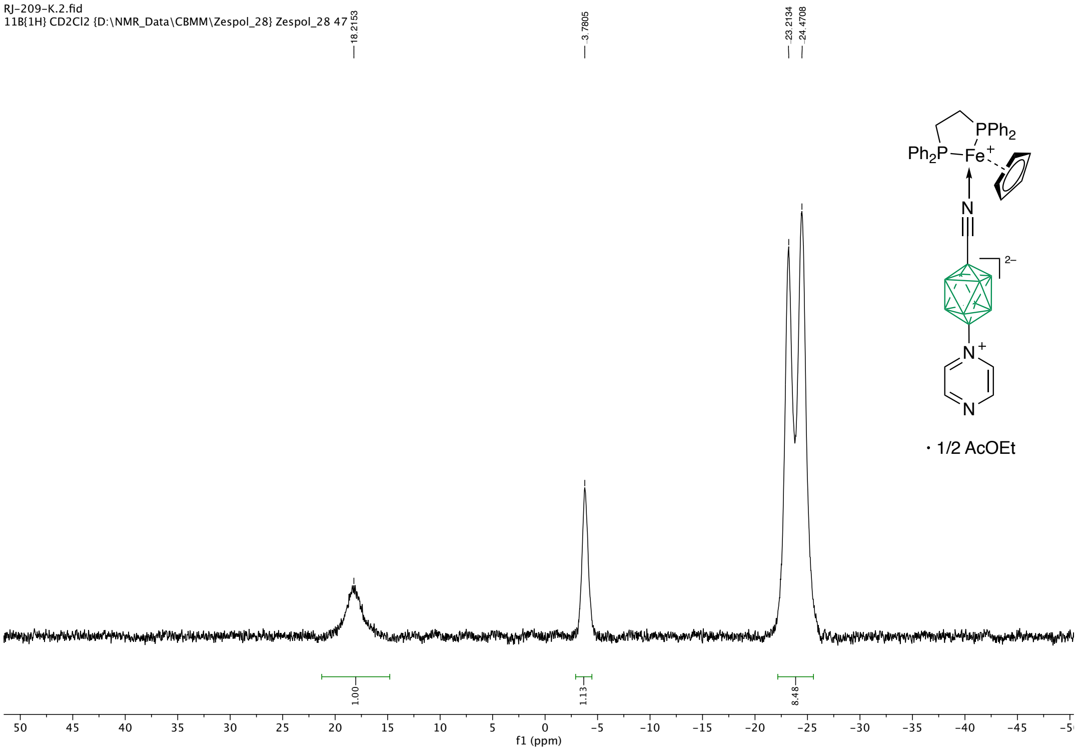

Figure S27: $^{11}B\{^{1}H\}$ NMR spectrum of compound **4d** (128 MHz, CD_2Cl_2).

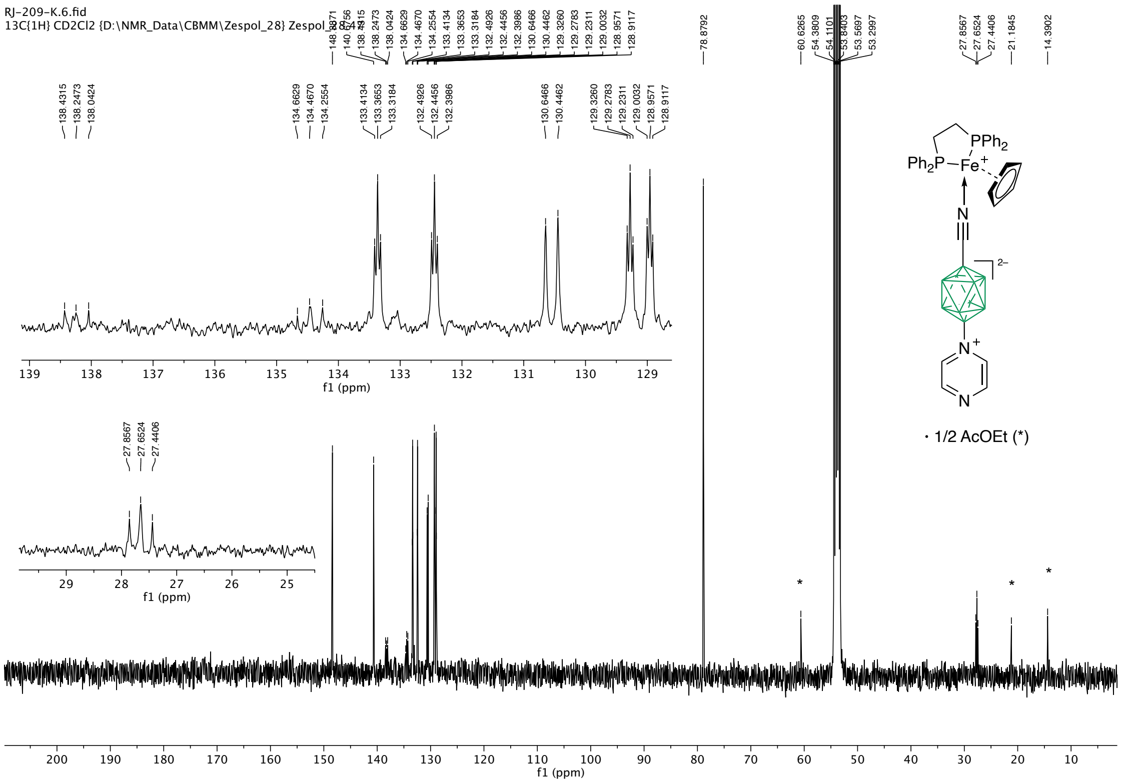

Figure S28: $^{13}C\{^{1}H\}$ NMR spectrum of compound **4d** (101 MHz, CD_2Cl_2).

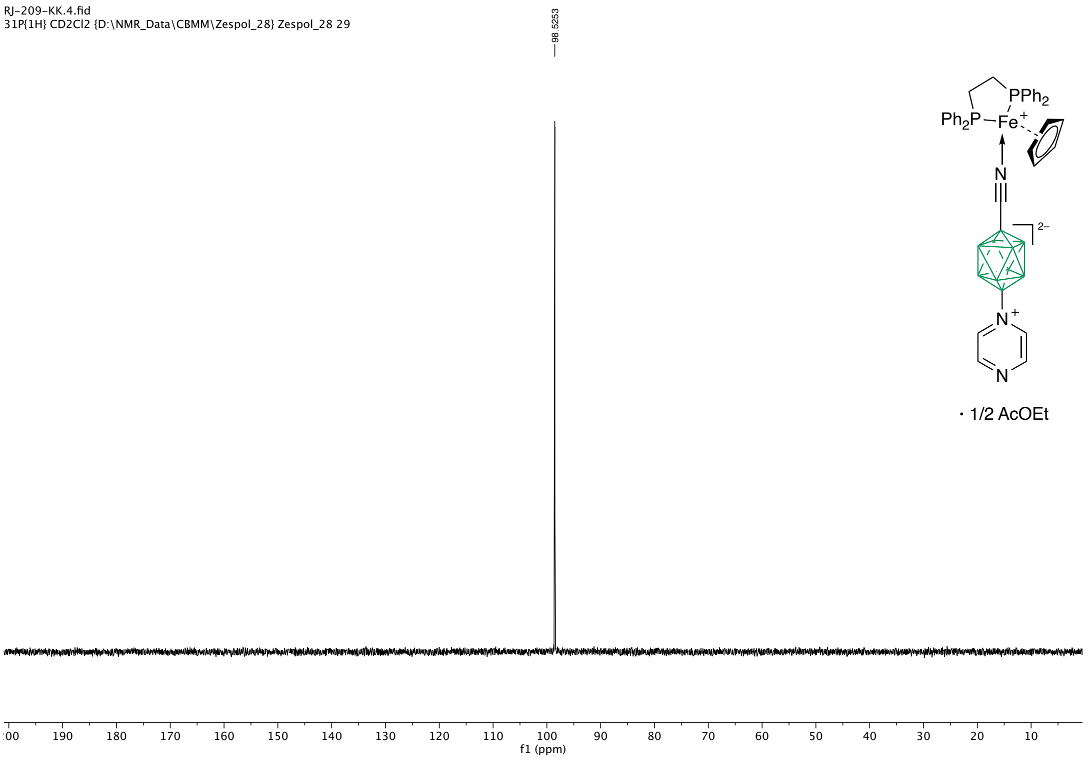

Figure S29: $^{31}P\{^1H\}$ NMR spectrum of compound **4d** (162 MHz, CD_2Cl_2).

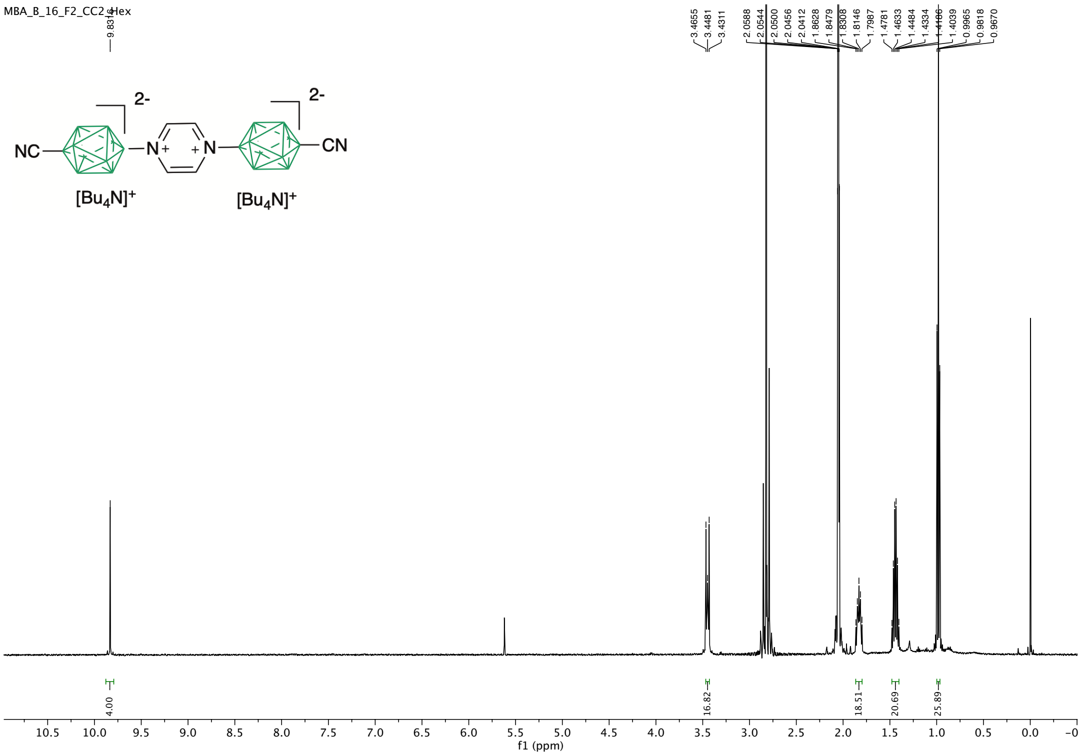

Figure S30: ^1H NMR spectrum of compound **9[Bu$_4$N]** (500 MHz, acetone-d_6).

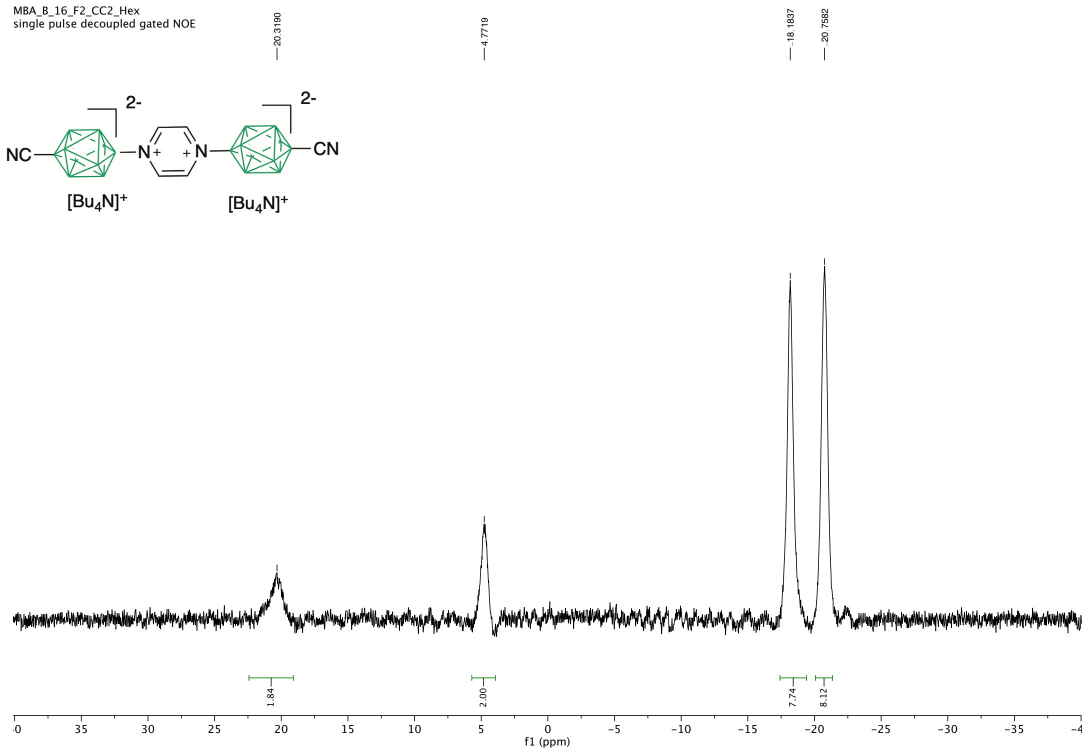

Figure S31: $^{11}B\{^{1}H\}$ NMR spectrum of compound **9[Bu₄N]** (160 MHz, acetone-d_6).

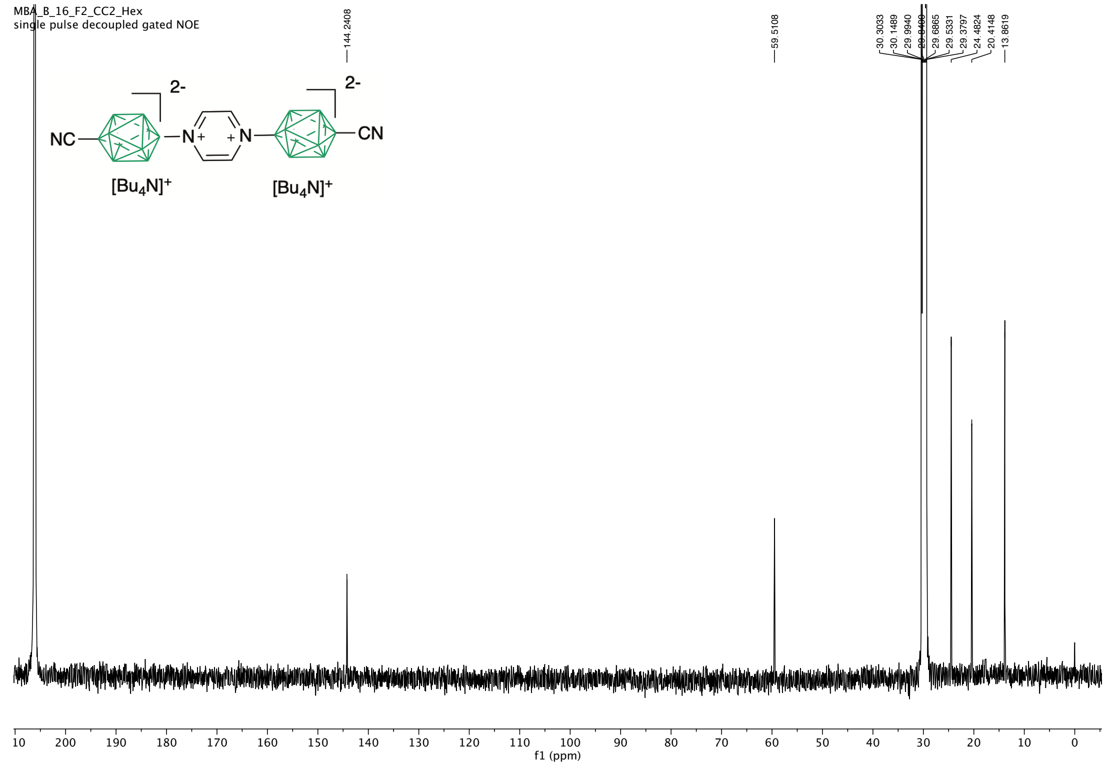

Figure S32: $^{13}C\{^{1}H\}$ NMR spectrum of compound **9[Bu₄N]** (126 MHz, acetone-d_6).

3. XRD data collection and refinement details

a) general comments

All crystal data were collected on a dual source Rigaku SuperNova diffractometer with a Dectris Pilatus3 R 200 K-A detector, equipped with an Oxford Cryosystems Ltd. nitrogen flow apparatus (Cryostream 800 Series) at 100 K using micro-focus X-ray Source Cu K_α radiation, λ = 1.54184 Å. The data were integrated using CrysAlisPro program.[4] Intensities for absorption were corrected using multi-scan or gaussian method as in SCALE3 ABSPACK scaling algorithm implemented in CrysAlisPro program.[4] The crystal data and structure refinement descriptors for all structures are listed in Table S1.

Files CCDC 2348449-2348454, 2352379, 2352380 contain supplementary crystallographic data for this paper. These data can be obtained free of charge from the Cambridge Crystallographic Data Centre via www.ccdc.cam.ac.uk/structures.

b) structure solution and refinement for 2–4

Structures were solved with the ShelXT[5] structure solution program and refined in the ShelXL[6] by the full-matrix least-squares minimization on F^2 using OLEX2 software package.[7] All non-hydrogen atoms were refined anisotropically. All hydrogen atoms were included in idealized positions for structure factor calculations using a riding model.

The asymmetric unite and partial packing diagrams are shown in Figures S33–S42, respectively.

In polymorph B of **4d** two phenyl groups exhibit positional disorder. This was modeled by splitting the six carbon atoms over two positions with the final refined occupancy ratio of 0.64(2):0.36(2) for the phenyl ring attached to P2 and 0.405(11):0.595(11) for the phenyl ring attached to P3. Constraints and restraints such as EADP, SADI, RIGU, and SIMU were used to aid the disorder modeling. Solvent masking was used for partially evaporated and disordered solvent which was not possible to model. A total of 34 electrons were found in a volume of 203 Å^3 in two voids per unit cell. This is consistent with the presence of 0.175[$C_4H_8O_2$] per formula unit, which account for 34 electrons per unit cell.

Table S1. Crystallographic data for selected derivatives.

**Compound**	**2d[Bu₄N]**	**2f[Bu₄N]**	**3c[H]**	**3d[Bu₄N]**	**3f[Bu₄N]**	**4c**	**4d-A**	**4d-B**
CCDC	2348451	2352379	2348449	2348452	2348453	2348454	2348450	2352380
Formula	$C_{20}H_{49}B_{10}N_3$	$C_{20}H_{49}B_{10}N_3$	$C_{11}H_{17}B_{10}N_3$	$C_{21}H_{48}B_{10}N_4$	$C_{21}H_{48}B_{10}N_4$	$C_{42}H_{45}B_{10}FeN_3P_2$	$C_{36}H_{41}B_{10}FeN_3P_2$	$C_{36}H_{41}B_{10}FeN_3P_2$
$D_{calc.}$/ g cm^{-3}	1.041	1.045	1.217	1.073	1.056	1.286	1.305	1.274
m/mm^{-1}	0.394	0.395	0.473	0.421	0.414	3.837	4.236	4.135
Formula Weight	439.72	439.72	299.37	464.73	464.73	817.70	741.61	741.61
Colour	yellow	yellow	orange	yellow	yellow	red	red	red
Shape	plate	plate	plate	plate	plate	plate	plate	plate
T/K	99.9(8)	99.9(8)	100.1(7)	99.9(7)	99.8(7)	100.0(5)	101(2)	100.0(2)
Crystal System	monoclinic	monoclinic	triclinic	monoclinic	monoclinic	monoclinic	triclinic	triclinic
Space Group	$P2_1/n$	$P2_1/n$	*P*-1	$P2_1/n$	$P2_1/c$	$P2_1/n$	*P*-1	*P*-1
a/Å	11.37090(10)	10.37000(10)	8.4666(6)	11.14600(10)	10.28070(10)	12.2661(3)	11.3418(8)	13.2724(2)
b/Å	11.9002(2)	17.1334(2)	8.8274(5)	11.95530(10)	17.7006(2)	24.9758(6)	12.6987(9)	17.0933(3)
c/Å	20.7302(2)	15.8622(2)	12.0274(6)	21.6580(3)	16.0949(2)	27.8967(6)	15.0898(10)	17.8235(2)
a/°	90	90	74.555(5)	90	90	90	109.317(6)	78.2530(10)
β/°	91.0870(10)	97.1840(10)	71.700(5)	94.8810(10)	93.8140(10)	98.875(2)	100.272(6)	89.6340(10)
γ/°	90	90	79.062(5)	90	90	90	105.707(6)	77.7070(10)
V/Å^3	2804.62(6)	2796.17(6)	817.07(9)	2875.54(5)	2922.38(6)	8444.0(3)	1886.6(2)	3865.25(10)
Z	4	4	2	4	4	8	2	4
Z'	1	1	1	1	1	2	1	2
Q_{min}/°	4.266	3.814	3.970	4.097	3.716	2.387	3.248	2.534
Q_{max}/°	78.953	76.667	76.364	78.834	79.083	76.961	66.605	76.733
Measured Refl.	36832	15498	6954	27942	29244	48668	14020	43774
Independent Refl.	5976	5672	3287	6071	6219	17241	6426	15797
Reflections with I > 2(I)	5432	5301	2569	5252	5659	13656	5029	13408
R_{int}	0.0487	0.0207	0.0346	0.0430	0.0373	0.0762	0.0527	0.0433
Parameters	306	306	217	320	320	1045	469	1041
Restraints	0	0	0	0	0	0	0	546
Largest Peak	0.479	0.261	0.372	0.364	0.425	1.179	1.759	0.641
Deepest Hole	-0.318	-0.193	-0.203	-0.190	-0.464	-0.782	-0.501	-0.862
GooF	1.045	1.038	1.023	1.043	1.068	1.018	1.105	1.067
wR_2 (all data)	0.1425	0.1130	0.1791	0.1504	0.1464	0.1772	0.2294	0.1136
wR_2	0.1394	0.1114	0.1658	0.1455	0.1428	0.1653	0.2173	0.1087
R_1 (all data)	0.0554	0.0429	0.0760	0.0624	0.0559	0.0803	0.1051	0.0520
R_1	0.0516	0.0409	0.0611	0.0549	0.0522	0.0643	0.0852	0.0429

Molecules of **2d[Bu$_4$N]** form undulated ribbons of discrete dimers with the separation between their molecular axes of 5.50 and 5.66 Å (Figure S33) and close C–H···B (2.918-3.098 Å) intermolecular contacts between the {*closo*-B$_{10}$} cages and the pyrazine rings (Figure S34). The neighboring ribbons are isolated by layers of [Bu$_4$N]$^+$ cations and are rotated by 75.9° relative to each other.

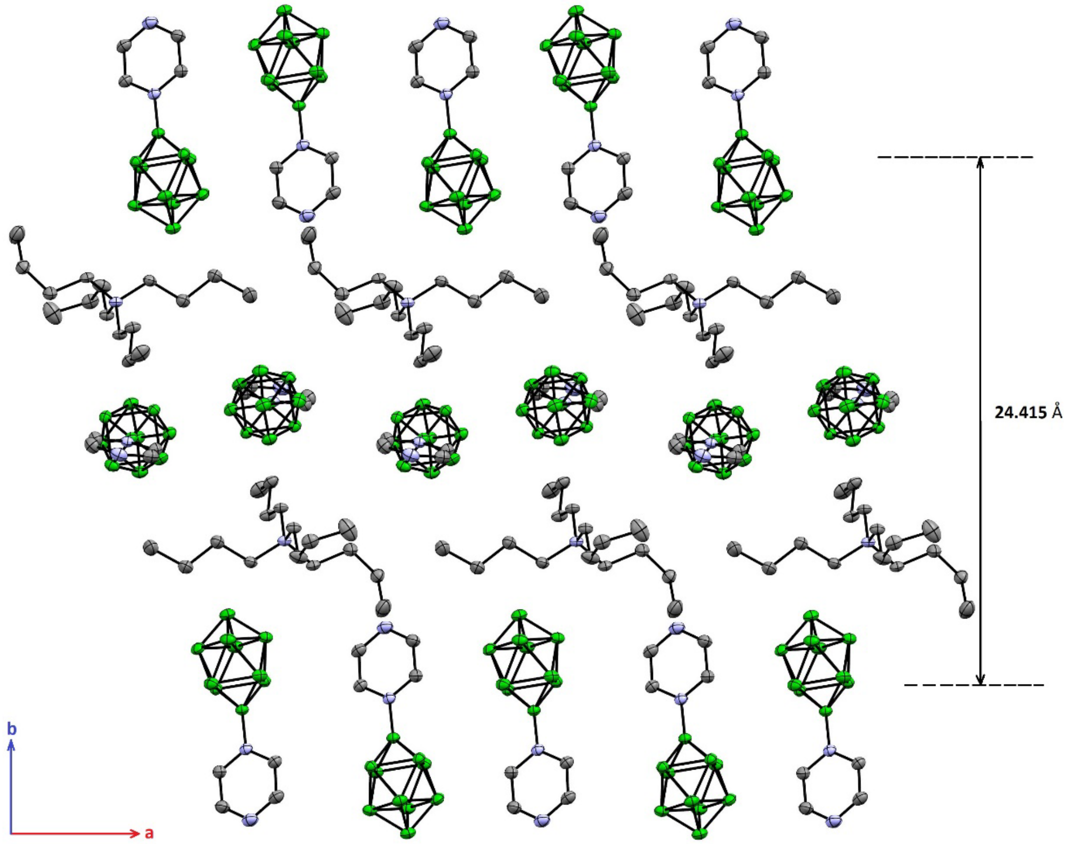

Figure 33. Partial packing diagram for **2d[Bu$_4$N]** in the (0-11) plane. Hydrogen atoms are omitted for clarity.

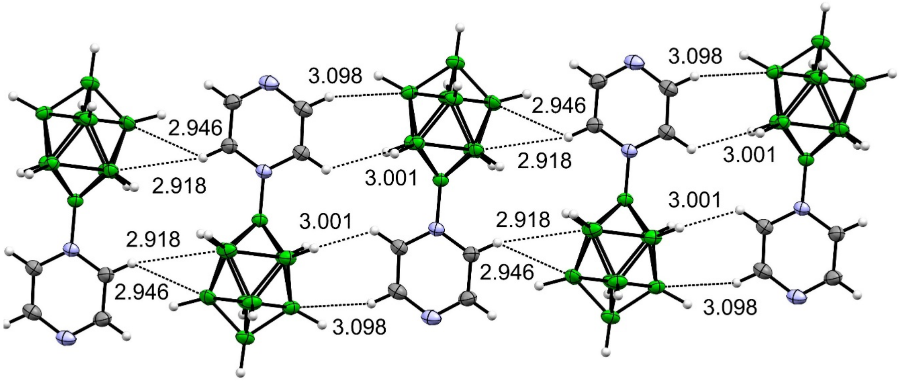

Figure S34. Partial packing diagram for **2d[Bu$_4$N]** in the (0-11) plane. Counterions are omitted for clarity. Dotted lines indicate short intermolecular contacts.

Molecules of **2f[Bu$_4$N]** display zigzag chains formed by C–H···B (2.643–2.795 Å) interactions between the {*closo*-B_{10}} cages and pyridazine rings along the (-111) plane (Figure S35), which are isolated by surrounding $[Bu_4N]^+$ counterions (Figure S36).

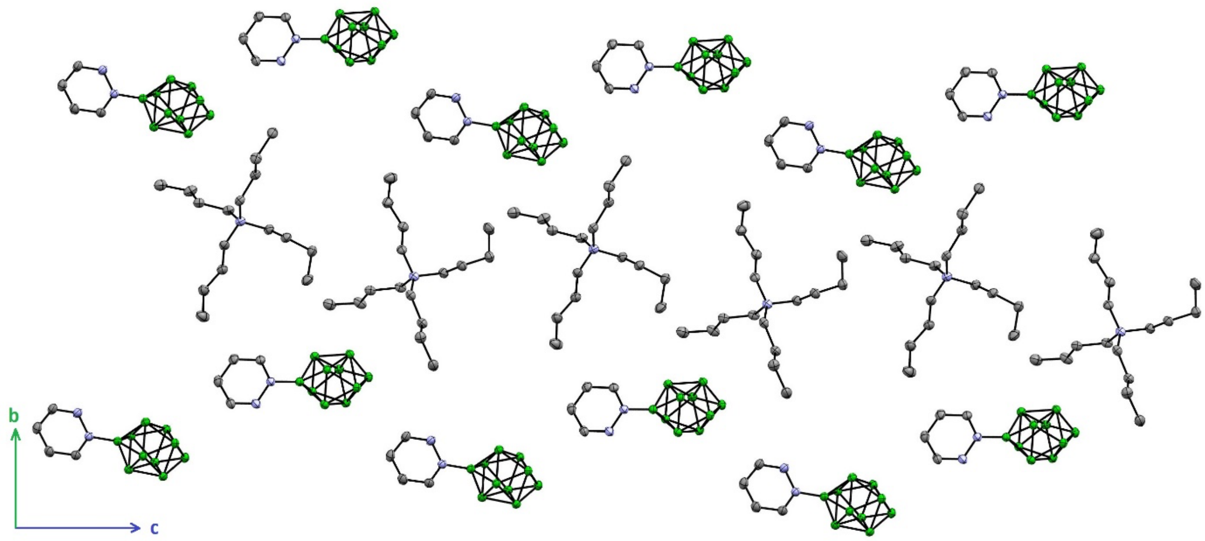

Figure S35. Partial packing diagram for **2f[Bu$_4$N]** along the (-111) plane. Hydrogen atoms are omitted for clarity.

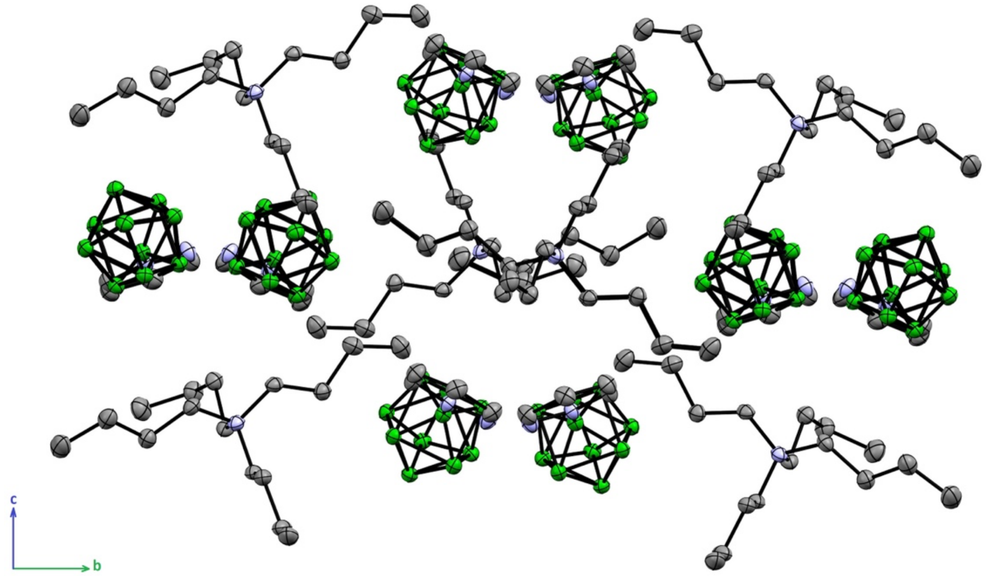

Figure S36. Partial packing diagram for **2f[Bu$_4$N]**. Hydrogen atoms are omitted for clarity.

Molecules of **3c[H]** form infinite chains running along the [100] direction through N···H close interactions (1.883 Å) between neighboring molecules (Figure S37). The chains are separated by 5.59 Å in the (010) plane (Figure S37) and interact with chains in other planes through short B–H···C (2.743 Å), C–H···B (3.091 Å) and B–H···N (2.708 Å)

contacts between the {*closo*-B_{10}} cages and pyridine rings. Thus, forming adjacent sheets that are linked between the {*closo*-B_{10}} cages and pyridine rings along *b*-axis (Figure S38).

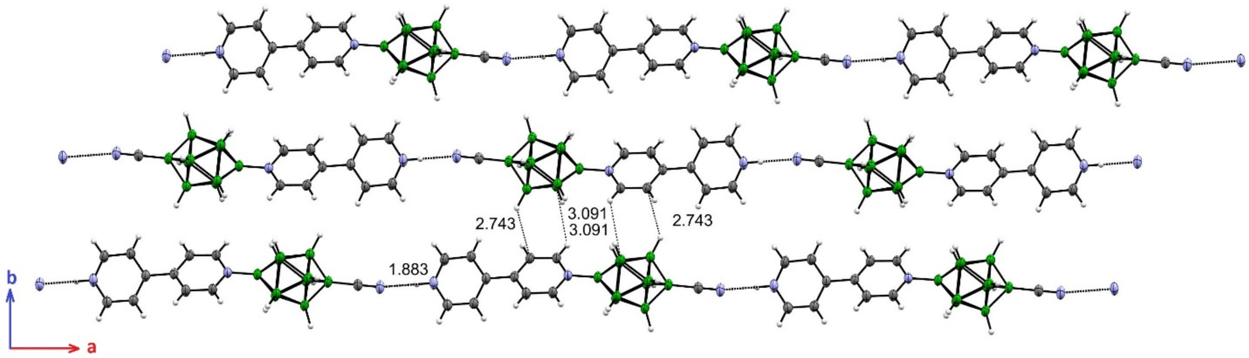

Figure S37. Partial packing diagram for **3c[H]** in the (010) plane. The dotted lines show short N···H contacts.

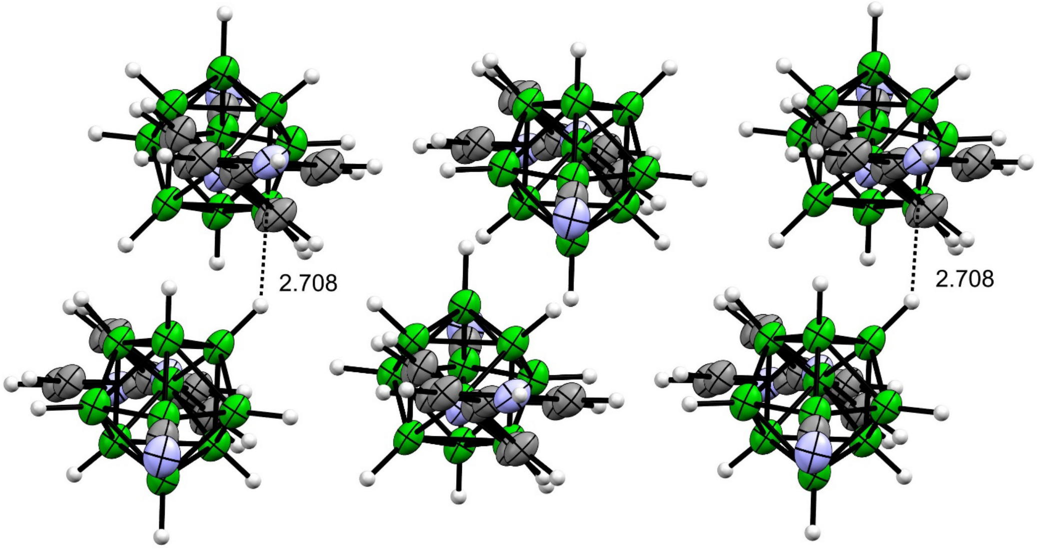

Figure 38. Partial packing diagram for **3c[H]** in the (001) plane. The dotted lines show short B–H···N intermolecular contacts.

Crystal packing of molecules of **3d[Bu_4N]** is similar to that of **2d[Bu_4N]**. Molecules of **3d[Bu_4N]** form undulated ribbons of discrete dimers with the separation between their molecular axes of 5.44 and 5.505 Å (Figure S39) and close C–H···B (2.964-3.041 Å) contacts between the {*closo*-B_{10}} cages and the pyrazine rings (Figure S40). The neighboring ribbons are isolated by a layer of $[Bu_4N]^+$ and are rotated by 73.9^o relative to each other. The main difference between the structure of **3d[Bu_4N]** and **2d[Bu_4N]** is the smaller separation between the ribbons (22.066 Å *vs* 24.415 Å).

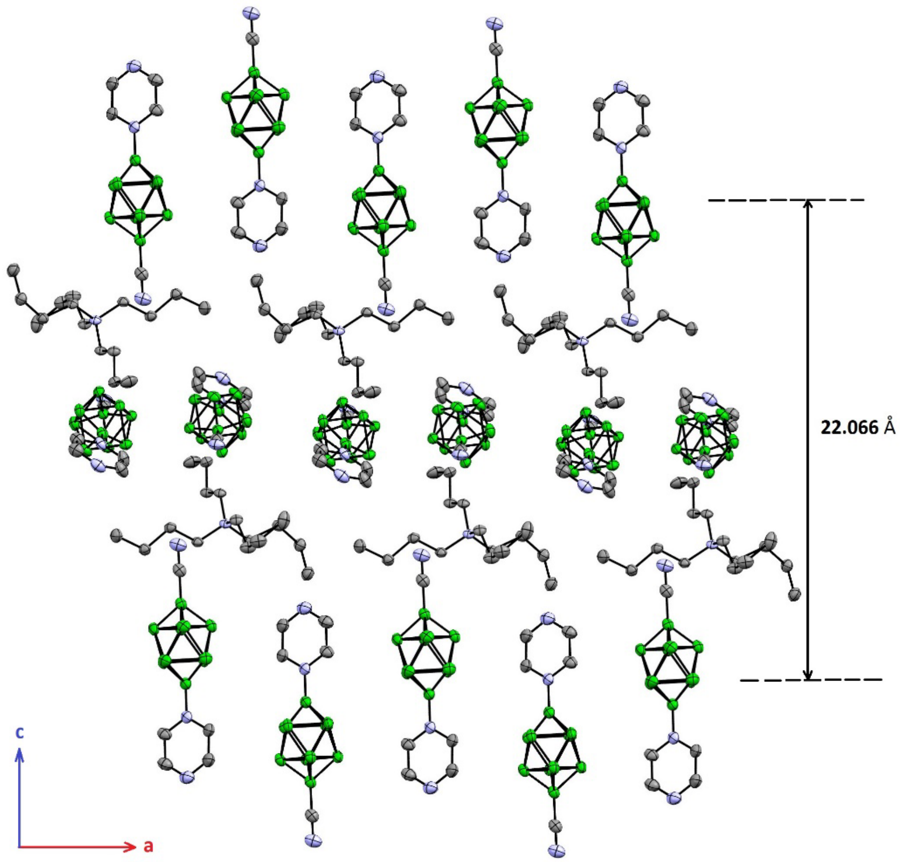

Figure S39. Partial packing diagram for **3d[Bu$_4$N]** in the (011) plane. Hydrogen atoms are omitted for clarity.

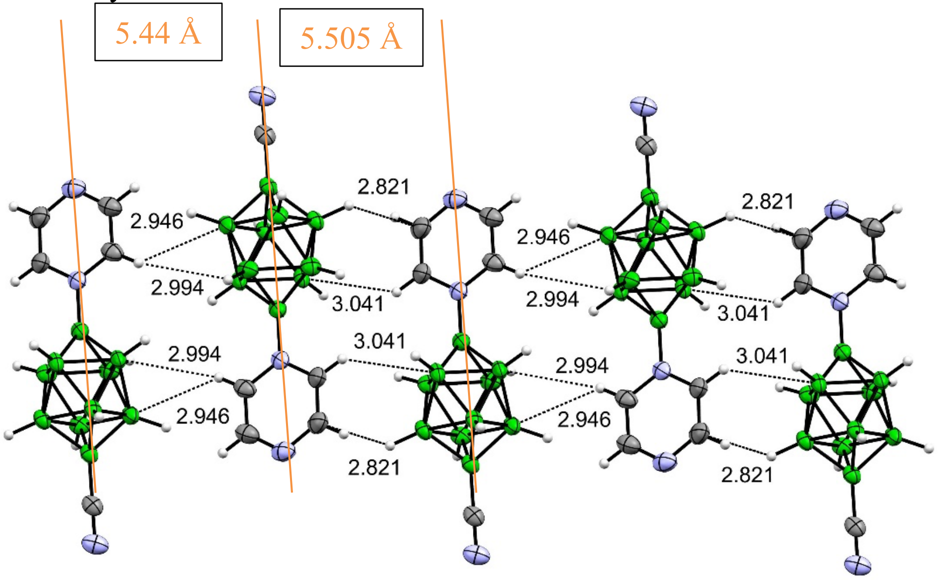

Figure S40. Partial packing diagram for **3d[Bu$_4$N]** in the (011) plane. Counterions are omitted for clarity. Short intermolecular contacts are depicted as dotted lines.

Molecules of **3f[Bu$_4$N]** form discrete antiparallel dimers through C–H···B interactions between the {*closo*-B_{10}} cages and pyridazine rings (2.810–2.938 Å, Figure

S41). Axes of molecules forming the dimer are separated by 5.45 Å, while axis of the neighboring dimers form an angle of 16.75°. Supramolecular layers in **3f[Bu$_4$N]** extend along the (100) plane.

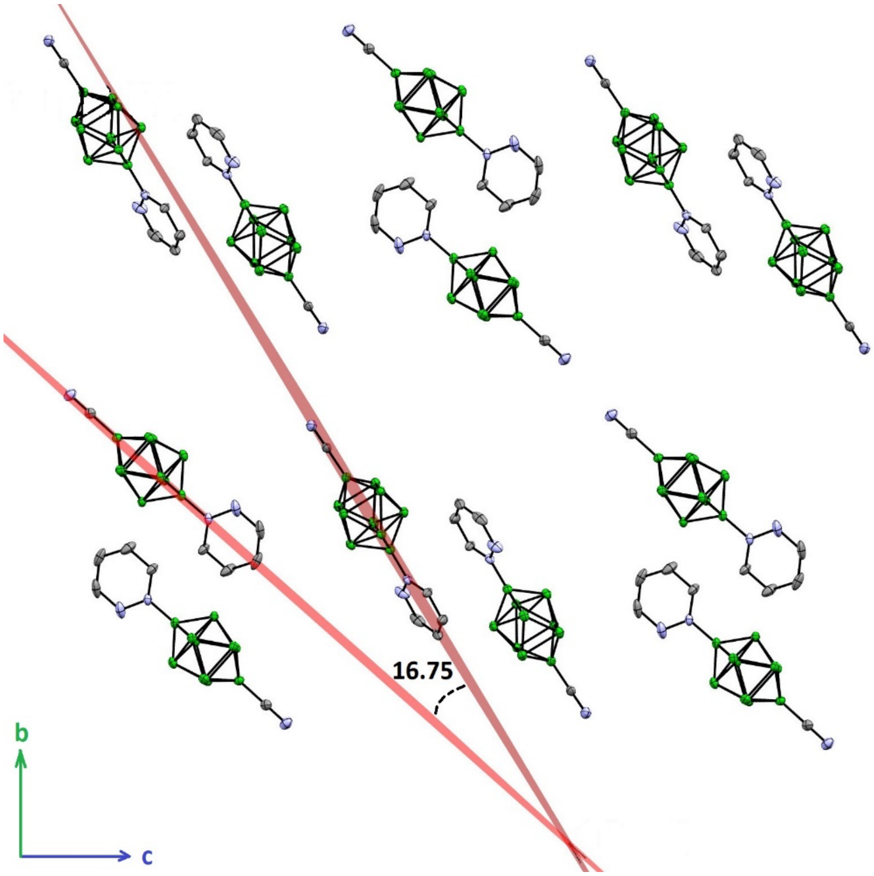

Figure S41. Partial packing diagram for **3f[Bu$_4$N]** in the (100) plane. Hydrogen atoms and counterions are omitted for clarity.

The two unique molecules of Fe complexes **4c** and **4d** are shown in Figure S42 and S43, respectively (Figures S42 and S43). In the latter, the Ph ring atoms of the minor positional disorder were removed. Also, the Cp ring interacts with the pyrazine ring with the distance between centroids of 3.36 Å (Figure S43).

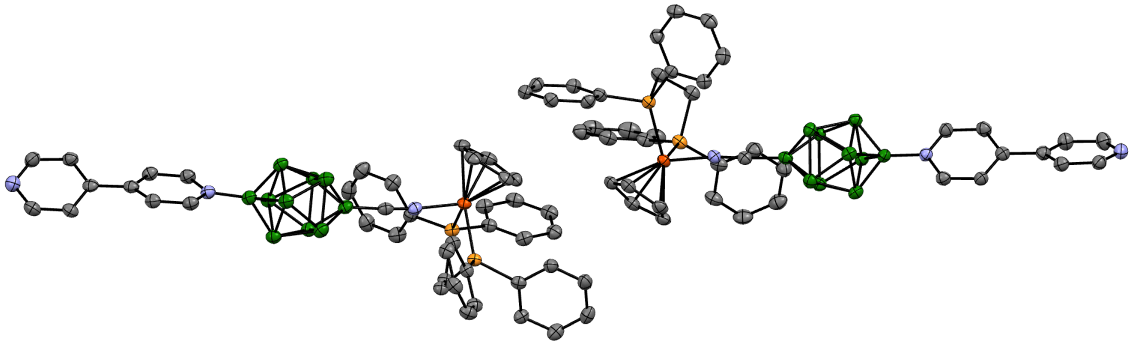

Figure S42. Partial packing diagram for **4c** showing two unique molecules A and B. Hydrogen atoms are omitted for clarity.

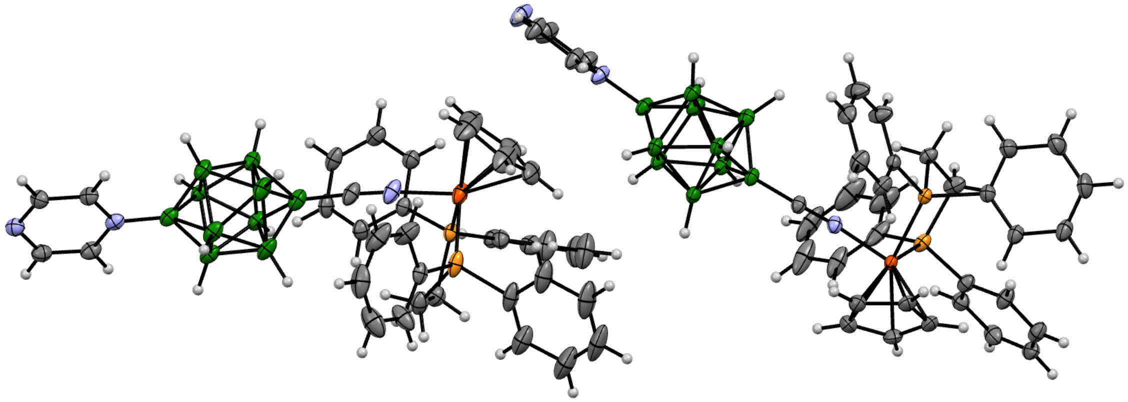

Figure S42. Partial packing diagram for **4d** showing two unique molecules A and B. Positionally disordered atoms are omitted for clarity.

c) attempted preparation of crystals of 5c

Upon mixing of warm methanolic solutions of **4c** and (pdc)Cu(aq)$_3$, fine needless were formed within 10-15 min (Figure S43). To slow down the crystallization process the solutions were diluted by ten-fold and carefully layered in an NMR tube. The result was the same even though the crystals were growing overnight. Hydrothermal growth of crystals at 80 ^{o}C for 24 h followed by slow cooling at a rate of 3 K/h gave red conglomerates. Addition of some H_2O and repeating the process did not improve the quality of the crystals.

Recrystallization of the obtained solid material was attempted by very slow evaporation of a mixture of MeCN/MeOH and MeCN/EtOH over several days. Solid was dissolved in warm MeCN (not boiling) and alcohol was added. In the first case soft feather-like crystals were obtained, while in the MeCN/EtOH thin needless were obtained again.

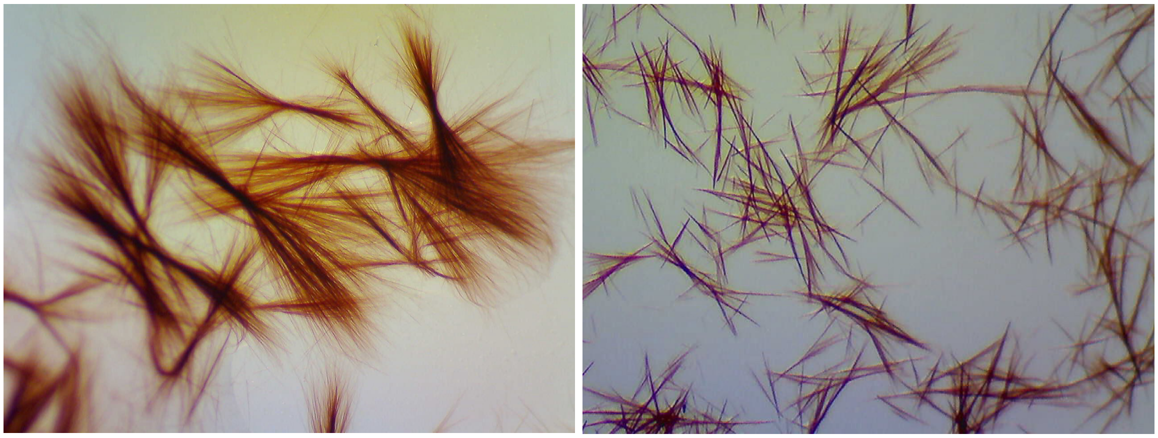

Figure S43. Thin needles of **5c** obtained by recrystallization.

At least 10 specimens were mounted on the diffractometer and the best experiment is described below.

Experimental details of the XRD measurement. Single red needle-shaped crystals of **5c** were isolated from MeOH. A suitable crystal 0.17×0.04×0.03 mm^3 was selected and mounted on a suitable support on a SuperNova, Dual, Cu at home/near, Pilatus 200K diffractometer. The crystal was kept at a steady T = 99.8(8) K during data collection. The structure was solved with the ShelXT 2014/5 structure solution program using the Intrinsic Phasing solution method and by using Olex2[7] as the graphical interface. The model was refined with version 2018/1 of ShelXL 2018/1[6] using Least Squares minimization.

Crystal Data for **5c**. $C_{49}H_{50}B_{10}CuFeN_4O_5P_2$, M_r = 1064.36, tetragonal, I-4 (No. 82), a = 36.821(3) Å, b = 36.821(3) Å, c = 8.3129(10) Å, $\alpha = \beta = \gamma = 90^\circ$, V = 11271(2) Å^3, T = 99.8(8) K, Z = 8, Z' = 1, μ(Cu K$_\alpha$) = 3.428, 14957 reflections measured, 8911 unique (R_{int} = 0.1410), which were used in all calculations. The final wR_2 was 0.4183 (all data) and R_1 was 0.1386 (I > 2(I)).

These data are not deposited with CCDC.

4. Electronic spectroscopy and emission spectra

Electronic absorption spectra were measured for three concentrations in spectrophotometric grade MeCN and molar extinction coefficients ε were obtained from the Beer's Law plot with typical r^2 of >0.995 for each compound. Fluorescence spectra were recorded for diluted MeCN solutions using a Hitachi F-4500 Fluorescence Spectrophotometer. The excitation wavelength was set to the absorbance max in MeCN and λ_{em} was scanned from λ_{max} +50 nm to 900 nm. UV spectra, Beer's Law plots and emission spectra are shown in Figures S44–S53.

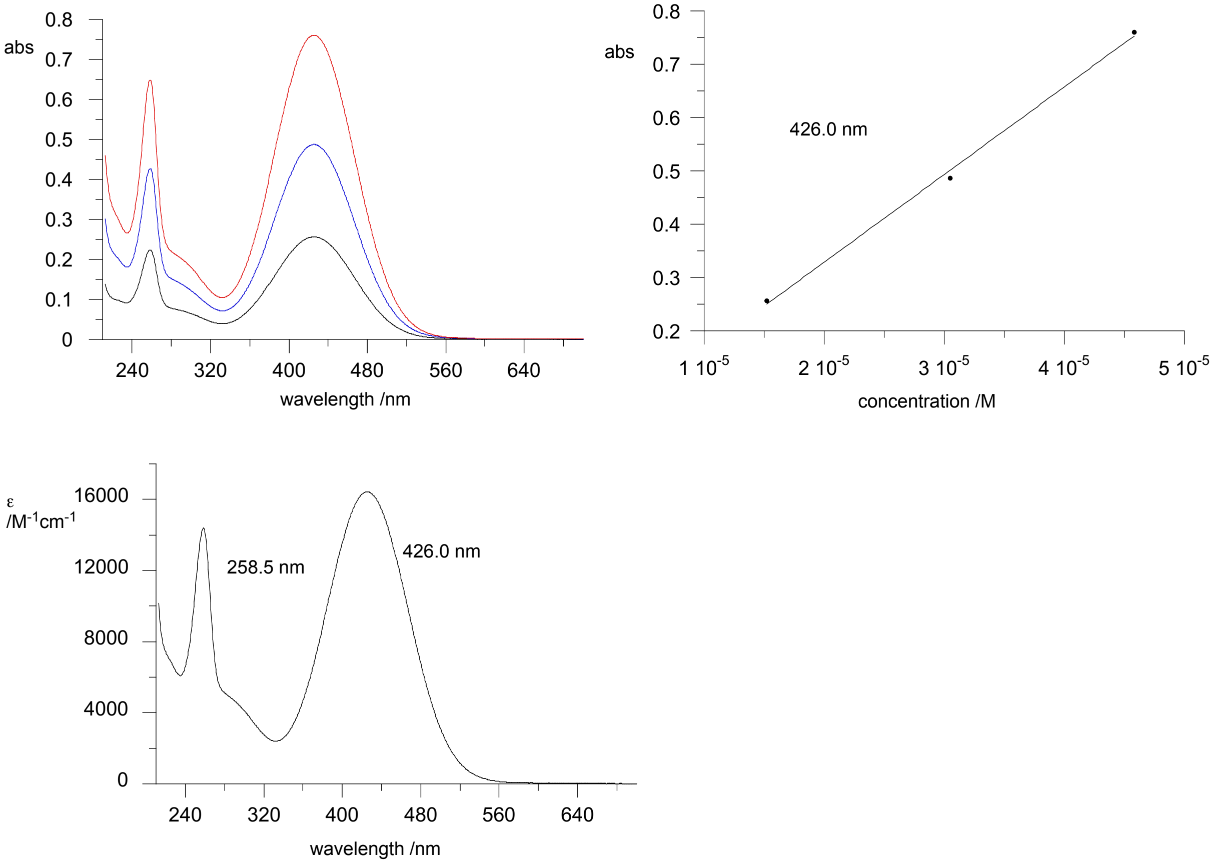

Figure S44. Clockwise: electronic absorption spectra for **2d[Bu$_4$N]** in CH_3CN for 3 concentrations; determination of molar extinction coefficient ε at λ = 426.0 nm (best fit function: ε = 16434(215)×conc, r^2 = 0.9976); molar extinction.

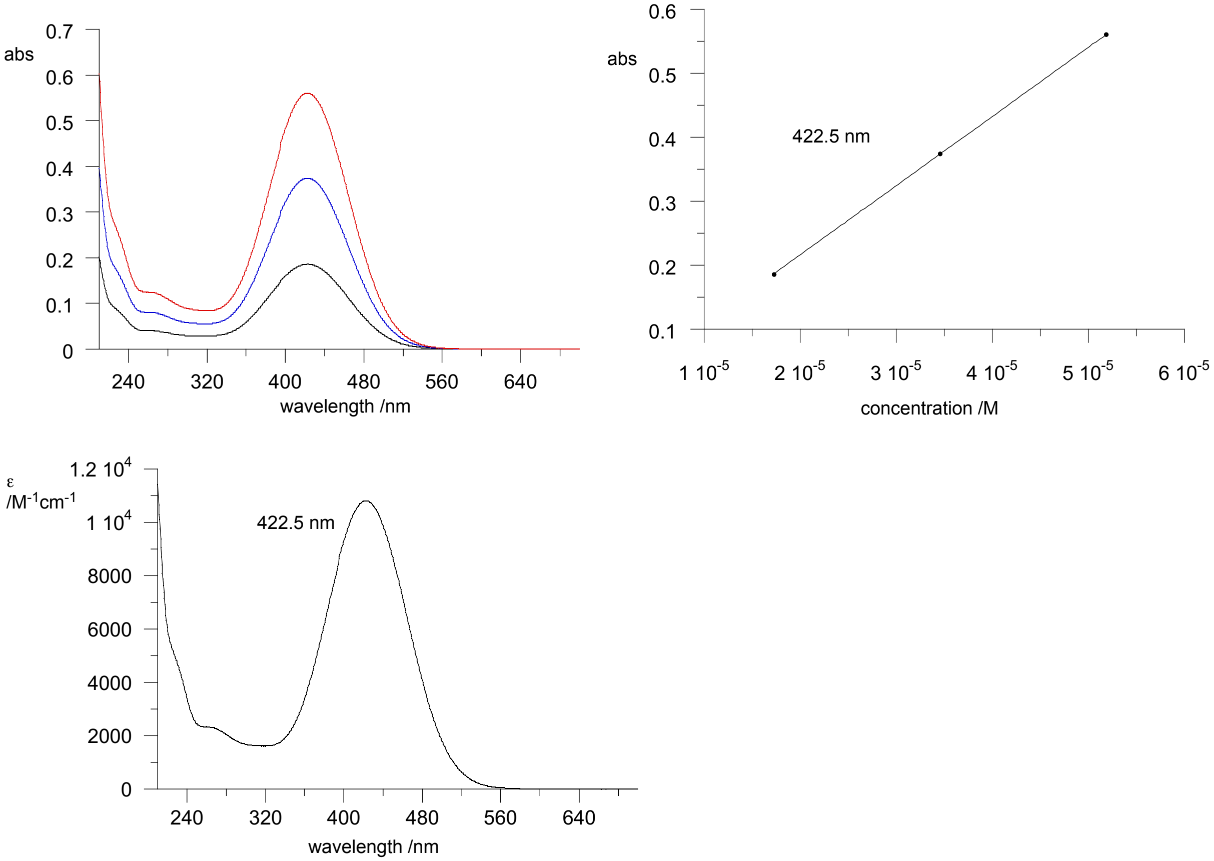

Figure S45. Clockwise: electronic absorption spectra for **2f[Bu$_4$N]** in CH_3CN for 3 concentrations; determination of molar extinction coefficient ε at λ = 422.5 nm (best fit function: ε = 10808(12)×conc, r^2 = 0.9999); molar extinction.

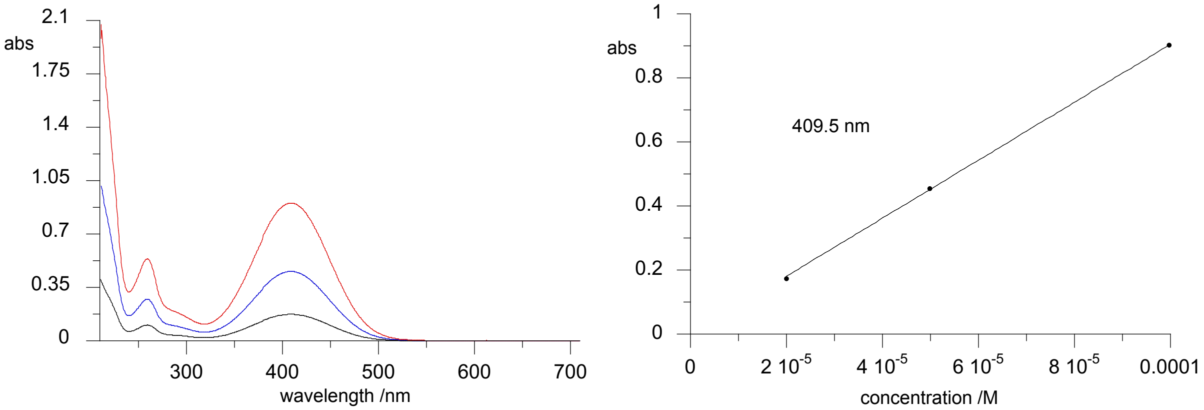

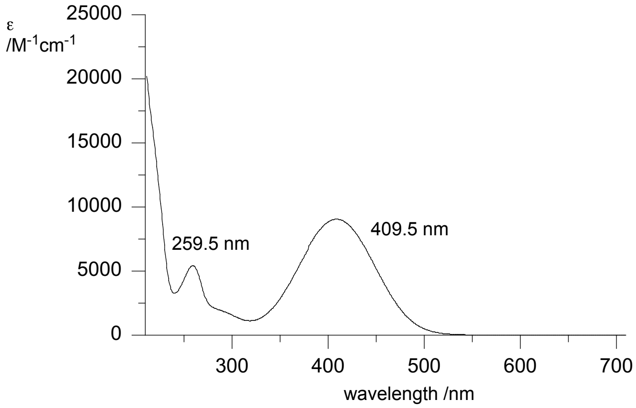

Figure S46. Clockwise: electronic absorption spectra for **3b[Bu$_4$N]** in CH_3CN for 3 concentrations; determination of molar extinction coefficient ε at λ = 409.5 nm (best fit function: ε = 9050(49)×conc, r^2 = 0.9998); molar extinction.

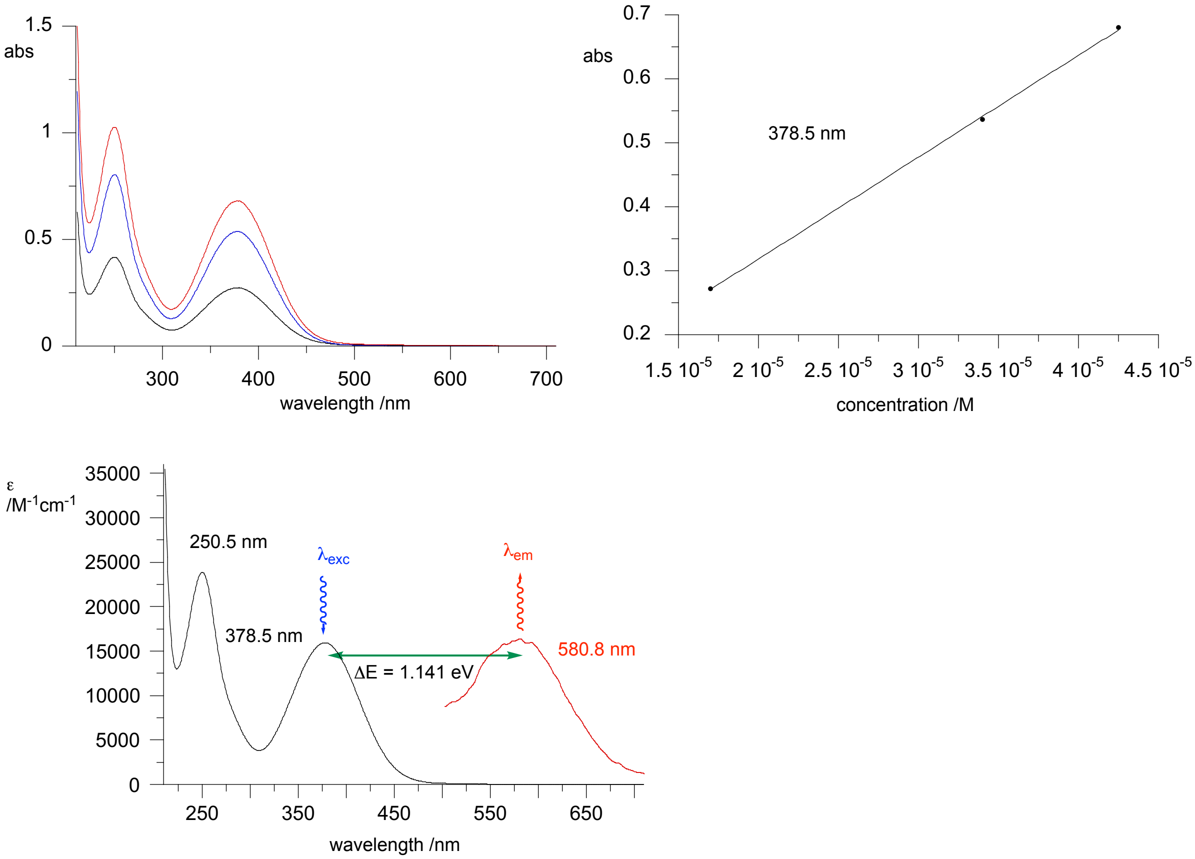

Figure S47. Clockwise: electronic absorption spectra for **3c[Bu$_4$N]** in CH_3CN for 3 concentrations; determination of molar extinction coefficient ε at λ = 378.5 nm (best fit function: ε = 15917(74)×conc, r^2 = 0.9996); molar extinction and normalized fluorescence spectra with indicated excitation wavelength and Stokes shift.

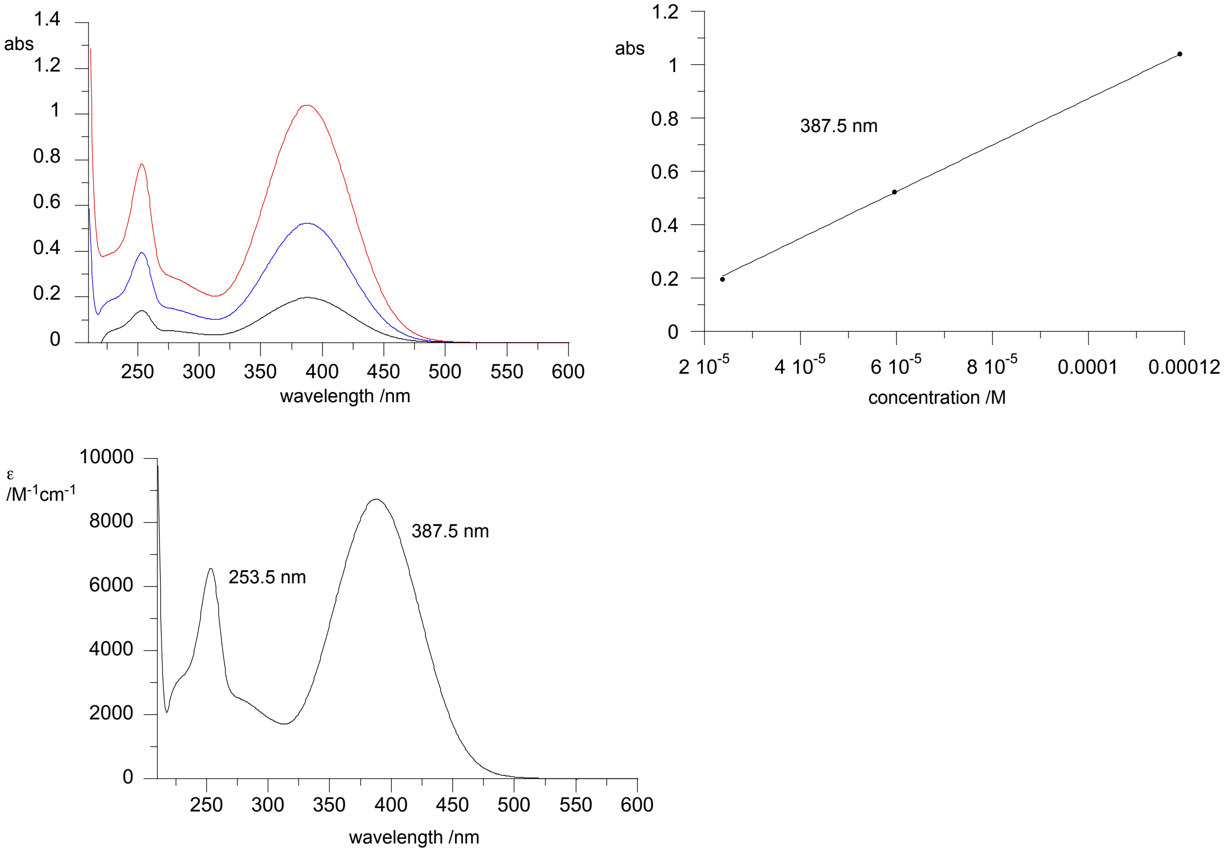

Figure S48. Clockwise: electronic absorption spectra for **3d[Bu$_4$N]** in CH_3CN for 3 concentrations; determination of molar extinction coefficient ε at λ = 387.5 nm (best fit function: ε = 8729(61)×conc, r^2 = 0.9996); molar extinction.

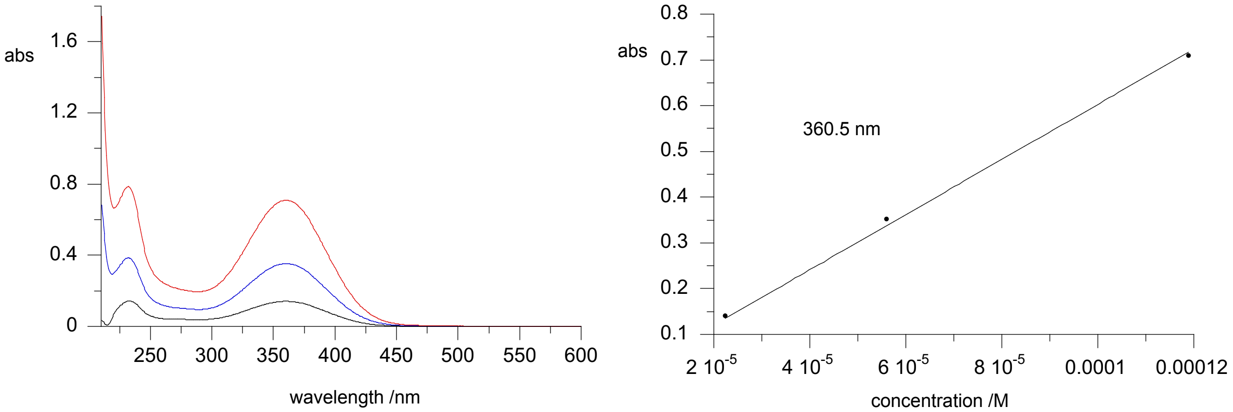

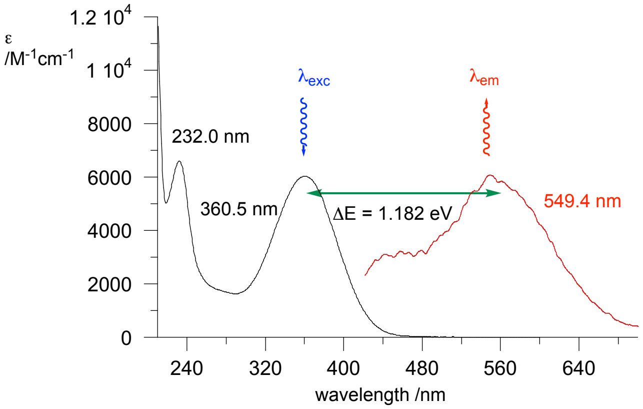

Figure S49. Clockwise: electronic absorption spectra for **3e[Bu$_4$N]** in CH_3CN for 3 concentrations; determination of molar extinction coefficient ε at λ = 360.0 nm (best fit function: ε = 6030(97)×conc, r^2 = 0.998); molar extinction and normalized fluorescence spectra with indicated excitation wavelength and Stokes shift.

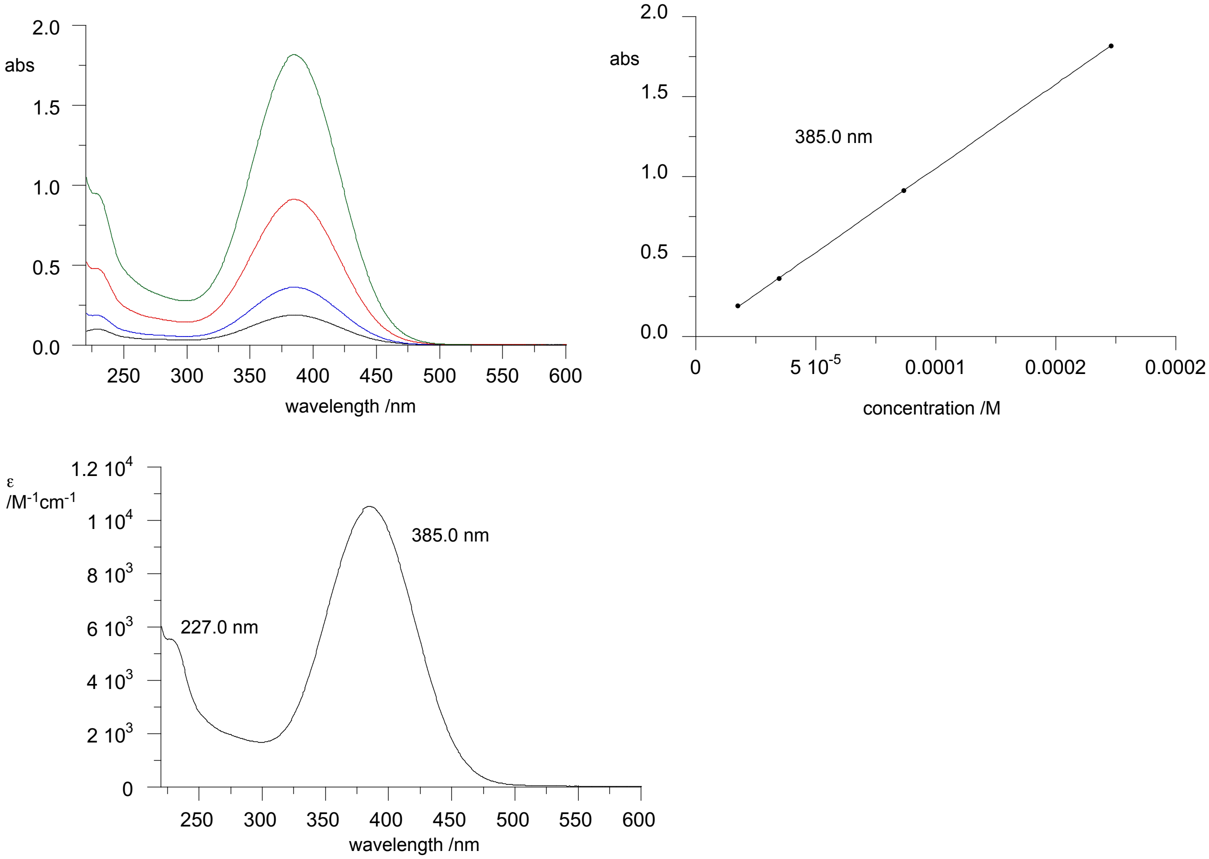

Figure S50. Clockwise: electronic absorption spectra for **3f[Bu$_4$N]** in CH_3CN for 4 concentrations; determination of molar extinction coefficient ε at λ = 385.0 nm (best fit function: ε = 10506(16)×conc, r^2 = 0.999); molar extinction.

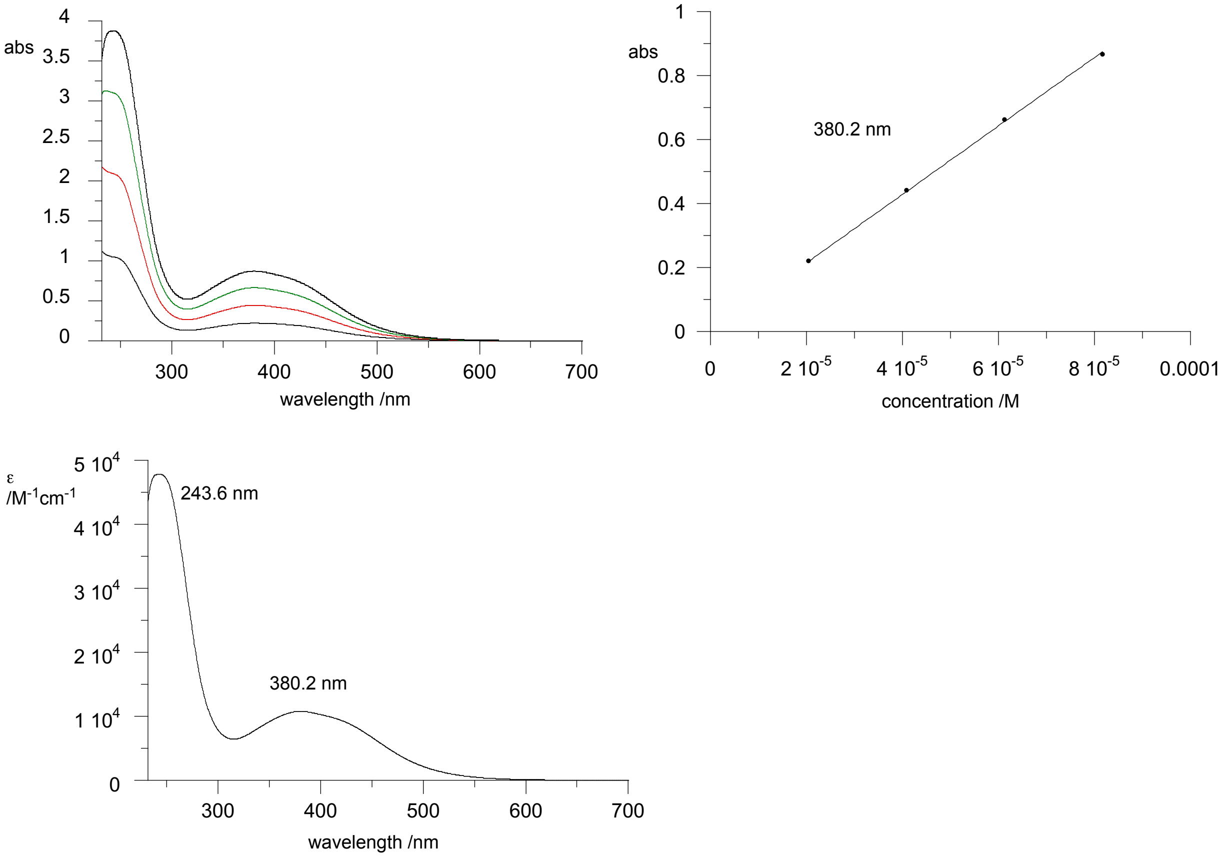

Figure S51. Clockwise: electronic absorption spectra for **4c** in CH_2Cl_2 for 4 concentrations; determination of molar extinction coefficient ε at λ = 380.2 nm (best fit function: ε = 10706(52)×conc, r^2 = 0.9996); molar extinction.

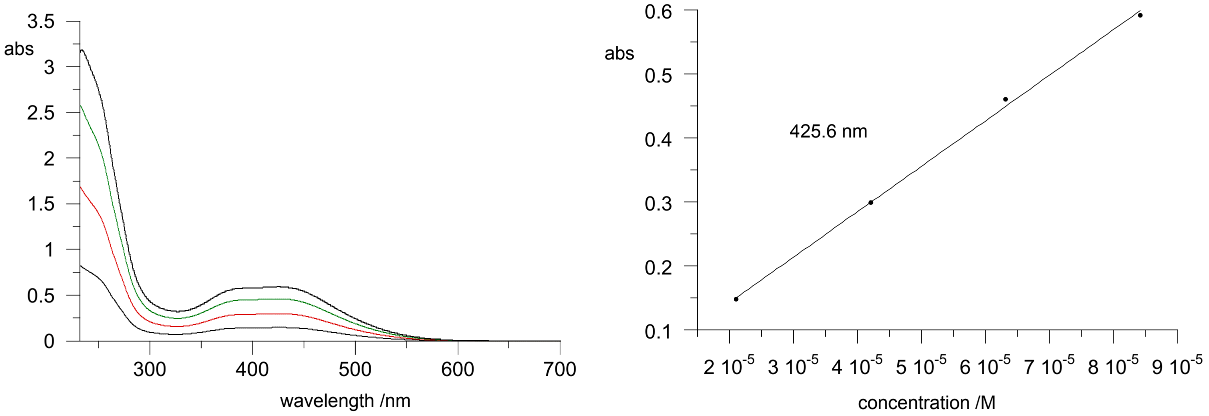

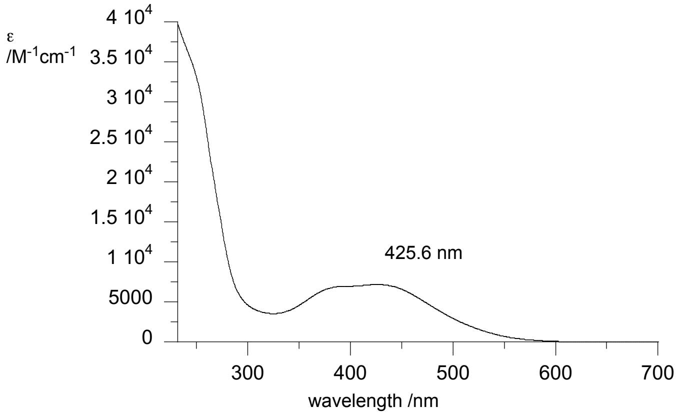

Figure S52. Clockwise: electronic absorption spectra for **4d** in CH_2Cl_2 for 4 concentrations; determination of molar extinction coefficient ε at λ = 425.6 nm (best fit function: ε = 7113(65)×conc, r^2 = 0.998); molar extinction.

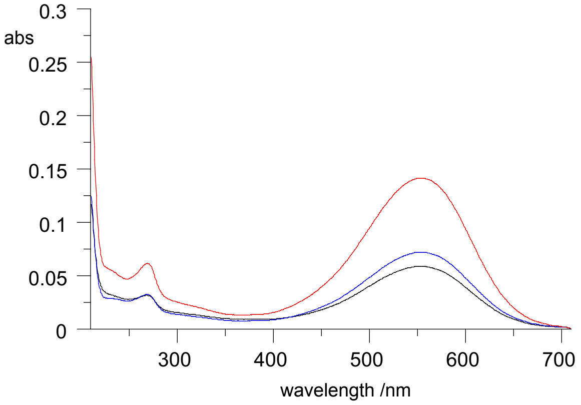

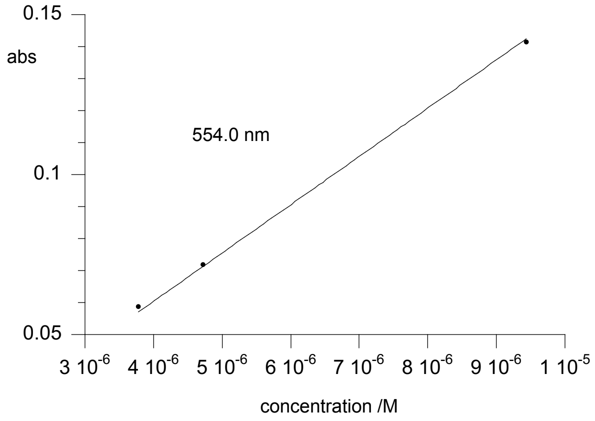

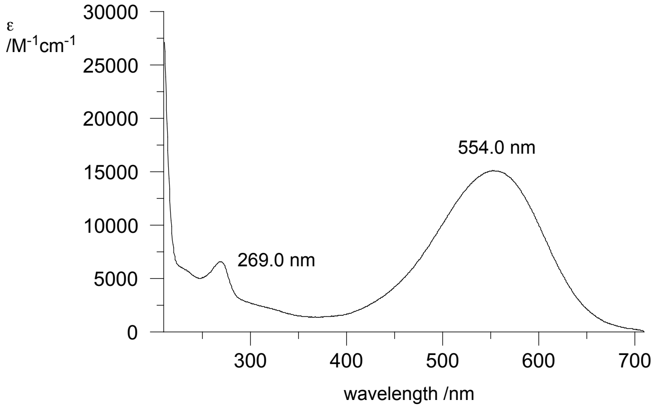

Figure S53. Clockwise: electronic absorption spectra for **9[Bu₄N]** in CH_3CN for 3 concentrations; determination of molar extinction coefficient ε at λ = 554.0 nm (best fit function: ε = 15096(134)×conc, r^2 = 0.9999); molar extinction.

5. Electrochemical data

Electrochemical analysis was conducted using a Metrohm Autolab potentiostat. Cyclovoltammetric (CV) measurements for **4** were performed in degassed solutions of 0.5 mM analyte in 100 mM $[Bu_4N]^+[PF_6]^-$ in CH_2Cl_2 as the supporting electrolyte. The voltammograms were recorded at ca 22 °C using a glassy carbon working electrode (φ = 1 mm), a Pt-wire counter electrode and an Ag/AgCl wire as pseudo-reference electrode at a scan rate of 50 mV s^{-1} and typical range -0.5 to +1 V. The potentials are referenced to the Fc/Fc^+ couple in separate measurements after addition of internal reference to the analyte solutions (decamethylferrocene ($Me_{10}Fc$) ($Me_{10}Fc$/ $Me_{10}Fc^+$ vs Fc/Fc^+ is –0.546 V, Figures S54 and S55).

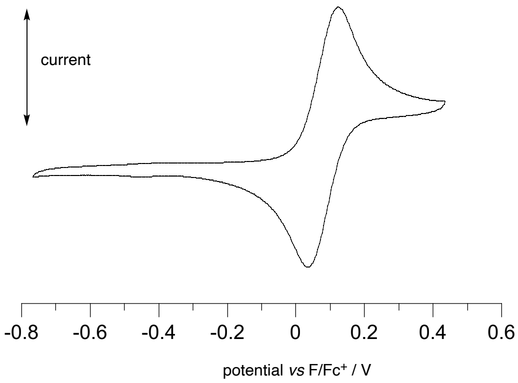

Figure S54. Cyclic voltammogram (CV) of **4c** in CH_2Cl_2 referenced to the Fc/Fc^+ couple. From CV: $E_{1/2}$ = 0.080 V.

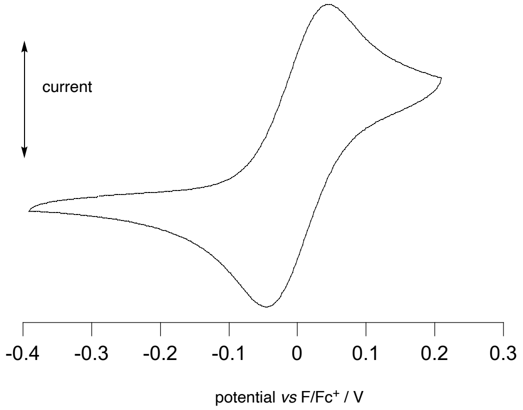

Figure S55. Cyclic voltammogram (CV) of **4d** in CH_2Cl_2 referenced to the Fc/Fc^+ couple. From CV: $E_{1/2}$ = 0.101 V.

6. Partial output data for TD-DFT calculations

```
CAM-B3LYP/Def2TZVP TD(NStates=20) SCRF(solvent=CH3CN)
```

2a

```
Excited State   1:      Singlet-A1     3.9675 eV  312.50 nm  f=0.2908
<S**2>=0.000
      47 -> 52         0.11400
      51 -> 52         0.69074
 This state for optimization and/or second-order correction.
 Total Energy, E(TD-HF/TD-KS) =  -502.183485410
```

2b

```
Excited State   1:      Singlet-A1     3.1771 eV  390.24 nm  f=0.4174
<S**2>=0.000
      53 -> 58         0.10095
      57 -> 58         0.69259
 This state for optimization and/or second-order correction.
 Total Energy, E(TD-HF/TD-DFT) =  -594.443340416
```

2c

```
Excited State   1:      Singlet-A      3.5870 eV  345.65 nm  f=0.4998
<S**2>=0.000
      67 -> 72         0.11171
      71 -> 72         0.67966
      71 -> 75         0.12865
 This state for optimization and/or second-order correction.
 Total Energy, E(TD-HF/TD-DFT) =  -749.248918976
```

2d

```
Excited State   1:      Singlet-A1     3.3933 eV  365.38 nm  f=0.3178
<S**2>=0.000
      47 -> 52         0.11025
      51 -> 52         0.69271
 This state for optimization and/or second-order correction.
 Total Energy, E(TD-HF/TD-KS) =  -518.238876339
```

2e

```
Excited State   1:      Singlet-A'     3.7298 eV  332.42 nm  f=0.2810
<S**2>=0.000
      47 -> 52         0.11394
      51 -> 52         0.69130
 This state for optimization and/or second-order correction.
 Total Energy, E(TD-HF/TD-KS) =  -518.235369600
```

2f

```
Excited State   1:      Singlet-A'     3.4047 eV  364.16 nm  f=0.3430
<S**2>=0.000
      47 -> 52         0.11266
      51 -> 52         0.69184
 This state for optimization and/or second-order correction.
 Total Energy, E(TD-HF/TD-KS) =  -518.215681542
```

3a

```
Excited State   1:      Singlet-A1     4.1934 eV  295.67 nm  f=0.3340
<S**2>=0.000
      53 -> 58        -0.12345
      57 -> 58         0.68681
 This state for optimization and/or second-order correction.
 Total Energy, E(TD-HF/TD-KS) =  -594.455829884
```

3b

```
Excited State   1:      Singlet-A1     3.4276 eV  361.73 nm  f=0.4477
<S**2>=0.000
      59 -> 64         0.11432
      63 -> 64         0.68950
 This state for optimization and/or second-order correction.
 Total Energy, E(TD-HF/TD-KS) =  -686.714355048
```

3c

```
Excited State   1:      Singlet-A      3.8245 eV  324.18 nm  f=0.5781
<S**2>=0.000
      73 -> 78         0.12924
      77 -> 78         0.67550
      77 -> 81         0.12729
 This state for optimization and/or second-order correction.
 Total Energy, E(TD-HF/TD-KS) =  -841.520801695
```

3d

```
Excited State   1:      Singlet-A1     3.6312 eV  341.44 nm  f=0.3432
<S**2>=0.000
      53 -> 58         0.12357
      57 -> 58         0.68930
 This state for optimization and/or second-order correction.
 Total Energy, E(TD-HF/TD-KS) =  -610.510160787
```

3e

```
Excited State   1:      Singlet-A'     3.9630 eV  312.86 nm  f=0.3174
<S**2>=0.000
      53 -> 58         0.12518
      57 -> 58         0.68771
 This state for optimization and/or second-order correction.
 Total Energy, E(TD-HF/TD-KS) =  -610.507031371
```

3f

```
Excited State   1:      Singlet-A'     3.6412 eV  340.50 nm  f=0.3734
<S**2>=0.000
      53 -> 58         0.12543
      57 -> 58         0.68835
 This state for optimization and/or second-order correction.
 Total Energy, E(TD-HF/TD-KS) =  -610.487434124
```

9

```
Excited State   1:      Singlet-B3U    2.5799 eV  480.57 nm  f=1.0083
<S**2>=0.000
      93 ->  94        0.69499
 This state for optimization and/or second-order correction.
 Total Energy, E(TD-HF/TD-KS) =  -956.899346500
```

7. Archive for DFT results

2a


```
1\1\GINC-LOCALHOST\FOpt\RB3LYP\def2TZVP\C5H14B10N1(1-)\PIOTR\08-Jun-20
 20\0\\#P B3LYP/Def2TZVP FOpt(tight) geom(noangle, nodistance) fcheck #
 P SCRF(Solvent=C6H5Cl)\\B10-1-Pyridine, C2v\\-1,1\B,0.,0.,0.1004669108
 \B,1.3009082385,0.,2.6639787152\B,0.,1.2995441316,2.65961425\B,-1.3009
 082385,0.,2.6639787152\B,0.,-1.2995441316,2.65961425\B,0.9276001595,-0
 .9194110773,1.1561683107\B,0.9276001595,0.9194110773,1.1561683107\B,-0
 .9276001595,0.9194110773,1.1561683107\B,-0.9276001595,-0.9194110773,1.
 1561683107\B,0.,0.,3.7508598325\N,0.,0.,-1.4257287664\C,1.1950061319,0
 .,-3.4894423797\C,-1.1950061319,0.,-3.4894423797\C,1.1634271736,0.,-2.
 109774387\C,-1.1634271736,0.,-2.109774387\C,0.,0.,-4.1981969318\H,2.42
 57431357,0.,3.0733868298\H,0.,2.4265774397,3.0625202638\H,-2.425743135
 7,0.,3.0733868298\H,0.,-2.4265774397,3.0625202638\H,2.1502769405,0.,-3
 .9944269036\H,-2.1502769405,0.,-3.9944269036\H,1.7112917794,-1.7099039
 616,0.7153541943\H,1.7112917794,1.7099039616,0.7153541943\H,-1.7112917
 794,1.7099039616,0.7153541943\H,-1.7112917794,-1.7099039616,0.71535419
 43\H,2.0578133254,0.,-1.5062694654\H,-2.0578133254,0.,-1.5062694654\H,
 0.,0.,-5.2797590301\H,0.,0.,4.942748398\\Version=ES64L-G09RevD.01\Stat
 e=1-A1\HF=-502.6726892\RMSD=4.723e-09\RMSF=2.108e-06\Dipole=0.,0.,-9.1
 622476\Quadrupole=8.2566338,-0.2269991,-8.0296346,0.,0.,0.\PG=C02V [C2
 (H1B1B1N1C1H1),SGV(C4H6B2),SGV'(H2B2),X(H4B4)]\\@
```


2b


```
1\1\GINC-LOCALHOST\FOpt\RB3LYP\def2TZVP\C6H13B10N2(1-)\PIOTR\03-May-20
 24\0\\#P B3LYP/Def2TZVP FOpt(tight) geom(noangle, nodistance) fcheck #
 P SCRF(Solvent=C6H5Cl) freq(noraman, readIso)\\H-10-B10-1-Pyridine-4-C
 N\\-1,1\B,0.,0.,-0.794296832\B,0.,1.3020268835,-3.3583965195\B,1.30056
 10047,0.,-3.3479006842\B,0.,-1.3020268835,-3.3583965195\B,-1.300561004
 7,0.,-3.3479006842\B,-0.9164750778,0.933554284,-1.8505688765\B,0.91647
 50778,0.933554284,-1.8505688765\B,0.9164750778,-0.933554284,-1.8505688
 765\B,-0.9164750778,-0.933554284,-1.8505688765\B,0.,0.,-4.4426087113\H
 ,0.,2.4248098901,-3.7705345699\H,2.4264214949,0.,-3.7509113441\H,0.,-2
 .4248098901,-3.7705345699\H,-2.4264214949,0.,-3.7509113441\H,0.,2.1549
 243325,3.2902862928\H,0.,-2.1549243325,3.2902862928\H,-1.7068543787,1.
 7133689117,-1.4054716068\H,1.7068543787,1.7133689117,-1.4054716068\N,0
 .,0.,0.7203436048\C,0.,1.2022160351,2.7822159154\C,0.,-1.2022160351,2.
 7822159154\C,0.,1.1651578427,1.4065233248\C,0.,-1.1651578427,1.4065233
 248\C,0.,0.,3.495150886\H,1.7068543787,-1.7133689117,-1.4054716068\H,-
 1.7068543787,-1.7133689117,-1.4054716068\H,0.,2.0611433934,0.806131656
 \H,0.,-2.0611433934,0.806131656\C,0.,0.,4.9228040749\N,0.,0.,6.0751480
 224\H,0.,0.,-5.6330256826\\Version=ES64L-G16RevC.01\State=1-A1\HF=-594
 .9468766\RMSD=3.825e-09\RMSF=3.866e-06\Dipole=0.,0.,7.6698867\Quadrupo
 le=18.870787,28.2056035,-47.0763905,0.,0.,0.\PG=C02V [C2(H1B1B1N1C1C1N
 1),SGV(C4H6B2),SGV'(H2B2),X(H4B4)]\\@
```


2c


```
1\1\GINC-LOCALHOST\FOpt\RB3LYP\def2TZVP\C10H17B10N2(1-)\PIOTR\03-May-2
 024\0\\#P B3LYP/Def2TZVP FOpt(tight) geom(noangle, nodistance) fcheck
 #P freq(noraman, readIso) SCRF(Solvent=C6H5Cl)\\(H-10-B10)-Bipyridine\
 \-1,1\N,0.0000000032,0.,-6.4469962118\C,-1.1325830489,0.3702888097,-4.
 363152164\C,-1.0809750779,0.3567362492,-5.7498980183\C,1.1325830532,-0
 .3702888097,-4.3631521629\C,1.0809750835,-0.3567362492,-5.7498980172\C
 ,0.0000000018,0.,-3.6338045393\H,-1.9517141415,0.6508259563,-6.3258108
 269\H,-2.0385026595,0.6897600377,-3.8663331805\H,2.0385026633,-0.68976
 00377,-3.8663331785\H,1.9517141477,-0.6508259563,-6.325810825\C,1.1178
```

941234,0.3094304151,-0.0475032779\C,-1.1178941233,-0.3094304151,-0.047
503279\C,1.1485654146,0.3151747751,-1.4239345329\C,-1.1485654132,-0.31
51747751,-1.423934534\C,0.0000000011,0.,-2.1568976438\N,-0.0000000003,
0.,0.6425860127\H,1.9819845273,0.5561193385,0.549616759\H,-1.981984527
8,-0.5561193385,0.5496167571\H,2.0694268967,0.5897299499,-1.9175455321
\H,-2.0694268948,-0.5897299499,-1.9175455341\B,-0.0000000011,0.,2.1653
421041\B,0.6642897452,1.1248828259,3.2215772765\B,-1.1323072452,0.6519
001437,3.2217200577\B,-0.6642897484,-1.1248828259,3.2215772758\B,1.132
3072421,-0.6519001437,3.2217200588\B,1.2581940833,0.3312037156,4.72942
23375\B,-0.3307341236,1.256966047,4.723985337\B,-1.2581940879,-0.33120
37156,4.7294223363\B,0.330734119,-1.256966047,4.7239853374\B,-0.000000
0029,0.,5.8156502085\H,1.2206063356,2.0892573551,2.7815609509\H,-2.090
9550509,1.2175066653,2.7812740035\H,-1.2206063383,-2.0892573551,2.7815
609497\H,2.0909550482,-1.2175066653,2.7812740055\H,2.3456228355,0.6170
184014,5.1397169033\H,-0.617065178,2.3467393359,5.1271501576\H,-2.3456
228405,-0.6170184014,5.139716901\H,0.6170651729,-2.3467393359,5.127150
1582\H,-0.0000000034,0.,7.0068174084\\Version=ES64L-G16RevC.01\State=1
-A\HF=-749.8559462\RMSD=4.049e-09\RMSF=1.611e-06\Dipole=0.,0.,-11.8910
986\Quadrupole=45.3409219,32.8370539,-78.1779758,0.0867858,0.,0.\PG=C0
2 [C2(H1B1B1N1C1C1N1),X(C8H16B8)]\\@

2d

1\1\GINC-LOCALHOST\FOpt\RB3LYP\def2TZVP\C4H13B10N2(1-)\PIOTR\21-Oct-20
21\0\\#P B3LYP/Def2TZVP FOpt(tight) geom(noangle, nodistance) fcheck #
P SCRF(Solvent=C6H5Cl)\\H-10-B10-1-Pyrazine, C2v\\-1,1\B,0.,0.,-0.1229
479957\B,0.,1.3019293158,-2.6858375084\B,1.3004006039,0.,-2.6748617754
\B,0.,-1.3019293158,-2.6858375084\B,-1.3004006039,0.,-2.6748617754\B,-
0.9168676298,0.9335602696,-1.17774724\B,0.9168676298,0.9335602696,-1.1
7774724\B,0.9168676298,-0.9335602696,-1.17774724\B,-0.9168676298,-0.93
35602696,-1.17774724\B,0.,0.,-3.7696389157\H,0.,2.4250018359,-3.097327
5338\H,2.4264821087,0.,-3.0775828096\H,0.,-2.4250018359,-3.0973275338\
H,-2.4264821087,0.,-3.0775828096\H,0.,2.0658074434,4.0203234993\H,0.,-
2.0658074434,4.0203234993\H,-1.7074560236,1.712762803,-0.732125898\H,1
.7074560236,1.712762803,-0.732125898\N,0.,0.,1.3910273508\C,0.,1.13229
8287,3.4718312932\C,0.,-1.132298287,3.4718312932\C,0.,1.1537767368,2.0
89792302\C,0.,-1.1537767368,2.089792302\N,0.,0.,4.176572637\H,1.707456
0236,-1.712762803,-0.732125898\H,-1.7074560236,-1.712762803,-0.7321258
98\H,0.,2.0669002653,1.5157200244\H,0.,-2.0669002653,1.5157200244\H,0.
,0.,-4.9605996468\\Version=ES64L-G09RevD.01\State=1-A1\HF=-518.7077078
\RMSD=8.186e-09\RMSF=2.690e-06\Dipole=0.,0.,7.3503858\Quadrupole=5.602
8695,14.4160869,-20.0189563,0.,0.,0.\PG=C02V [C2(H1B1B1N1N1),SGV(C4H6B
2),SGV'(H2B2),X(H4B4)]\\@

2e

1\1\GINC-LOCALHOST\FOpt\RB3LYP\def2TZVP\C4H13B10N2(1-)\PIOTR\21-Oct-20
21\0\\#P B3LYP/Def2TZVP FOpt(tight) geom(noangle, nodistance) fcheck #
P SCRF(Solvent=C6H5Cl)\\H-10-B10-1-Pyrimidine, Cs\\-1,1\B,-2.687356185
9,1.3046235609,0.\B,-2.6809971622,-1.2981256422,0.\B,-2.6778295403,0.0
026936693,-1.3001485358\B,-2.6778295403,0.0026936693,1.3001485358\B,-1
.1792910324,0.9348109775,0.9192673533\B,-1.1792910324,0.9348109775,-0.
9192673533\B,-1.17384413,-0.9244072562,-0.9193845532\B,-1.17384413,-0.
9244072562,0.9193845532\B,-0.1235623649,0.007822467,0.\B,-3.7692327323
,0.0000050802,0.\H,-3.1004784589,2.4278134309,0.\H,-3.0894644957,-2.42
28031586,0.\H,-3.081171277,0.0021438336,-2.4266193723\H,-3.081171277,0
.0021438336,2.4266193723\H,-0.7366583381,1.7194771394,1.7075270481\H,-
0.7366583381,1.7194771394,-1.7075270481\H,-0.7324326642,-1.7060681712,

-1.7106231108\H,-0.7324326642,-1.7060681712,1.7106231108\N,1.399208340
2,0.0173792948,0.\C,3.4734892678,1.1582848866,0.\C,2.0956605075,1.1723
055056,0.\C,4.1035555695,-0.0775332018,0.\C,2.0995586387,-1.1401372809
,0.\N,3.4120031902,-1.221946358,0.\H,4.0318866844,2.082475077,0.\H,1.5
095658755,2.0787871738,0.\H,5.18360512,-0.1595585468,0.\H,1.5089429495
,-2.0450843964,0.\H,-4.9610227296,-0.0049528923,0.\\Version=ES64L-G09R
evD.01\State=1-A'\HF=-518.7138242\RMSD=3.120e-09\RMSF=6.059e-06\Dipole
=8.3427299,0.9641201,0.\Quadrupole=-10.4414713,7.365973,3.0754983,7.58
94702,0.,0.\PG=CS [SG(C4H7B4N2),X(H6B6)]\\@

2f

1\1\GINC-LOCALHOST\FOpt\RB3LYP\def2TZVP\C4H13B10N2(1-)\PIOTR\21-Oct-20
21\0\\#P B3LYP/Def2TZVP FOpt(tight) geom(noangle, nodistance) fcheck #
P SCRF(Solvent=C6H5Cl)\\H-10-B10-1-Pyridazine, C2v\\-1,1\B,-1.31399029
85,2.6564930231,0.\B,1.2890235799,2.7059962756,0.\B,-0.0115736315,2.67
00316959,1.3000664268\B,-0.0115736315,2.6700316959,-1.3000664268\B,-0.
9121222652,1.1558642852,-0.9154542743\B,-0.9121222652,1.1558642852,0.9
154542743\B,0.9538827486,1.1898331435,0.9177881266\B,0.9538827486,1.18
98331435,-0.9177881266\B,0.0450126292,0.1159881291,0.\B,-0.0330770013,
3.7644455823,0.\H,-2.4457126641,3.0458970245,0.\H,2.4031882014,3.14203
85151,0.\H,-0.0203509629,3.0733738681,2.4265534869\H,-0.0203509629,3.0
733738681,-2.4265534869\H,-1.6854485621,0.6929599546,-1.703279714\H,-1
.6854485621,0.6929599546,1.703279714\H,1.7394755025,0.7658031001,1.712
3557568\H,1.7394755025,0.7658031001,-1.7123557568\N,0.0244018562,-1.39
8653656,0.\C,-1.182571361,-3.4473919281,0.\C,1.1806196113,-3.362384064
5,0.\C,-1.1423933225,-2.0644015338,0.\C,0.0165836562,-4.1297716781,0.\
N,1.193847856,-2.0409466038,0.\H,-2.1366031202,-3.9544697263,0.\H,2.16
06382308,-3.8217362578,0.\H,-2.0267525102,-1.4471789287,0.\H,0.0662397
513,-5.20910808,0.\H,-0.0536150789,4.9560108256,0.\\Version=ES64L-G09R
evD.01\State=1-A'\HF=-518.6823685\RMSD=5.861e-09\RMSF=7.208e-06\Dipole
=-0.905646,-9.3214533,0.\Quadrupole=4.0706372,-3.1459863,-0.9246509,2.
5308803,0.,0.\PG=CS [SG(C4H7B4N2),X(H6B6)]\\@

3a

1\1\GINC-LOCALHOST\FOpt\RB3LYP\def2TZVP\C6H13B10N2(1-)\PIOTR\01-Dec-20
20\0\\#P B3LYP/Def2TZVP FOpt(tight) geom(noangle, nodistance) fcheck #
P SCRF(Solvent=C6H5Cl) guess=check freq(noraman, readIso)\\CB-10-B10-1
-Pyridine, C2v\\-1,1\B,0.,1.3079071673,-2.0619270955\B,0.,-1.307907167
3,-2.0619270955\B,1.3067100182,0.,-2.0587438247\B,-1.3067100182,0.,-2.
0587438247\B,-0.9217129395,0.9279467331,-0.5610664469\B,0.9217129395,0
.9279467331,-0.5610664469\B,0.9217129395,-0.9279467331,-0.5610664469\B
,-0.9217129395,-0.9279467331,-0.5610664469\B,0.,0.,0.4923771873\B,0.,0
.,-3.133518571\N,0.,0.,2.0193586377\C,0.,1.195460812,4.0812752987\C,0.
,-1.195460812,4.0812752987\C,0.,1.1639605706,2.7017024211\C,0.,-1.1639
605706,2.7017024211\C,0.,0.,4.7889551812\H,0.,2.4235527178,-2.48552323
07\H,0.,-2.4235527178,-2.4855232307\H,2.424373285,0.,-2.4766032996\H,-
2.424373285,0.,-2.4766032996\H,-1.7102734865,1.7103646951,-0.120083531
8\H,1.7102734865,1.7103646951,-0.1200835318\H,1.7102734865,-1.71036469
51,-0.1200835318\H,-1.7102734865,-1.7103646951,-0.1200835318\H,0.,2.15
03901644,4.5866550378\H,0.,-2.1503901644,4.5866550378\H,0.,2.058744609
3,2.0987874981\H,0.,-2.0587446093,2.0987874981\H,0.,0.,5.870510495\C,0
.,0.,-4.6656155395\N,0.,0.,-5.8232331819\\Version=ES64L-G09RevD.01\Sta
te=1-A1\HF=-594.9937748\RMSD=2.640e-09\RMSF=1.736e-06\Dipole=0.,0.,10.
6869722\Quadrupole=6.0651128,14.7222939,-20.7874067,0.,0.,0.\PG=C02V [
C2(H1C1N1B1B1C1N1),SGV(C4H6B2),SGV'(H2B2),X(H4B4)]\\@

3b

1\1\GINC-LOCALHOST\FOpt\RB3LYP\def2TZVP\C7H12B10N3(1-)\PIOTR\22-Oct-20
21\0\\#P B3LYP/Def2TZVP FOpt(tight) geom(noangle, nodistance) fcheck #
P SCRF(Solvent=C6H5Cl) freq(noraman, readIso) guess=check\\CN-10-B10-1
-Pyridine-4-CN\\-1,1\B,0.,0.,-0.1613091649\B,0.,1.3087450364,-2.715078
4525\B,1.3075171665,0.,-2.7069488745\B,0.,-1.3087450364,-2.7150784525\
B,-1.3075171665,0.,-2.7069488745\B,-0.9198999511,0.9324472698,-1.21394
05683\B,0.9198999511,0.9324472698,-1.2139405683\B,0.9198999511,-0.9324
472698,-1.2139405683\B,-0.9198999511,-0.9324472698,-1.2139405683\B,0.,
0.,-3.7840869309\C,0.,0.,-5.3160920344\N,0.,0.,-6.4733642318\H,0.,2.42
31338283,-3.1398915778\H,2.4246592156,0.,-3.124039995\H,0.,-2.42313382
83,-3.1398915778\H,-2.4246592156,0.,-3.124039995\H,0.,2.1548377586,3.9
254121596\H,0.,-2.1548377586,3.9254121596\H,-1.7084083828,1.7114089327
,-0.7687796371\H,1.7084083828,1.7114089327,-0.7687796371\N,0.,0.,1.357
2431018\C,0.,1.2024710456,3.4168941949\C,0.,-1.2024710456,3.4168941949
\C,0.,1.1651547374,2.0405704699\C,0.,-1.1651547374,2.0405704699\C,0.,0
.,4.1278192478\H,1.7084083828,-1.7114089327,-0.7687796371\H,-1.7084083
828,-1.7114089327,-0.7687796371\H,0.,2.0615651053,1.4406796373\H,0.,-2
.0615651053,1.4406796373\C,0.,0.,5.5565157348\N,0.,0.,6.708426449\\Ver
sion=ES64L-G09RevD.01\State=1-A1\HF=-687.2664672\RMSD=6.915e-10\RMSF=1
.892e-06\Dipole=0.,0.,9.3509813\Quadrupole=27.9900502,37.5752245,-65.5
652747,0.,0.,0.\PG=C02V [C2(N1C1B1B1N1C1C1N1),SGV(C4H6B2),SGV'(H2B2),X
(H4B4)]\\@

3c

1\1\GINC-LOCALHOST\FOpt\RB3LYP\def2TZVP\C11H16B10N3(1-)\PIOTR\22-Oct-2
021\0\\#P B3LYP/Def2TZVP FOpt(tight) geom(noangle, nodistance) fcheck
#P freq(noraman, readIso) SCRF(Solvent=C6H5Cl)\\(CN-10-B10)-Bipyridine
\\-1,1\N,-0.0000000049,0.,7.0739651532\C,-1.1386166059,-0.3521771096,4
.9905907913\C,-1.0866112416,-0.3397099541,6.3774156479\C,1.1386165989,
0.3521771096,4.9905907929\C,1.0866112327,0.3397099541,6.3774156494\C,-
0.000000003,0.,4.2621075336\H,-1.961801452,-0.619732928,6.9535072062\H
,-2.0496082624,-0.6567918192,4.4937702701\H,2.0496082562,0.6567918192,
4.493770273\H,1.9618014423,0.619732928,6.9535072089\C,1.1133815752,-0.
3267068997,0.6763821018\C,-1.1133815762,0.3267068997,0.6763821002\C,1.
1437874277,-0.3335911793,2.0527944925\C,-1.1437874306,0.3335911793,2.0
527944909\C,-0.0000000019,0.,2.7848421018\N,0.,0.,-0.011823195\H,1.973
9401085,-0.586371597,0.0795914931\H,-1.9739401087,0.586371597,0.079591
4904\H,2.0599578161,-0.6226367926,2.5466251805\H,-2.0599578197,0.62263
67926,2.5466251776\B,0.0000000011,0.,-1.536312468\B,0.688177619,-1.112
4087167,-2.5897140119\B,-1.1183136661,-0.6791740557,-2.58999237\B,-0.6
881776154,1.1124087167,-2.5897140129\B,1.1183136697,0.6791740557,-2.58
99923684\B,1.2714609295,-0.3051132831,-4.0911630498\B,-0.3044459729,-1
.2704317314,-4.0872158504\B,-1.2714609238,0.3051132831,-4.0911630516\B
,0.3044459786,1.2704317314,-4.08721585\B,0.0000000036,0.,-5.1626744129
\C,0.0000000047,0.,-6.6943886819\N,0.0000000055,0.,-7.8519245941\H,1.2
652130327,-2.0625029034,-2.150633747\H,-2.0632585371,-1.2634619779,-2.
1496664779\H,-1.2652130297,2.0625029034,-2.1506337488\H,2.0632585401,1
.2634619779,-2.149666475\H,2.3562752874,-0.5654466506,-4.5148538505\H,
-0.5651958903,-2.3573663391,-4.5047708452\H,-2.3562752811,0.5654466506
,-4.5148538538\H,0.5651958966,2.3573663391,-4.5047708444\\Version=ES64
L-G09RevD.01\State=1-A\HF=-842.1765546\RMSD=8.470e-10\RMSF=5.933e-06\D
ipole=0.,0.,13.5223199\Quadrupole=56.3709895,43.6774753,-100.0484648,-
0.3190929,0.0000001,0.\PG=C02 [C2(N1C1C1N1B1B1C1N1),X(C8H16B8)]\\@

3c[H]

1\1\GINC-LOCALHOST\FOpt\RB3LYP\def2TZVP\C11H17B10N3\PIOTR\29-Apr-2024\
0\\#P B3LYP/Def2TZVP FOpt(tight) geom(noangle, nodistance) fcheck #P f
req(noraman, readIso) SCRF(Solvent=C6H5Cl)\\(CN-10-B10)-Bipyridine pro
tonated\\0,1\N,0.0000000034,0.,-6.9424421371\C,-1.1418907412,0.3749117
594,-4.9224872344\C,-1.1193586052,0.3696994689,-6.295275817\C,1.141890
746,-0.3749117594,-4.9224872333\C,1.1193586114,-0.3696994689,-6.295275
8159\C,0.0000000021,0.,-4.1974704754\H,-1.9619394153,0.6566932205,-6.9
051149755\H,-2.0437614165,0.6962289461,-4.4243875803\H,2.0437614209,-0
.6962289461,-4.4243875783\H,1.9619394221,-0.6566932205,-6.9051149736\C
,1.1321726183,0.2655057447,-0.6225993008\C,-1.1321726177,-0.2655057447
,-0.6225993019\C,1.1646400276,0.2655231271,-1.9980232439\C,-1.16464002
57,-0.2655231271,-1.998023245\C,0.0000000013,0.,-2.7262003265\N,0.,0.,
0.065088937\H,2.004847691,0.4781636998,-0.0253084245\H,-2.004847691,-0
.4781636998,-0.0253084264\H,2.0976791691,0.5038907007,-2.4869778178\H,
-2.0976791667,-0.5038907007,-2.4869778198\B,-0.0000000008,0.,1.5788261
512\B,0.6518801011,1.1369495543,2.6324146032\B,-1.1482462931,0.6313000
006,2.6325538574\B,-0.6518801037,-1.1369495543,2.6324146026\B,1.148246
2905,-0.6313000006,2.6325538585\B,1.2603275302,0.3536550915,4.13373522
08\B,-0.3541074747,1.259030794,4.1232116656\B,-1.2603275343,-0.3536550
915,4.1337352196\B,0.3541074707,-1.259030794,4.123211666\B,-0.00000000
25,0.,5.2022016741\C,-0.0000000033,0.,6.7337376358\N,-0.0000000039,0.,
7.8907453119\H,1.1875730686,2.1062699619,2.1856441315\H,-2.1106949701,
1.1805649168,2.1871673063\H,-1.1875730707,-2.1062699619,2.1856441303\H
,2.1106949679,-1.1805649168,2.1871673084\H,2.3325461023,0.6549936179,4
.5590890168\H,-0.6562279849,2.3342597054,4.5398045239\H,-2.3325461068,
-0.6549936179,4.5590890145\H,0.6562279804,-2.3342597054,4.5398045245\H
,0.0000000039,0.,-7.9558064995\\Version=ES64L-G16RevC.01\State=1-A\HF=
-842.6137693\RMSD=2.897e-09\RMSF=2.027e-06\Dipole=0.,0.,-24.4482797\Qu
adrupole=4.8576375,-11.4967174,6.6390799,-1.1687727,0.,0.\PG=C02 [C2(H
1N1C1C1N1B1B1C1N1),X(C8H16B8)]\\@

3d

1\1\GINC-LOCALHOST\FOpt\RB3LYP\def2TZVP\C5H12B10N3(1-)\PIOTR\22-Oct-20
21\0\\#P B3LYP/Def2TZVP FOpt(tight, ReadFC) geom(noangle, nodistance)
fcheck #P freq(noraman, readIso) SCRF(Solvent=C6H5Cl)\\CB-10-B10-1-Pyr
idazine, C2v\\-1,1\B,0.,0.,0.1215162797\B,1.3087093121,0.,2.674141232\
B,0.,1.3075516717,2.6654633649\B,-1.3087093121,0.,2.674141232\B,0.,-1.
3075516717,2.6654633649\B,0.9328396595,-0.919964521,1.1726887165\B,0.9
328396595,0.919964521,1.1726887165\B,-0.9328396595,0.919964521,1.17268
87165\B,-0.9328396595,-0.919964521,1.1726887165\B,0.,0.,3.7426944463\C
,0.,0.,5.2748067964\N,0.,0.,6.4320715756\H,2.423042366,0.,3.0991299906
\H,0.,2.4246170559,3.0828255984\H,-2.423042366,0.,3.0991299906\H,0.,-2
.4246170559,3.0828255984\H,2.065809921,0.,-4.0239160494\H,-2.065809921
,0.,-4.0239160494\H,1.71151067,-1.7085889597,0.7275810848\H,1.71151067
,1.7085889597,0.7275810848\N,0.,0.,-1.3964737416\C,1.1329379046,0.,-3.
4746493922\C,-1.1329379046,0.,-3.4746493922\C,1.1540851967,0.,-2.09186
48473\C,-1.1540851967,0.,-2.0918648473\N,0.,0.,-4.1768308468\H,-1.7115
1067,1.7085889597,0.7275810848\H,-1.71151067,-1.7085889597,0.727581084
8\H,2.0671780567,0.,-1.5176879298\H,-2.0671780567,0.,-1.5176879298\\Ve
rsion=ES64L-G09RevD.01\State=1-A1\HF=-611.0275565\RMSD=3.699e-09\RMSF=
1.731e-08\Dipole=0.,0.,-8.9456396\Quadrupole=22.0768924,13.0038919,-35
.0807842,0.,0.,0.\PG=C02V [C2(N1C1B1B1N1N1),SGV(C4H6B2),SGV'(H2B2),X(H
4B4)]\\@

3e

1\1\GINC-LOCALHOST\FOpt\RB3LYP\def2TZVP\C5H12B10N3(1-)\PIOTR\23-Oct-20

21\0\\#P B3LYP/Def2TZVP FOpt(tight) geom(noangle, nodistance) fcheck #
P freq(noraman, readIso) SCRF(Solvent=C6H5Cl)\\CB-10-B10-1-Pyrimidine,
Cs\\-1,1\B,-2.0573166384,1.2980990388,0.\B,-2.0446217982,-1.31833325,
0.\B,-2.0459258902,-0.0091164396,-1.3071199045\B,-2.0459258902,-0.0091
164396,1.3071199045\B,-0.5543017275,0.9268257963,0.9213616596\B,-0.554
3017275,0.9268257963,-0.9213616596\B,-0.5456435871,-0.9324637434,-0.92
22177582\B,-0.5456435871,-0.9324637434,0.9222177582\B,0.5001823619,0.0
015689683,0.\B,-3.1211360924,-0.0149784751,0.\C,-4.6529834617,-0.02200
83335,0.\N,-5.8104132624,-0.0255029153,0.\H,-2.4861720863,2.4113803002
,0.\H,-2.4637975295,-2.4353089869,0.\H,-2.4637283136,-0.0115821826,-2.
4245027011\H,-2.4637283136,-0.0115821826,2.4245027011\H,-0.115023442,1
.7111217002,1.7087414521\H,-0.115023442,1.7111217002,-1.7087414521\H,-
0.1005107042,-1.7121858165,-1.7104019431\H,-0.1005107042,-1.7121858165
,1.7104019431\N,2.0245483313,0.0215625336,0.\C,4.0864601693,1.18227158
4,0.\C,2.7088233246,1.1832365411,0.\C,4.7267586087,-0.0483285344,0.\C,
2.733233023,-1.1298157697,0.\N,4.0464624133,-1.1990816159,0.\H,4.63652
79449,2.1113515764,0.\H,2.1149578006,2.0845966444,0.\H,5.8075289788,-0
.1204651097,0.\H,2.1514258196,-2.0406085718,0.\\Version=ES64L-G09RevD.
01\State=1-A'\HF=-611.0342201\RMSD=2.152e-09\RMSF=3.738e-06\Dipole=9.8
67653,1.0226411,0.\Quadrupole=-24.3530238,14.4705659,9.8824579,8.59382
61,0.,0.\PG=CS [SG(C5H6B4N3),X(H6B6)]\\@

3f

1\1\GINC-LOCALHOST\FOpt\RB3LYP\def2TZVP\C5H12B10N3(1-)\PIOTR\23-Oct-20
21\0\\#P B3LYP/Def2TZVP FOpt(tight) geom(noangle, nodistance) fcheck #
P freq(noraman, readIso) SCRF(Solvent=C6H5Cl)\\CB-10-B10-1-Pyridazine,
C2v\\-1,1\B,-2.0243672929,1.2980239866,0.\B,-2.052087762,-1.318707757
6,0.\B,-2.0290011861,-0.0097562348,-1.3069652219\B,-2.0290011861,-0.00
97562348,1.3069652219\B,-0.5263969939,0.9032497081,0.9180442233\B,-0.5
263969939,0.9032497081,-0.9180442233\B,-0.5455213364,-0.9618367443,-0.
9210990327\B,-0.5455213364,-0.9618367443,0.9210990327\B,0.5165874088,-
0.0449388433,0.\B,-3.1067968158,0.0017817627,0.\C,-4.638402925,0.01938
2752,0.\N,-5.7957834143,0.0350883964,0.\H,-2.4364133719,2.4177948046,0
.\H,-2.4912991628,-2.4279603511,0.\H,-2.4466988488,-0.0051538028,-2.42
45259447\H,-2.4466988488,-0.0051538028,2.4245259447\H,-0.0709631288,1.
6791583396,1.7046245879\H,-0.0709631288,1.6791583396,-1.7046245879\H,-
0.1164241699,-1.7444111426,-1.7131749004\H,-0.1164241699,-1.7444111426
,1.7131749004\N,2.0338119084,-0.0092990927,0.\C,4.0711196347,1.2125276
148,0.\C,4.001634236,-1.1526500378,0.\C,2.6873915574,1.1618951612,0.\C
,4.7598316031,0.0184092116,0.\N,2.6806440055,-1.1742805899,0.\H,4.5715
231844,2.1699737772,0.\H,4.4685186896,-2.1288937025,0.\H,2.0637906719,
2.0419828835,0.\H,5.8395208464,-0.0238122812,0.\\Version=ES64L-G09RevD
.01\State=1-A'\HF=-611.0028215\RMSD=2.370e-09\RMSF=3.586e-06\Dipole=10
.9266345,0.9732978,0.\Quadrupole=-15.1606946,10.218703,4.9419915,3.935
1228,0.,0.\PG=CS [SG(C5H6B4N3),X(H6B6)]\\@

4c

1\1\GINC-LOCALHOST\FOpt\RB3LYP\def2SVP\C16H27B10Fe1N3P2\PIOTR\16-Nov-2
023\0\\#P B3LYP/Def2SVP FOpt(ModRedundant) SCF(Direct) SCRF(COSMO, Sol
vent=CH2CL2) #P Geom=(NoDistance,NoAngle, Step=9) fcheck freq(noRaman)
\\Bipyr-B10CN-Fe(PH3)2Cp with frozen bipoyridyl two angles\\0,1\B,1.34
15639959,0.62478178,-1.1492048561\B,1.339485244,-0.6169696013,1.164240
8059\B,1.3418028317,-1.1555364662,-0.6067240916\B,1.3395207571,1.16469
72964,0.6224925329\B,-0.1629044804,1.2609744405,-0.3768209978\B,-0.160
1482441,-0.3791353009,-1.2496803346\B,-0.1627300655,-1.2538164022,0.38
89879009\B,-0.1627767417,0.3862079595,1.26227323\B,-1.2426313125,0.003

2810408,0.0053125517\B,2.4069355134,0.0054846357,0.0094304334\H,1.7866
232216,1.1522028903,-2.1433646798\H,1.7831520306,-1.1442142009,2.15904
84196\H,1.7871063302,-2.1468403824,-1.1390825951\H,1.7817262196,2.1567
272217,1.1559931407\H,-0.5954169569,2.3412960506,-0.7064235279\H,-0.58
65621124,-0.70893718,-2.3323612277\H,-0.5948517833,-2.3345575631,0.717
5683043\H,-0.5916380572,0.715625523,2.3439354057\C,-2.7754748433,0.002
4981021,0.0067294686\N,-3.9398713721,0.0013013527,0.0133445583\C,-5.83
12651614,0.171778669,2.1727966323\H,-4.9065789274,0.208846922,2.748130
6962\C,-6.4940170559,1.2941378618,1.6061840162\C,-6.5174766876,-1.0127
361553,1.7940679925\H,-6.206446247,2.3381412057,1.7214206141\C,-7.6308
425338,0.7962937866,0.8789157968\H,-6.2509518516,-2.029933228,2.077366
5714\C,-7.6449212533,-0.6165555729,0.9924175884\H,-8.3618431393,1.3987
918882,0.3397121946\H,-8.389523963,-1.28305112,0.5575593139\Fe,-5.8586
507349,0.0001345678,0.0450445554\P,-5.9111882544,-1.7611906362,-1.3445
035104\H,-6.0843112573,-3.0474677617,-0.7712565422\H,-6.9077680846,-1.
8560696237,-2.3522920744\H,-4.7739327216,-2.0332673436,-2.1481349791\P
,-5.9068493585,1.4905598821,-1.6290772092\H,-6.9140108476,1.4244015629
,-2.6287345313\H,-6.0515433299,2.8608574787,-1.2905787019\H,-4.7751934
831,1.5949104387,-2.4785887102\C,4.6260479268,1.1173990708,-0.32545965
01\C,4.6205416726,-1.1122638996,0.3597918349\C,6.0106640595,1.14853016
62,-0.3238571079\H,4.0303777023,1.9866112022,-0.6030245899\C,6.0049856
251,-1.144080623,0.3780151282\H,4.0205520284,-1.9809230801,0.629560259
1\H,6.5087548268,2.0709506883,-0.6234320077\H,6.4982039458,-2.06669684
04,0.6849562519\N,3.9361487682,0.0026600588,0.0119272619\C,6.744311028
4,0.0023402449,0.0334300823\C,8.227638128,0.0020662271,0.0440311945\C,
8.9583585943,1.1617613393,0.3544803482\C,8.9620127468,-1.1575676715,-0
.2579826141\C,10.3539988079,1.1074058124,0.348481031\H,8.4571326406,2.
0944077277,0.6198108351\C,10.3575022588,-1.1015034643,-0.2420150834\H,
8.4639876408,-2.0907950611,-0.5272463286\H,10.9318213604,2.0051380866,
0.5965118168\H,10.9381749183,-1.9986390383,-0.4855037961\N,11.05070403
78,0.0036260358,0.0547749795\\Version=ES64L-G09RevD.01\State=1-A\HF=-2
984.3144537\RMSD=6.783e-09\RMSF=6.370e-05\Dipole=-3.2591403,-0.138573,
-1.4364792\Quadrupole=71.8531979,-27.4108835,-44.4423144,1.6677061,18.
2325521,-1.1320539\PG=C01 [X(C16H27B10Fe1N3P2)]\\@

4d

1\1\GINC-LOCALHOST\FOpt\RB3LYP\def2SVP\C10H23B10Fe1N3P2\PIOTR\14-Oct-2
023\0\\#P B3LYP/Def2SVP FOpt SCF(Direct, tight) SCRF(COSMO, Solvent=CH
2CL2) #P Geom=(NoDistance,NoAngle) fcheck freq(noRaman)\\Pyrazine-B10C
N-Fe(PH3)2Cp\\0,1\B,3.195061705,-0.7283421998,1.0899704326\B,3.2040677
872,0.6590063962,-1.1426217893\B,3.196833744,1.0884015104,0.6576382425
\B,3.2014714928,-1.1578375512,-0.7102353117\B,1.6931806883,-1.31306976
56,0.2727918467\B,1.6953743289,0.2686416207,1.2458330693\B,1.696294365
5,1.2440582794,-0.3369554954\B,1.7049058029,-0.337437419,-1.3103203866
\B,0.616916167,-0.03436754,-0.0365023674\B,4.2613250228,-0.0350014933,
-0.0222035943\H,3.6363170798,-1.3141905432,2.0517422589\H,3.6528277796
,1.2448168713,-2.1009099434\H,3.6421800556,2.0431390461,1.2518551785\H
,3.6509562294,-2.1126953601,-1.3009879226\H,1.2601969626,-2.4113894939
,0.5330758572\H,1.2635929653,0.5311051167,2.3442001369\H,1.2656441394,
2.3427450142,-0.6002481766\H,1.281902363,-0.5991956329,-2.4121033833\C
,-0.9161975409,-0.0309328256,-0.0377285365\N,-2.080412652,-0.027484028
9,-0.0348571566\C,-4.019358921,-1.8301810899,-1.1700478412\H,-3.109581
1573,-2.3127828333,-1.5261183196\C,-4.6300024345,-2.0535521439,0.09463
21212\C,-4.7343826582,-0.814582109,-1.8574738349\H,-4.3117062563,-2.77
47344716,0.8458570639\C,-5.7633561266,-1.1732661253,0.1817614031\H,-4.
5080955484,-0.427373776,-2.849866971\C,-5.82773622,-0.4119892865,-1.01
22621631\H,-6.4594999176,-1.1050715594,1.0177749975\H,-6.5821852272,0.

3373881467,-1.250549081\Fe,-3.9984104697,-0.0290910421,-0.0237308981\P ,-4.0352984724,2.1330430274,-0.6247270843\H,-4.2752891077,2.4324958671 ,-1.9905256502\H,-4.979618617,3.0292591244,-0.0559841079\H,-2.86573844 47,2.9134140173,-0.4333074533\P,-3.9976295505,0.4428501411,2.169453905 8\H,-4.9949276977,1.2873580614,2.7259524602\H,-4.1196234637,-0.6384838 988,3.0800876761\H,-2.8542283566,1.0699716748,2.728223824\C,6.47932324 38,-1.1272168066,0.3778989142\C,6.4829479701,1.0574309935,-0.401027783 7\C,7.8718282995,-1.1062713314,0.3787767728\H,5.9030078062,-1.99909747 2,0.6855953856\C,7.8753747784,1.0373585809,-0.3865355437\H,5.909526903 2,1.9290073338,-0.7149120352\H,8.4218488952,-1.9958464526,0.6996578928 \H,8.4283681286,1.9272620668,-0.7013469583\N,5.7881945989,-0.035043968 6,-0.0152091805\N,8.572485537,-0.0342136699,0.00005553\\Version=ES64L-G09RevD.01\State=1-A\HF=-2753.4082294\RMSD=3.742e-09\RMSF=6.661e-06\Dipole=-3.9982269,1.293214,0.8147416\Quadrupole=53.0315733,-23.7799037,-29.2516696,-11.2767639,-6.6138551,-4.5793285\PG=C01 [X(C10H23B10Fe1N3P2)]\\@

9

1\1\GINC-LOCALHOST\FOpt\RB3LYP\def2TZVP\C6H20B20N4(2-)\PIOTR\23-Oct-2021\0\\#P B3LYP/Def2TZVP FOpt(tight) geom(noangle, nodistance) fcheck #P freq(noraman, readIso) SCRF(Solvent=C6H5Cl)\\1,4-bis-(CN-10-B10)-Pyrazine, cs\\-2,1\N,9.2061161267,0.,0.\C,8.0490176435,0.,0.\B,2.89465583 22,0.,0.\B,3.9491840945,-0.9378232072,-0.9172996095\B,3.9491840945,-0. 9378232072,0.9172996095\B,3.9491840945,0.9378232072,0.9172996095\B,3.9 491840945,0.9378232072,-0.9172996095\B,5.4367596833,0.,-1.3083601717\B ,5.4508410151,-1.3096594903,0.\B,5.4367596833,0.,1.3083601717\B,5.4508 410151,1.3096594903,0.\B,6.5171980664,0.,0.\H,3.500153899,-1.712672329 9,-1.7060911123\H,3.500153899,-1.7126723299,1.7060911123\H,3.500153899 ,1.7126723299,1.7060911123\H,5.85311981,0.,-2.4251284885\H,5.877406170 9,-2.4227679947,0.\H,5.85311981,0.,2.4251284885\H,5.8774061709,2.42276 79947,0.\H,3.500153899,1.7126723299,-1.7060911123\N,1.3893012572,0.,0. \C,-0.6850311428,1.1554069723,0.\C,0.6850311428,1.1554069723,0.\C,-0.6 850311428,-1.1554069723,0.\C,0.6850311428,-1.1554069723,0.\N,-1.389301 2572,0.,0.\H,1.251079045,2.0727902987,0.\H,-1.251079045,2.0727902987,0 .\H,-1.251079045,-2.0727902987,0.\H,1.251079045,-2.0727902987,0.\B,-2. 8946558322,0.,0.\B,-3.9491840945,0.9378232072,0.9172996095\B,-3.949184 0945,0.9378232072,-0.9172996095\B,-3.9491840945,-0.9378232072,-0.91729 96095\B,-3.9491840945,-0.9378232072,0.9172996095\B,-5.4367596833,0.,1. 3083601717\B,-5.4508410151,1.3096594903,0.\B,-5.4367596833,0.,-1.30836 01717\B,-5.4508410151,-1.3096594903,0.\B,-6.5171980664,0.,0.\C,-8.0490 176435,0.,0.\N,-9.2061161267,0.,0.\H,-3.500153899,1.7126723299,1.70609 11123\H,-3.500153899,1.7126723299,-1.7060911123\H,-3.500153899,-1.7126 723299,-1.7060911123\H,-3.500153899,-1.7126723299,1.7060911123\H,-5.85 311981,0.,2.4251284885\H,-5.8774061709,2.4227679947,0.\H,-5.85311981,0 .,-2.4251284885\H,-5.8774061709,-2.4227679947,0.\\Version=ES64L-G09RevD.01\State=1-AG\HF=-957.6264364\RMSD=8.895e-10\RMSF=2.032e-06\Dipole=0.,0.,0.\Quadrupole=-266.2037895,138.1087973,128.0949922,0.,0.,0.\PG=D02H [C2(N1C1B1B1N1.N1B1B1C1N1),SG'(H4B4),SG"(C4H8B4),X(H8B8)]\\@

8. References

(1) Kapuściński, S.; Abdulmojeed, M. B.; Schafer, T. E.; Pietrzak, A.; Hietsoi, O.; Friedli, A. C.; Kaszyński, P. Photonic materials derived from the [*closo*-$B_{10}H_{10}$]$^{2-}$ anion: Tuning photophysical properties in [*closo*-$B_{10}H_8$-1-X-10-(4-Y-NC_5H_5)]$^-$, *Inorg. Chem. Front.* **2021**, *8*, 1066–1082.

(2) Kaszynski, P.; Ringstrand, B. Functinalization of boron clusters through iodonium zwitterions, *Angew. Chem. Int. Ed.* **2015**, *54*, 6576-6581.

(3) Blake, A. J.; Felloni, M.; Hubberstey, P.; Wilson, C.; Schröder, M. Triaqua(2,6-pyridinedicarboxylato)copper(II) at 150 K, *Acta Crystallogr., Sect. E: Struct. Rep. Online* **2002**, *58*, m43–m46.

(4) CrysAlisPro, Rigaku Oxford Diffraction, 2020 and 2022, Yarnton, Oxfordshire, England.

(5) Sheldrick, G. M. *SHELXT* – Integrated space-group and crystal- structure determination, *Acta Cryst., Sect. A* **2015**, *A71*, 3-8.

(6) Sheldrick, G. M. Crystal structure refinement with *SHELXL*, *Acta Cryst., Sect. C* **2015**, *C71*, 3-8.

(7) Dolomanov, O. V.; Bourhis, L. J.; Gildea, R. J.; Howard, J. A. K.; Puschmann, H. OLEX2: a complete structure solution, refinement and analysis program, *J. Appl. Cryst.* **2009**, *42*, 339-341.
